# Global disease burden of inflammatory bowel disease in women and women of childbearing age from 1990 to 2021 and its prediction to 2040

**DOI:** 10.1371/journal.pone.0331034

**Published:** 2025-09-10

**Authors:** Jiefeng Zhao, Daxing Miao, Tianbao Xiao, Tao Yang, Jiang Chen, Xiangquan Lai

**Affiliations:** 1 Department of Colorectal and Anal Surgery, The First Affiliated Hospital of Guizhou University of Traditional Chinese Medicine, Guiyang, Guizhou Province, China; 2 State Key Laboratory of Holistic Integrative Management of Gastrointestinal Cancers, Xijing Hospital of Digestive Diseases, Fourth Military Medical University, Xi’an, Shanxi Province, China; SKUMS: Shahrekord University of Medical Science, IRAN, ISLAMIC REPUBLIC OF

## Abstract

**Objective:**

The incidence of inflammatory bowel disease (IBD) peaks between the ages of 15 and 40. This age range coincides with women of childbearing age (WCBA), who face unique challenges like adverse pregnancy outcomes and heightened anxiety. Despite the rising global prevalence of IBD, particularly among younger populations, the burden of IBD among women, especially WCBA, remains underexplored.

**Methods:**

This study utilized data from the Global Burden of Disease (GBD) Study 2021 to examine the prevalence, disability-adjusted life-years (DALYs), and mortality of IBD among women and WCBA from 1990 to 2021. The estimated annual percentage change (EAPC) in age-standardized (AS) rates was calculated to quantify temporal trends. The relationship between the socio-demographic index (SDI) and AS prevalence, DALYs rate, and mortality was assessed using methodologies such as the slope index of inequality, concentration index, frontier analysis, decomposition analysis, and the Bayesian Age-Period-Cohort model.

**Results:**

From 1990 to 2021, a significant global decline was observed in the AS prevalence rate (ASPR), DALYs rate (ASDR), and mortality rate (ASMR) of IBD among women. For WCBA, the global prevalence rate decreased slightly, while the DALYs rate increased slightly, and mortality remained unchanged. Significant variations in trends were noted across different SDI and GBD regions. In 2021, the highest ASPR, ASDR, and ASMR for female IBD were reported in Australasia, high-income North America, and Western Europe, respectively. The most pronounced upward trends were observed in East Asia, Australasia, and high-income North America. China, Mauritius, and Kuwait experienced the most significant increases in prevalence, DALYs, and mortality rates among WCBA. Health inequalities across socioeconomic strata decreased, but substantial gaps remained, particularly in India.

**Conclusion:**

This study reveals a global decline in the burden of IBD among women and WCBA from 1990 to 2021, with notable regional disparities. The decreasing trends highlight the effectiveness of certain interventions and improvements in healthcare. However, the increasing burden in some regions and for certain age groups, along with significant gaps identified in frontier analysis, emphasize the need for targeted public health strategies and resource allocation to further reduce the burden of IBD among women and WCBA.

## 1 Introduction

Inflammatory bowel disease (IBD) is defined as an immune-mediated chronic intestinal inflammation [1], which is divided into ulcerative colitis, Crohn’s disease, and IBD-unclassified [2]. IBD is thought to occur in genetically susceptible individuals who are exposed to specific environmental factors that can alter the gut microbiota, ultimately leading to dysbiosis and immune dysregulation [3]. Diarrhea, abdominal discomfort, rectal bleeding, and weight loss are common symptoms among IBD patients, but inflammation is their primary characteristic [4]. Over the past few decades, IBD has become a truly global disease [5], with its incidence and prevalence increasing rapidly worldwide, particularly among younger populations [2]. It has been reported that the regions with the highest prevalence of IBD are concentrated in North America and Europe. However, since the beginning of the 21st century, the prevalence in South America, Asia, and Africa has been on a continuous increase [6].

It is worth noting that IBD patients are typically diagnosed in early adolescence or at a young age. The incidence peak of IBD occurs between the second and fourth decades of life (15–40 years old) [7], which coincides with the age range of women of childbearing age (WCBA) as defined by the World Health Organization. In fact, many WCBA have IBD [8], and approximately 50% of women with IBD may be in their reproductive years [9]. Importantly, active IBD can impair fertility; women with active Crohn’s disease have lower spontaneous conception rates, and surgical resections or pelvic inflammatory complications further reduce fertility [10–12]. During pregnancy, uncontrolled disease activity is associated with significantly higher risks of miscarriage, intrauterine growth restriction, preterm birth, low birth weight, and delivery complications [13,14]. These adverse outcomes are closely linked to maternal psychological health; up to 25% of women with IBD experience clinically significant anxiety or depression, which in turn correlates with increased disease activity and poorer pregnancy outcomes [12,15]. Medication treatment may lead to adverse pregnancy outcomes [16], and there is a contradiction between controlling the disease and their high reproductive demands [17,18]. These factors largely contribute to the high levels of pregnancy-related fear and anxiety among WCBA with the disease [19]. Study has shown that anxiety disorders are more prevalent among female IBD patients compared to the general population [20]. Inappropriate conception timing can also increase the risk of IBD exacerbation during pregnancy [21]. Moreover, the quality of life and treatment adherence of WCBA with IBD are also relatively low [22]. Collectively, these reproductive and mental-health considerations underscore the importance of targeted research into the burden of IBD specifically among WCBA.

Despite these significant health implications, the global burden of IBD among women, particularly WCBA, remains underexplored. With the increasing global female population, there is a critical need for comprehensive research to analyze the burden of IBD among women, especially WCBA, across different regions. This study aims to address this gap by utilizing data from the Global Burden of Disease (GBD) Study 2021 to examine the prevalence, DALYs, and mortality patterns of IBD among WCBA from 1990 to 2021. We hypothesize that regional disparities in IBD burden exist and that socio-economic development significantly impacts these trends.

## 2 Materials and methods

### 2.1 Data source

The GBD2021 provides an extensive examination of the impacts of 371 distinct health conditions across 204 countries and territories, covering a broad spectrum of health metrics such as disease incidence, prevalence, DALYs, mortality, risk factors, and other related indicators [23]. The compilation of data for GBD 2021 was conducted through a rigorous collection process, sourcing information from a variety of databases, including national censuses, household surveys, civil registration systems, vital statistics, disease registries, health service records, air quality monitoring systems, satellite imagery, and other relevant health data repositories [24].

### 2.2 Study population

This study focused on female individuals diagnosed with IBD from 1990 to 2021, with a particular emphasis on WCBA. Following the GBD 2021 case definition, ‘inflammatory bowel disease’ encompasses both ulcerative colitis and Crohn’s disease. WCBA were defined as females aged 15–49 years, consistent with the standard reproductive-age classification adopted by the World Health Organization in its reproductive-health indicators [25] and widely used in UN Sustainable Development Goals reporting, GBD demographic tabulations, and global maternal-health analyses. Data on the prevalence, DALYs, and mortality of IBD among women and WCBA were specifically extracted from the GBD 2021 dataset. The selection of study participants was based on the availability of comprehensive and reliable data, with no specific exclusion criteria applied.

### 2.3 Health metrics

DALYs, a key metric in this study, represent the total number of years lost due to illness, combining years of life lost (YLL) and years lived with disability (YLD). One DALY can be considered as one year of healthy life lost due to disability or premature death from a specific cause [23].For the analysis of the all-age population, we utilized the AS rates per 100,000 population as reported by GBD 2021, including the AS prevalence rate (ASPR), AS DALYs rate (ASDR), and AS mortality rate (ASMR). These metrics were chosen to provide a standardized and comparable measure of disease burden across different regions and time periods.

### 2.4 Analytical methods

To track the trends in ASR over the specified time period, we calculated the Estimated Annual Percentage Change (EAPC), a measure summarizing the average annual percent change in rates over the study period, and used linear regression models to determine the 95% confidence intervals (CI) for the EAPC [26].

### 2.5 Data access

The data used in this study can all be retrieved through the GBD database 2021, accessible via the URL http://ghdx.healthdata.org/gbd-results-tool.

### 2.6 Statistics

In this study, we utilized frontier analysis —a technique specifically designed to benchmark health system performance by identifying the ‘best achievable’ burden level at each Socio-demographic Index (SDI) quintile— to develop a frontier model based on the SDI. This approach is optimal for quantifying potential health gains under resource constraints, as it avoids the ‘average effect’ limitation of conventional regressions that mask inequities across development strata. We applied it to assess the prevalence, disability-adjusted life-years, and mortality rates, thereby enabling priority-setting for resource allocation to reduce global health disparities [27].

Additionally, we employed the Das Gupta decomposition technique to delineate the variations in the disease burden of IBD among females between 1990 and 2021, attributing these variations to the influences of aging, population growth, and epidemiological change. The selection of population growth, aging, and epidemiological change as the core factors for decomposing variations in disease burden is a well-established and widely accepted framework in epidemiological research, particularly when employing methodologies like the Das Gupta decomposition technique. This tripartite division allows for a nuanced understanding of the drivers behind changes in disease metrics over time. Percentages for the three components (population growth, population ageing, and epidemiological change) are additive contributions; a contribution >100% occurs when epidemiological change is negative (i.e., risk or case-fatality has declined) and offsets part of the population-driven increase [28,29].

The Slope Index of Inequality (SII), a regression-based measure of absolute inequality, and the concentration index, a metric derived from Lorenz curve analysis, were utilized to quantify SDI-related inequalities in the burden of IBD among WCBA across countries [30]. The SII was derived from a regression analysis that linked the DALYs of IBD among women and WCBA at the national level to SDI-based relative position scale, with the scale’s reference point set at the median of the cumulative class intervals for the SDI-ranked population. The health inequality concentration index was determined by plotting a Lorenz curve against the cumulative relative distribution of DALYs of IBD among SDI-ranked populations, followed by numerical integration of the area under the curve [31,32].

The Bayesian Age-Period-Cohort (BAPC) model, an extension of the Generalized Linear Model within a Bayesian framework, was applied to generate nuanced projections of future disease burdens by accounting for interactions among age, period, and cohort effects. Key assumptions underlying the BAPC projections include: Future age-period-cohort effects are extrapolated based on historical trends (1990–2021), assuming no structural breaks in disease drivers; The model does not incorporate unforeseeable events (e.g., pandemics, wars, or radical healthcare reforms) that may abruptly alter IBD trajectories; Interactions between age, period, and cohort are modeled as additive components, potentially underestimating synergistic effects; Case ascertainment probabilities are assumed stable across projection years, despite potential technological advances. For sensitivity analysis, we employed the Autoregressive Integrated Moving Average (ARIMA). In this study, we applied the BAPC model to project the global burden of IBD among women and WCBA from 2022 to 2040. For data processing, analysis, and figure creation, we utilized the R software package (version 4.4.2) and the JD_GBDR software (version 2.35).

### 2.7 Ethical approval

This research utilizes data from the Global Burden of Disease (GBD) study. The GBD employs deidentified data, and the University of Washington Institutional Review Board has granted a waiver of informed consent. Given that our analysis relies on this publicly available and deidentified dataset, no additional ethics approval was necessary for our specific study.

## 3 Results

### 3.1 Prevalence, DALYs and mortality rate of IBD among women and WCBA

**Fig 1:**
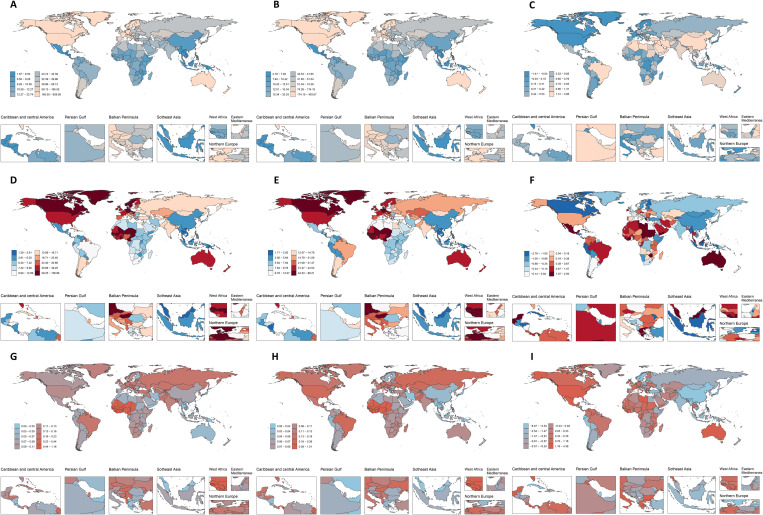
The global changes of prevalence (A-C), DALYs (D-F), and mortality (G-I) rates of IBD among WCBA from 1990 to 2021. Rates in 1990 (A, D, and **G)**; Rates in 2021 (B, E, and **H)**; Changes from 1990 to 2021 (C, F, and **I)**. Abbreviation: IBD, inflammatory bowel disease; WCBA, women of childbearing age; DALYs, disability-adjusted life-years.

In 1990, the global prevalence, DALYs rate, and mortality rate of IBD among WCBA were 45.22 per 100,000 (95% CI: 37.98 to 53.75), 14.44 per 100,000 (95% CI: 10.91 to 18.34), and 0.13 per 100,000 (95% CI: 0.08 to 0.17), respectively. In 2021, the global prevalence, DALYs rate, and mortality rate of IBD among WCBA were 44.49 per 100,000 (95% CI: 36.13 to 54.05), 14.45 per 100,000 (95% CI: 11.49 to 17.96), and 0.13 per 100,000 (95% CI: 0.10 to 0.16), respectively. Globally, the prevalence rate among WCBA is declining. Nevertheless, all five SDI regions display upward trends. Among the 21 GBD regions, 19 have experienced an increase in prevalence rate, while two regions have seen a decline: High-income North America and Oceania (S1 Table and S1A Fig). Globally, the overall DALYs rate for WCBA has shown a slight increase; however, among the five SDI regions, only the High SDI and High Middle SDI regions have seen a decline. Among the 21 GBD regions, 11 have shown an increase in the DALYs rate, while 10 have experienced a decrease (S1 Table and S1B Fig). Globally, the overall mortality rate for WCBA has remained unchanged; however, among the five SDI regions, the High Middle SDI, Middle SDI, and Low Middle SDI regions have shown a declining trend, while the High SDI region has remained stable and the Low SDI region has shown an increasing trend. Among the 21 GBD regions, five have shown an increasing trend, six have remained unchanged, and the remaining regions have shown a decreasing trend (S1 Table and S1C Fig). In China, Libya, and the Republic of Korea, the prevalence rate of WCBA has shown the most significant increases, while in Finland, Canada, and Iceland, the prevalence rate of WCBA has declined the most (S4 Table and Fig 1A–1C). Mauritius, Libya, and American Samoa have experienced the most significant increases in the DALYs rate for WCBA, whereas Estonia, Latvia, and Bermuda have seen the most significant decreases in the DALYs rate for WCBA (S5 Table and Fig 1D–1F). Kuwait, Guam, and American Samoa have shown the most significant increases in the mortality rate for WCBA, while Singapore, Estonia, and Sweden have experienced the most significant decreases in the mortality rate for WCBA (S6 Table and Fig 1G–1I).

**Fig 2:**
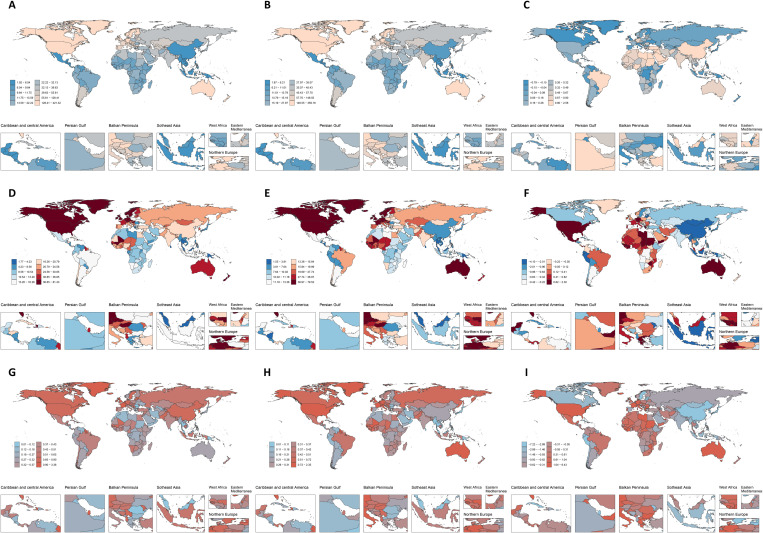
The global changes of AS prevalence, DALYs, and mortality rates of IBD among women from 1990 to 2021. AS rates in 1990 (A, D, and **G)**; AS rates in 2021 (B, E, and **H)**; EAPC from 1990 to 2021 (C, F, and **I)**. Abbreviation: AS, age-standardized; IBD, inflammatory bowel disease; DALYs, disability-adjusted life-years; EAPC, estimated annual percentage change.

The ASPR for female IBD decreased from 49.43 per 100,000 (95% CI: 43.20 to 57.62) in 1990 to 45.90 per 100,000 (95% CI: 39.71 to 53.97) in 2021, with an EAPC of −0.15 (95% CI: −0.28 to −0.02) (S2 Table, Fig 2A–2C, and S2A Fig). The ASDR for female IBD decreased from 21.18 per 100,000 (95% CI: 16.65 to 25.88) in 1990 to 17.75 per 100,000 (95% CI: 15.08 to 21.23) in 2021, with an EAPC of −0.48 (95% CI: −0.58 to −0.39) (S2 Table, Fig 2D–2F, and S2B Fig). The ASMR for female IBD decreased from 0.58 per 100,000 (95% CI: 0.46 to 0.68) in 1990 to 0.50 per 100,000 (95% CI: 0.42 to 0.59) in 2021, with an EAPC of −0.31 (95% CI: −0.52 to −0.11) (S2 Table, Fig 2G–2I, and S2C Fig). In 2021, the ASPR for female IBD in the region of Australasia was the highest, at 214.00 per 100,000 people (95% CI: 182.06 to 255.17) (S2 Table). The overall ASPR of female IBD worldwide shows a downward trend, with a decreasing trend in High middle SDI regions, an increasing trend in the remaining SDI regions, and a decreasing trend in Central Sub-Saharan Africa, Eastern Europe, and Oceania regions (S2 Table, S2A Fig). The most obvious downward trend is in the Central Sub-Saharan Africa region (EAPC of −0.36 (95% CI: −0.55 to −0.17)), and the most obvious upward trend is in the East Asia region (EAPC of 2.48 (95% CI: 1.82 to 3.14)) (S2 Table, S2A Fig). In 2021, the ASDR for female IBD in the region of High-income North America was the highest, at 51.06 per 100,000 people (95% CI: 41.65 to 62.53) (S2 Table). The overall ASDR of female IBD worldwide shows a downward trend, with an increasing trend in High SDI regions, a decreasing trend in the remaining SDI regions. The most obvious downward trend is in the East Asia region (EAPC of −3.71 (95% CI: −3.98 to −3.43)), and the most obvious upward trend is in the Australasia region (EAPC of 1.13 (95% CI: 0.79 to 1.47)) (S2 Table, S2B Fig). In 2021, the ASMR for female IBD in the region of Western Europe was the highest, at 1.10 per 100,000 people (95% CI: 0.92 to 1.21) (S2 Table). The overall ASMR of female IBD worldwide shows a downward trend, with an increasing trend in High SDI regions and an increasing trend in the remaining SDI regions. The most obvious downward trend is in the High-income Asia Pacific region (EAPC of −4.44 (95% CI: −4.83 to −4.04)), and the most obvious upward trend is in the Australasia region (EAPC of 3.82 (95% CI: 2.89 to 4.77)) (S2 Table, S2C Fig).

Fig 2 and S4 Table display the ASPR, ASDR, and ASMR for IBD among women across 204 countries and regions. The top 5 countries with the lowest ASPR for female IBD in 1990 were Mexico, Guatemala, Honduras, Cambodia, and Lao People’s Democratic Republic, while the highest were Canada, Netherlands, Iceland, Sweden, and Greenland (Fig 2A). By 2021, the top 5 countries and regions with the lowest ASPR for female IBD were Mexico, Philippines, Cambodia, Honduras, and Guatemala, and the top 5 countries and regions with the highest ASPR were Canada, Netherlands, Germany, Norway, and Sweden (Fig 2B). Fig 2C illustrates that in 49 countries and regions, the ASPR for female IBD has declined, with the most significant decrease observed in Italy. In contrast, in 155 countries and regions, the ASPR has increased, with China experiencing the most notable increase (Fig 2C).

In 1990, the top 5 countries with the lowest ASDR for female IBD were Northern Mariana Islands, Solomon Islands, Malaysia, Guam, and Papua New Guinea. The highest ASDR for female IBD was observed in Canada, Netherlands, Cyprus, Greenland, and Germany (Fig 2D). By 2021, the lowest ASDR for female IBD were in Northern Mariana Islands, Papua New Guinea, Solomon Islands, Sri Lanka, and Singapore, while the highest ASDR were in Netherlands, Germany, Canada, Greenland, and United States of America (Fig 2E). A total of 129 countries and regions have seen a decline in the ASDR for female IBD, with the most significant decrease observed in Estonia, and the most notable increase in Mauritius (Fig 2F).

In 1990, the top 5 countries with the lowest ASMR for female IBD were Northern Mariana Islands, Kuwait, Solomon Islands, Papua New Guinea, and Guam. The highest ASMR for female IBD were observed in Cyprus, Netherlands, Republic of Korea, Qatar, and Brunei Darussalam (Fig 2G). By 2021, the lowest ASMR for female IBD were in Northern Mariana Islands, Singapore, Sri Lanka, Cook Islands, and Guam, while the highest ASMR were in Netherlands, Germany, Cyprus, France, United Kingdom (Fig 2H). A total of 125 countries and regions have seen a decline in the ASDR for female IBD, with the most significant decrease observed in Singapore, and the most notable increase in Kuwait (Fig 2I).

### 3.2 Decomposition analysis

**Fig 3:**
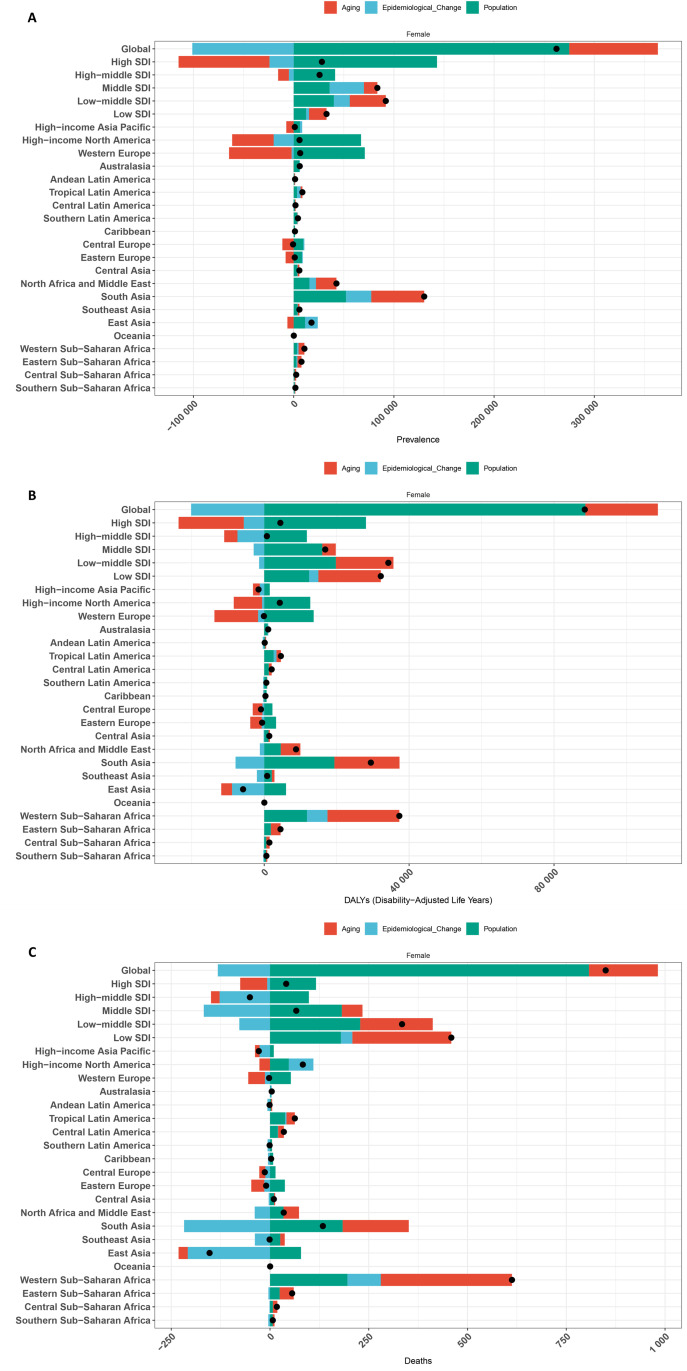
Decomposition analysis of the changes in prevalence (A), DALYs (B), and mortality (C) rates of IBD among women across global, 5 SDI and 21 GBD regions. Abbreviation: DALYs, disability-adjusted life years; IBD, inflammatory bowel disease; SDI, Socio-demographic Index; GBD, Global Burden of Disease.

Fig 3 and S3 Table present the results of a decomposition analysis of the changes in prevalence, DALYs and mortality rates for IBD in females across global, 5 SDI and 21 GBD regions. The results indicate that globally, 104.85% of the variation in the prevalence rate of IBD in females is attributed to population, followed by epidemiological change (−38.58%) (Fig 3A and S3 Table). Meanwhile, 100.17%of the variation in the DALYs rate of IBD in females is attributed to population, followed by epidemiological change (−22.8%) (Fig 3B and S3 Table). 95.02%of the variation in the mortality rate of IBD in females is attributed to population, followed by aging (20.53%) (Fig 3C and S3 Table). For prevalence, the contributions of population are positive across global and all SDI regions, Aging and Epidemiological change are positive across Middle, Low-middle, and Low SDI regions, while the impact is negative in High and High-middle SDI regions. For DALYs and mortality, the contribution of population is positive across global and all SDI regions, Aging is positive across global, Middle, Low-middle, and Low SDI regions, while the impact of Epidemiological change is negative in global, High, High-middle, Middle, and Low-middle SDI regions. For global, the High, High-middle, Middle, and Low-middle SDI regions, population are the dominant factor affecting prevalence and DALYs, while population is the dominant factor affecting mortality for global, the High, Middle, and Low-middle SDI regions. For the Low SDI region, aging is the dominant factor affecting both prevalence and DALYs, as well as mortality. The prevalence is least affected by epidemiological changes, population, and aging in Andean Latin America (0.09%), Western Sub-Saharan Africa (31.75%), and Caribbean (−1.89%), respectively. The DALYs are least affected by these same factors in Eastern Sub-Saharan Africa (−0.4%), Western Sub-Saharan Africa (31.73%), and Australasia (−2.55%). The mortality is least affected by these same factors in Central Latin America (2.03%), Western Sub-Saharan Africa (32.01%), and Australasia (0.63%). Among different regions, the prevalence rate in Central Europe (1792.61%), Central Europe (−1540.65%), and High-income North America (−342.31%) are most affected by aging, population, and epidemiological changes. Meanwhile, DALYs are most affected by these factors in Western Europe (14365.49%), Western Europe (−16264%), and Western Europe (1998.51%). The mortality rates in Western Europe (1817.72%), Southeast Asia (−2455.63%), and Andean Latin America (622.05%) are most affected by these factors.

### 3.3 Health inequalities analysis

**Fig 4:**
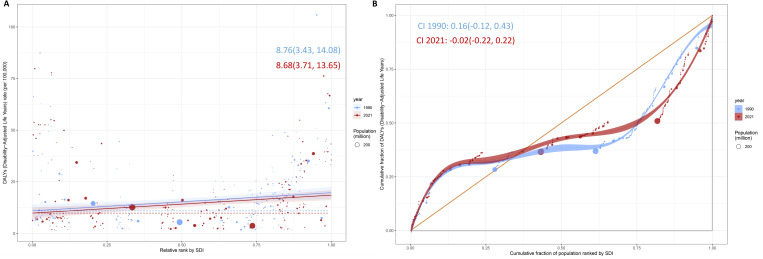
Health inequalities in the burden of IBD among WCBA. **(A)** Health inequality regression curves of DALYs rate. **(B)** Concentration curve of DALYs. Abbreviation: DALYs, disability-adjusted life years; IBD, inflammatory bowel disease; WCBA, women of childbearing age.

In 1990, the Slope Index of Inequality for health inequalities of DALYs rate in IBD among WCBA was 8.76(3.43, 14.08), and by 2021, these indices decreased to 8.68(3.71, 13.65) (Fig 4A). This signifies a notable reduction in the inequalities between the highest and lowest socioeconomic strata from 1990 to 2021. In 1990, the concentration index for health inequalities of DALYs rate in IBD among WCBA was 0.16(−0.12, 0.43), and by 2021, these indices decreased to −0.02(−0.22, 0.22) (Fig 4B). This signifies a notable reduction in the inequalities between the highest and lowest socioeconomic strata from 1990 to 2021.

### 3.4 Frontier analysis

**Fig 5:**
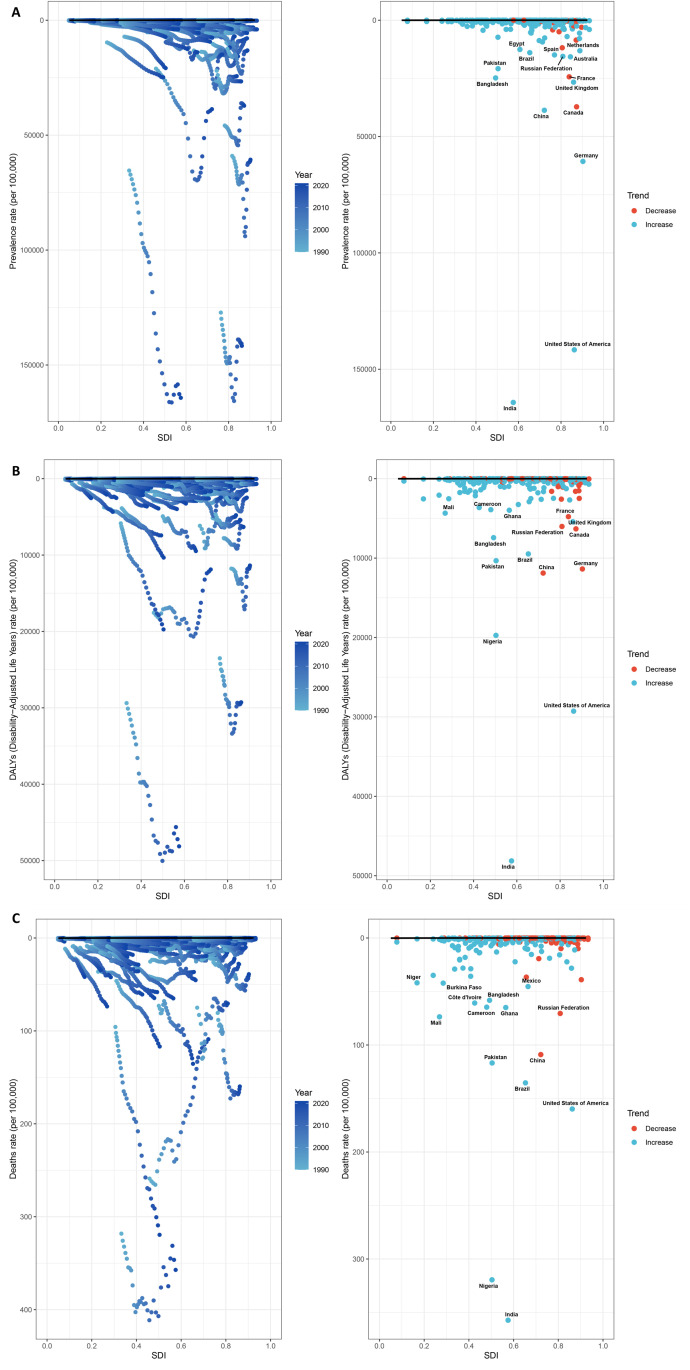
Frontier analysis for the potential for improvement in reducing the burden of IBD among WCBA across 204 countries and regions. Prevalence rate **(A)**, DALYs rate **(B)**, and mortality rate **(C)**. Abbreviation: DALYs, disability-adjusted life years; IBD, inflammatory bowel disease; WCBA, women of childbearing age.

We utilized frontier analysis to uncover the potential for improvement in reducing the burden of IBD among WCBA across different countries and regions (Fig 5). The countries and regions with the greatest actual gap in prevalence and DALYs rates for WCBA with IBD compared to the theoretical best are India, and the United States of America (Fig 5A and 5B). It is worth noting that compared to the theoretical optimal value, the countries and regions with the smallest gap in actual prevalence rates include Brazil, Egypt, the Russian Federation, Spain, the Netherlands, and Australia (Fig 5A). Meanwhile, the countries and regions with the smallest gap in actual DALYs rates are Mali, Cameroon, Ghana, France, and the United Kingdom (Fig 5B). The countries and regions with the greatest actual gap in mortality rate for WCBA with IBD compared to the theoretical best are India and Nigeria. Compared to the theoretical optimal value, the countries and regions with the smallest gap in actual mortality rate include Niger, Burkina Faso, and Mexico (Fig 5C).

### 3.5 Prediction analysis for the burden of IBD among WCBA to 2040

**Fig 6:**
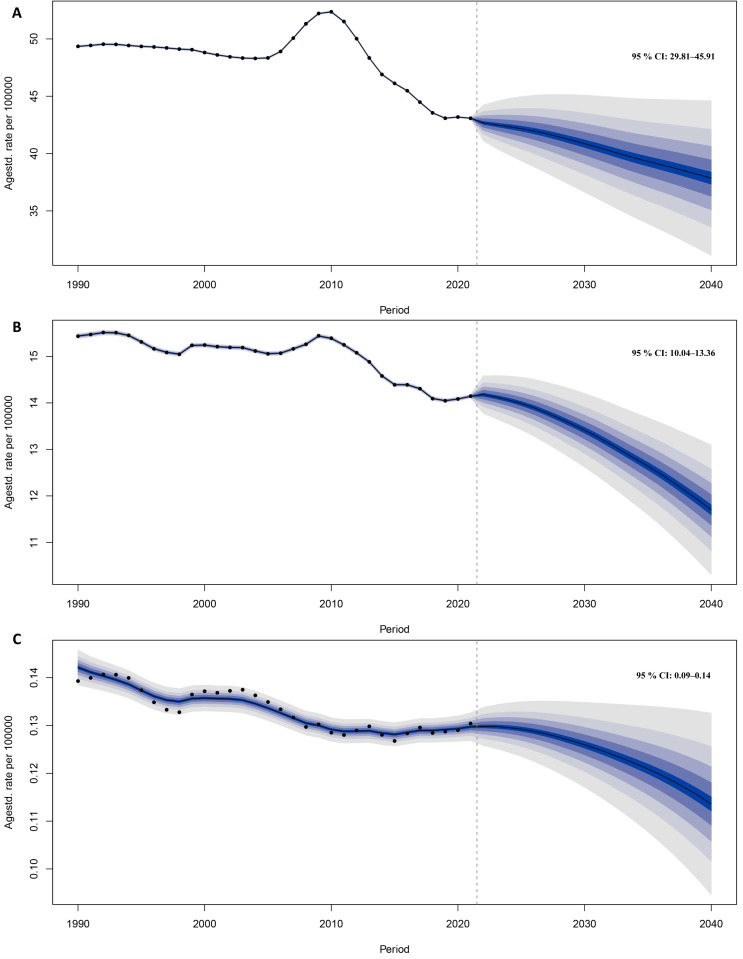
Estimated prevalence (A), DALYs (B), and mortality (C) rates for IBD among WCBA by 2040. Abbreviation: DALYs, disability-adjusted life years; IBD, inflammatory bowel disease; WCBA, women of childbearing age.

**Fig 7:**
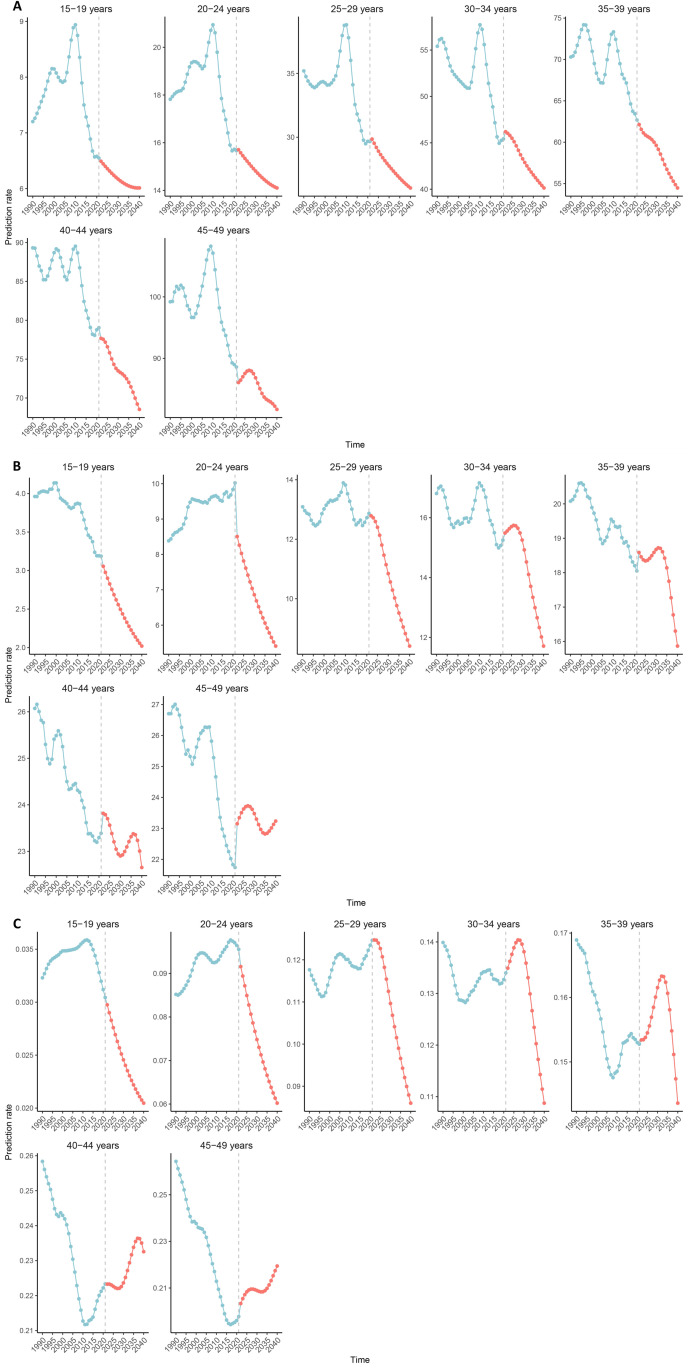
Estimated prevalence (A), DALYs (B), and mortality (C) rates of IBD in different age subgroups of WCBA by 2040. Abbreviation: DALYs, disability-adjusted life years; IBD, inflammatory bowel disease; WCBA, women of childbearing age.

The projected ASPR, ASDR and ASMR for IBD among WCBA are expected to gradually decrease through 2040 (Fig 6). In order to conduct sensitivity analysis, we used the ARIMA model, which predicts that from 2022 to 2040, the ASPR and ASDR of IBD in WCBA worldwide will generally show an upward and then downward trend, while the ASMR will generally show a downward trend (S3 Fig). It is noteworthy that for some age groups, the forecasted prevalence, DALYs and mortality rates show an increasing trend during certain periods leading up to 2040 (Fig 7). Our prediction results show that for patients aged 45–49, their predicted prevalence rate will increase from 2022 to 2027 and decrease from 2027 to 2040 (Fig 7A). The DALYs rate of patients in the 30–34 age group is expected to increase from 2022 to 2026, the DALYs rate of patients in the 35–39 age group is expected to increase from 2025 to 2031, the DALYs rate of patients in the 40–44 age group is expected to increase from 2030 to 2037, and the DALYs rate of patients in the 45–49 age group is expected to increase from 2022 to 2027 and 2035–2040 (Fig 7B). The mortality rate of patients in the 30–34 age group is expected to increase from 2022 to 2027, the mortality rate of patients in the 35–39 age group is expected to increase from 2022 to 2032, the mortality rate of patients in the 40–44 age group is expected to increase from 2027 to 2037, and the mortality rate of patients in the 45–49 age group is expected to increase from 2022 to 2028 and 2033–2040 (Fig 7C).

## 4 Discussion

Between 1990 and 2021, global ASPR, ASDR and ASMR for female IBD declined significantly. WCBA prevalence fell slightly, DALYs rose marginally, and mortality rates remained stable. Regional disparities were evident, with high-income regions like North America and Western Europe showing persistently high disease burdens, while East Asia, particularly China, demonstrated the most significant increases in prevalence but notable declines in DALYs and mortality. Population growth was the primary driver of changes in IBD burden, and health inequalities across socioeconomic strata decreased over time. Predictions to 2040 suggest an overall decline in IBD burden among WCBA, though certain age groups may experience rising prevalence, DALYs, and mortality during specific periods.

With the growth of the global female population and the rapid development of socio – economy, the disease burden of women is steadily increasing, and in some diseases, characteristics unique to women are emerging [33,34]. However, research on women’s health still primarily focuses on reproductive health topics, while studies on many other major causes that affect women’s incidence and mortality rates, and thus their disease burden, remain relatively limited [35]. As a special group of women, the disease burden of WCBA IBD still lacks targeted research. While some studies have included the WCBA group, the data they used were limited to the earlier GBD 2019 data, and there is a lack of systematic and comprehensive analysis and prediction of the disease burden of WCBA IBD [36,37]. Differently, we conducted a comprehensive and systematic study on the disease burden of WCBA IBD using the latest GBD 2021 data, and also predicted its disease burden up to 2040.

Some of our results are similar to the previous study [37], reflecting the intractable disease burden of IBD in females in high SDI regions such as the United States and Western Europe. We speculate that this may be the result of the interaction of a variety of complex factors: First, ultra-processed foods usually contain high energy density, sugar, salt, and high fat, while having low levels of fiber, protein, and micronutrients. Women are the main consumers of ultra-processed foods [38], and low fiber, high fat content, and high sugar in ultra-processed foods are high-risk factors for IBD [39–41]. The smoking rate among females in high-income countries is higher than in countries with other income levels. Europe is the continent with the highest female smoking rate globally, reaching 17.5% [42], and smoking is associated with an increased risk of IBD [43]. Direct evidence from three large prospective cohorts of women in high-income countries confirms that higher ultra-processed food intake and active smoking are independently associated with increased risks of both Crohn’s disease and ulcerative colitis [39,43–45]. In addition, studies have pointed out that in developed countries, especially among females, there is often less sun exposure due to work and lifestyle reasons [46]. Another study has pointed out that living in high-income areas such as North America and Western Europe, which are located in high-latitude zones with limited sunlight exposure, may lead to an increased incidence of IBD due to potential vitamin D deficiency [47]. One study indicated that in the age groups of 16–34 and 35–54, females receive antibiotic prescriptions 36% and 40% higher than males, respectively [48]. Notably, as one of the etiological factors of IBD, increased exposure to antibiotics may also be a reason for the higher IBD disease burden in developed countries such as the United States [49,50]. Of course, further targeted research is still needed to determine the specific causes that ultimately lead to these results.

The most pivotal finding is the divergent burden trajectory between economic strata: while high-income countries achieved modest declines in IBD prevalence among women and WCBA, low- and middle-income countries experienced alarming increases—particularly in East Asia where China’s ASPR surged by 287%. This reversal of the ‘Western disease’ paradigm signals an accelerating epidemiological transition. Over the past 30 years, residents of densely populated large cities in China have been influenced by Western society, including an increase in the intake of fat and refined sugar, as well as changes in lifestyle, such as increased smoking, reduced breastfeeding, and greater exposure to antibiotics [51,52]. In addition, China’s large population base and the increasingly serious aging problem have also led to a continuous increase in the prevalence of IBD in females [53]. With the enhanced awareness of diseases among clinicians and health practitioners, as well as advancements in medical technology, patients with IBD have gained easier access to diagnostic tests, thereby leading to a rapid increase in prevalence [54,55]. However, the significant decline in DALYs and mortality rate of IBD among women in East Asia, particularly China, contrasts with the rising burden in high-income regions, underscoring the impact of healthcare advancements and public health policies in mitigating disease severity [56,57].

We further adopted decomposition analysis to address the regret of previous studies not identifying the key factors driving changes in the global disease burden of female IBD. Unlike many disease burdens that are attributed to aging [58,59], the primary driving factor for changes in the DALYs rate, prevalence, and mortality rate of IBD in females is population. Between 1990 and 2021, the global population increased significantly [60], thereby providing a fertile ground for the increase in the burden of IBD in females. It is also important to note that, with population mobility and migration, cases from areas with a high incidence of IBD in females may spread to other regions, leading to changes in the burden of IBD in females over a wider area [61]. Unlike the horizontal effect of population mobility, the vertical effect of high population density in urban areas also has a very significant impact on the spread and influence of IBD in females [61,62], thereby affecting its disease burden. In recent years, as women’s health awareness has increased, more female patients have been able to seek medical attention and receive diagnoses in a timely manner. This has led to an increase in the diagnosis rate of IBD [63], which in turn has contributed to the burden of IBD in females to a certain extent. The disproportionately large contribution of ageing in low-SDI regions reflects two demographic realities: Firstly, these populations have a younger age structure, so even modest right-shifts in the population pyramid generate large relative increases in the number of older adults, among whom IBD incidence is highest; Second, improvements in early-life survival enlarge the pool of individuals who survive to ages at which IBD typically presents. In contrast, high-SDI countries already have an older population and have experienced smaller proportional changes in age structure during the study period, so population ageing exerts a correspondingly smaller effect on IBD burden.

We have made targeted predictions on the burden of IBD in WCBA in the future, which has not been addressed in previous studies. Overall, from 2022 to 2040, the disease burden of IBD in WCBA is expected to be alleviated. This expected relief may benefit from multiple factors: For example, novel drugs such as IL-23p19 antibodies (e.g., guselkumab and risankizumab) and JAK inhibitors (e.g., upadacitinib) have been able to better control inflammatory responses and reduce disease recurrence by more precisely targeting inflammatory mediators, thereby lowering the prevalence of IBD and even the mortality rate of severe active IBD [64]. In addition, high-resolution endoscopy, capsule endoscopy, and magnetic resonance enterography can diagnose IBD earlier and more accurately, enabling patients to receive timely treatment and reducing the risk of disease progression [65]. Nevertheless, burden trends will differ across WCBA age groups. Such as those aged 30–34, 35–39, and 40–44, although the trend of IBD burden is downward, their DALYs and mortality rates are expected to be relatively high during certain periods. This may be related to the multiple pressures that women in these age groups face in terms of childbearing, work, and family care. These pressures can lead them to more easily overlook the disease signals sent by their bodies, thereby affecting the early diagnosis and treatment of the disease [66]. Based on these results, we advocate that governments and healthcare institutions should strengthen targeted management of IBD patients in different age groups of WCBA. For example, establishing an IBD specialist clinic to facilitate patients’ consultation on their condition and treatment plans. In addition, it is necessary to develop personalized treatment and rehabilitation plans based on the patient’s life and work characteristics. This helps patients better balance treatment with daily life and work, thereby reducing the impact of the disease on their quality of life. At the same time, health promotion activities targeting WCBA should be carried out to improve the overall health literacy of this group.

The global rise in IBD-related DALYs among WCBA from 1990–2021, while DALYs fell in high-SDI regions, reflects stark disparities in healthcare capacity and demographic dynamics. High-SDI countries reduced DALYs through early diagnosis, universal access to biologics, and multidisciplinary treat-to-target protocols that minimise long-term disability [61,67]. In contrast, low- and middle-SDI settings experience diagnostic delays, limited access to advanced therapies, and fragmented care, leading to more severe disease courses and higher DALYs[6]. Decomposition analysis shows that 100% of the global DALY increase is attributable to population growth, whereas epidemiological improvements (−23%) are largely confined to high-SDI regions. Rising incidence driven by westernized diets and environmental changes further inflates DALYs where health-system resilience is weakest [68]. Methodological artefacts—variable data quality and GBD modelling assumptions—modulate but do not negate these real disparities [69].

The study underscores the need for targeted public health strategies to address the rising burden of IBD among women and WCBA in high-income regions and specific age groups. Clinicians should be aware of the increased risk among WCBA and prioritize early diagnosis and personalized treatment plans to mitigate long-term complications. The success of Chinese healthcare policies, such as the inclusion of biologics in national reimbursement programs, offers a model for other regions to improve access to effective treatments [56]. Additionally, the findings highlight the importance of addressing lifestyle factors, such as diet and smoking, which may contribute to the rising burden of IBD among women and WCBA in high-income regions [38,43]. Further research is needed to elucidate the specific mechanisms underlying the observed trends and to develop more effective prevention and treatment strategies.

Future research should explore the underlying mechanisms driving the divergent trends in IBD burden among women and WCBA across regions, particularly the role of environmental, genetic, and lifestyle factors. Longitudinal studies are needed to assess the long-term impact of public health interventions, such as dietary modifications and smoking cessation programs, on IBD burden among women. Additionally, further investigation into the impact of aging and population on IBD burden among women and WCBA is warranted, as these factors remain critical drivers of disease trends. There is also a need for targeted research on the unique challenges faced by WCBA, including the impact of IBD on pregnancy outcomes and reproductive health. Longitudinal studies and randomized controlled trials are essential to validate the findings and inform clinical guidelines.

The strengths of this study include the use of comprehensive data from the Global Burden of Disease (GBD) 2021 study, allowing for a detailed analysis of global trends. The inclusion of multiple regions and subgroups provides valuable insights into regional disparities. The inclusion of decomposition, health inequality, and frontier analyses provides a nuanced understanding of the drivers and disparities in IBD burden. However, limitations include potential data gaps in regions with less developed health information systems, which may lead to underestimation or overestimation of disease burden [70]. In regions with constrained healthcare access, underdiagnosis and misclassification of IBD are prevalent due to limited endoscopic facilities, pathological services, and specialized training. This likely leads to substantial underestimation of prevalence and DALYs in these settings, potentially masking true regional disparities. Additionally, the study assumes a relatively stable demographic structure over the projection period, which may not account for significant changes in population dynamics. The stationarity assumption may fail if emerging risk factors alter IBD pathogenesis. External shocks like COVID-19-style disruptions could invalidate trend extrapolations. Diagnostic heterogeneity across regions (e.g., endoscopy access disparities) may propagate biases into future estimates. Variations in diagnostic criteria and practices across countries may also affect the reported data. Finally, the projections for 2040 are subject to uncertainty, and the study does not consider the impact of environmental factors such as climate change [71,72]. As an ecological analysis using aggregated population-level data, this study identifies associations between SDI and IBD burden but cannot establish causality. The observed relationships may be influenced by unmeasured confounders (e.g., genetic susceptibility, microbiome profiles, or healthcare access granularity) that co-vary with socioeconomic development. While decomposition and frontier analyses quantify contributions of demographic and system-level factors, they do not imply direct causal pathways. To isolate the causal effects of the SDI from genetic and environmental confounders, we recommend Mendelian randomization studies; additionally, we suggest multi-level modeling that integrates individual-level risk factors—such as diet and pollution—with national SDI data. Because the GBD study does not provide gestational-phase-stratified burden metrics, it cannot address three key issues: (1) pregnancy-triggered disease flares; (2) the teratogenic potential of medications; and (3) neonatal outcomes. We therefore urge future GBD database to include pregnancy-specific disability weights that would allow direct quantification of the reproductive health burden.

## 5 Conclusions

While the global burden of IBD among women and WCBA declined from 1990 to 2021, interpretations require caution due to significant diagnostic bias in low-income countries, where under-ascertainment likely obscures true disease burden. Pronounced regional disparities persist, with high-income regions facing persistently high burdens, while East Asia, particularly China, demonstrates notable improvements in DALYs and mortality. To mitigate future burden, policy priorities must be region-specific: High-income regions urgently need regulatory measures targeting processed foods and antibiotic overuse; Rapidly developing economies should scale early screening and expand insurance coverage for biologics; Low-resource settings require investment in decentralized diagnostics and task-shifted IBD management. Population growth remains the primary driver of changes in IBD burden, and health inequalities have decreased over time. Predictions suggest an overall decline in IBD burden among WCBA by 2040, though certain age groups may experience rising trends. These findings highlight the need for targeted interventions and resource allocation to further reduce the burden of IBD among women and WCBA globally.

## Supporting information

S1 Fig The changes of prevalence (A), DALYs (B), and mortality (C) rates of IBD among WCBA in 21 GBD and 5 SDI regions from 1990 to 2021. Abbreviation: IBD, inflammatory bowel disease; WCBA, women of childbearing age; DALYs, disability-adjusted life-years; SDI, Socio-demographic Index; GBD, Global Burden of Disease.
(PDF)

S2 Fig The EAPC of AS prevalence (A), DALYs (B), and mortality (C) rates of IBD among women in 21 GBD and 5 SDI regions from 1990 to 2021. Abbreviation: IBD, inflammatory bowel disease; DALYs, disability-adjusted life-years; SDI, Socio-demographic Index; GBD, Global Burden of Disease; EAPC, estimated annual percentage change.
(PDF)

S3 Fig Estimated prevalence (A), DALYs (B), and mortality (C) rates for IBD among WCBA by 2040 using Autoregressive Integrated Moving Average model. Abbreviation: DALYs, disability-adjusted life years; IBD, inflammatory bowel disease; WCBA, women of childbearing age.
(PDF)

S1 Table The changes of prevalence, DALYs and mortality rate of IBD among WCBA from 1990 to 2021. Abbreviations: IBD, inflammatory bowel disease; WCBA, women of childbearing age; SDI, Socio-demographic Index; DALYs, disability-adjusted life-years; EAPC, estimated annual percentage change.
(DOCX)

S2 Table The changes of AS prevalence, DALYs and mortality rate of IBD among women from 1990 to 2021. Abbreviations: IBD, inflammatory bowel disease; AS, age-standardized; SDI, Socio-demographic Index; DALYs, disability-adjusted life-years; EAPC, estimated annual percentage change.
(DOCX)

S3 Table Decomposition analysis of the changes in prevalence, DALYs and mortality rates for IBD in women. Abbreviations: DALYs, disability-adjusted life years; IBD, inflammatory bowel disease; SDI, Socio-demographic Index.
(DOCX)

S4 Table The prevalence rate for IBD among women and WCBA and its temporal trends from 1990 to 2021 across 204 countries and regions. Abbreviations: IBD, inflammatory bowel disease; AS, age-standardized; WCBA, women of childbearing age; EAPC, estimated annual percentage change; CI, Confidence Interval.
(DOCX)

S5 Table The DALYs rate for IBD among women and WCBA and its temporal trends from 1990 to 2021 across 204 countries and regions. Abbreviations: IBD, inflammatory bowel disease; AS, age-standardized; WCBA, women of childbearing age; DALYs, disability-adjusted life-years; EAPC, estimated annual percentage change; CI, Confidence Interval.
(DOCX)

S6 Table The mortality rate for IBD among women and WCBA and its temporal trends from 1990 to 2021 across 204 countries and regions. Abbreviations: IBD, inflammatory bowel disease; AS, age-standardized; WCBA, women of childbearing age; EAPC, estimated annual percentage change; CI, Confidence Interval.
(DOCX)

## References

[1] Flynn S, Eisenstein S. Inflammatory Bowel Disease Presentation and Diagnosis. Surg Clin North Am. 2019;99(6):1051–62. doi: 10.1016/j.suc.2019.08.001 31676047

[2] Vuijk SA, Camman AE, de Ridder L. Considerations in Paediatric and Adolescent Inflammatory Bowel Disease. J Crohns Colitis. 2024;18(Supplement_2):ii31–45. doi: 10.1093/ecco-jcc/jjae087 39475081 PMC11523044

[3] Khor B, Gardet A, Xavier RJ. Genetics and pathogenesis of inflammatory bowel disease. Nature. 2011;474(7351):307–17. doi: 10.1038/nature10209 21677747 PMC3204665

[4] Rosen MJ, Dhawan A, Saeed SA. Inflammatory Bowel Disease in Children and Adolescents. JAMA Pediatr. 2015;169(11):1053–60. doi: 10.1001/jamapediatrics.2015.1982 26414706 PMC4702263

[5] Caron B, Honap S, Peyrin-Biroulet L. Epidemiology of Inflammatory Bowel Disease across the Ages in the Era of Advanced Therapies. J Crohns Colitis. 2024;18(Supplement_2):ii3–15. doi: 10.1093/ecco-jcc/jjae082 39475082 PMC11522978

[6] Ng SC, Shi HY, Hamidi N, Underwood FE, Tang W, Benchimol EI, et al. Worldwide incidence and prevalence of inflammatory bowel disease in the 21st century: a systematic review of population-based studies. Lancet. 2017;390(10114):2769–78. doi: 10.1016/S0140-6736(17)32448-0 29050646

[7] Selinger C, Laube R, Limdi JK, Headley K, Kent A, Kok K, et al. Appropriateness of small molecule agents for patients with IBD of childbearing age - a RAND/UCLA appropriateness panel. Therap Adv Gastroenterol. 2024;17. doi: 10.1177/17562848241299737 39539488 PMC11558739

[8] Selinger CP, Steed H, Purewal S, Homer R, Nihr BioResource, Brookes M. Factors Associated with Family Planning Status and Voluntary Childlessness in Women of Childbearing Age with Inflammatory Bowel Diseases. J Clin Med. 2023;12(13):4267. doi: 10.3390/jcm12134267 37445302 PMC10342358

[9] Crocetti E, Bergamaschi W, Russo AG. Population-based incidence and prevalence of inflammatory bowel diseases in Milan (Northern Italy), and estimates for Italy. Eur J Gastroenterol Hepatol. 2021;33(1S Suppl 1):e383–9. doi: 10.1097/MEG.0000000000002107 33784448 PMC8734622

[10] Carini F, Margherita Mazzola, Gagliardo C, Scaglione M, Giammanco M, Tomasello G. Inflammatory bowel disease and infertility: analysis of literature and future perspectives. Acta Biomed. 2021;92(5):e2021264. doi: 10.23750/abm.v92i5.11100 34738579 PMC8689300

[11] Ronchetti C, Cirillo F, Di Segni N, Cristodoro M, Busnelli A, Levi-Setti PE. Inflammatory Bowel Disease and Reproductive Health: From Fertility to Pregnancy-A Narrative Review. Nutrients. 2022;14(8):1591. doi: 10.3390/nu14081591 35458153 PMC9026369

[12] Alstead E. Fertility and pregnancy in inflammatory bowel disease. World J Gastroenterol. 2001;7(4):455–9. doi: 10.3748/wjg.v7.i4.455 11819810 PMC4688654

[13] Tunney R, Liu E, Limdi JK. Pregnancy outcomes among women with inflammatory bowel disease: A UK tertiary centre experience. Indian J Gastroenterol. 2024. doi: 10.1007/s12664-024-01657-4 39222194 PMC13009036

[14] Shmidt E, Dubinsky MC. Inflammatory Bowel Disease and Pregnancy. Am J Gastroenterol. 2022;117(10S):60–8. doi: 10.14309/ajg.0000000000001963 36194035

[15] Hinnant L, Rios Villacorta N, Chen E, Bacchus D, Dotson J, Greywoode R, et al. Consensus Statement on Managing Anxiety and Depression in Individuals with Inflammatory Bowel Disease. Inflamm Bowel Dis. 2025;31(5):1248–55. doi: 10.1093/ibd/izae151 39173019 PMC12069991

[16] N M-S, Álvarez-Troncoso J, Robles-Marhuenda Á, De la Calle Fernández-Miranda M, Muner Hernando ML, Bartha JL. Safety of biologic immunosuppressants in pregnant women with immune-mediated inflammatory diseases. J Autoimmun. 2024;148:103301. doi: 10.1016/j.jaut.2024.103301 39141986

[17] Monfared N, Gold M, Carbery I, Laube R, Selinger CP. Reproductive Safety Issues of Novel Small Molecules for the Treatment of Inflammatory Bowel Disease: A Systematic Review. J Clin Med. 2023;13(1):34. doi: 10.3390/jcm13010034 38202041 PMC10780022

[18] Liu E, Laube R, Leong RW, Fraser A, Selinger C, Limdi JK. Managing Inflammatory Bowel Disease in Pregnancy: Health Care Professionals’ Involvement, Knowledge, and Decision Making. Inflammatory Bowel Diseases. 2022;29(4):522–30. doi: 10.1093/ibd/izac101 35713620

[19] Homer-Perry R, Czuber-Dochan W, Wade T, Purewal S, Chapman SCE, Brookes M, et al. Full title: “Hopes, worries and expectations” experiences of pregnancy with inflammatory bowel disease: An interpretative phenomenological analysis study. Heliyon. 2024;10(11):e31954. doi: 10.1016/j.heliyon.2024.e31954 PMC11167349 38868041

[20] Javaid SF, Hashim IJ, Hashim MJ, Stip E, Samad MA, Ahbabi AA. Epidemiology of anxiety disorders: global burden and sociodemographic associations. Middle East Curr Psychiatry. 2023;30(1). doi: 10.1186/s43045-023-00315-3

[21] Laube R, Paramsothy S, Leong RW. Review of pregnancy in Crohn’s disease and ulcerative colitis. Therap Adv Gastroenterol. 2021;14:17562848211016242. doi: 10.1177/17562848211016242 34046084 PMC8135214

[22] Selinger CP, Laube R, Steed H, Brookes M, BioResource N, Leong RWL. Planning to conceive within a year is associated with better pregnancy-specific disease-related patient knowledge and better medication adherence in women of childbearing age with inflammatory bowel disease. Therap Adv Gastroenterol. 2023;16. doi: 10.1177/17562848231193211 37667806 PMC10475232

[23] GBD 2021 Diseases and Injuries Collaborators. Global incidence, prevalence, years lived with disability (YLDs), disability-adjusted life-years (DALYs), and healthy life expectancy (HALE) for 371 diseases and injuries in 204 countries and territories and 811 subnational locations, 1990-2021: a systematic analysis for the Global Burden of Disease Study 2021. Lancet. 2024;403(10440):2133–61. doi: 10.1016/S0140-6736(24)00757-8 38642570 PMC11122111

[24] GBD 2019 Diseases and Injuries Collaborators. Global burden of 369 diseases and injuries in 204 countries and territories, 1990-2019: a systematic analysis for the Global Burden of Disease Study 2019. Lancet. 2020;396(10258):1204–22. doi: 10.1016/S0140-6736(20)30925-9 33069326 PMC7567026

[25] World Health Organization Women of reproductive age (15-49 years) population (thousands) https://www.who.int/data/gho/indicator-metadata-registry/imr-details/women-of-reproductive-age-(15-49-years)-population-(thousands).

[26] Hankey BF, Ries LA, Kosary CL, Feuer EJ, Merrill RM, Clegg LX, et al. Partitioning linear trends in age-adjusted rates. Cancer Causes Control. 2000;11(1):31–5. doi: 10.1023/a:1008953201688 10680727

[27] GBD 2019 Under-5 Mortality Collaborators. Global, regional, and national progress towards Sustainable Development Goal 3.2 for neonatal and child health: all-cause and cause-specific mortality findings from the Global Burden of Disease Study 2019. Lancet. 2021;398(10303):870–905. doi: 10.1016/S0140-6736(21)01207-1 34416195 PMC8429803

[28] Das Gupta P. A general method of decomposing a difference between two rates into several components. Demography. 1978;15(1):99–112. doi: 10.2307/2060493 631402

[29] Zhang Z, Du N, Xu C-M, Chen W, Ting-Ting-Chen, Xiao Y. Global, regional, and national burden of inflammatory bowel disease in persons aged 60-89 years from 1992 to 2021. BMC Gastroenterol. 2025;25(1):425. doi: 10.1186/s12876-025-04042-3 40457222 PMC12131378

[30] Zhao J, Zhu J, Huang C, Zhu X, Zhu Z, Wu Q, et al. Uncovering the information immunology journals transmitted for COVID-19: A bibliometric and visualization analysis. Front Immunol. 2022;13:1035151. doi: 10.3389/fimmu.2022.1035151 36405695 PMC9670819

[31] Cao F, He Y-S, Wang Y, Zha C-K, Lu J-M, Tao L-M, et al. Global burden and cross-country inequalities in autoimmune diseases from 1990 to 2019. Autoimmun Rev. 2023;22(6):103326. doi: 10.1016/j.autrev.2023.103326 36958621

[32] Jiang C-Y, Han K, Yang F, Yin S-Y, Zhang L, Liang B-Y, et al. Global, regional, and national prevalence of hearing loss from 1990 to 2019: A trend and health inequality analyses based on the Global Burden of Disease Study 2019. Ageing Res Rev. 2023;92:102124. doi: 10.1016/j.arr.2023.102124 37972859

[33] Dávila-Cervantes CA, Agudelo-Botero M. Revealing the burden of chronic kidney disease in Mexican women, 1990-2021. BMC Nephrol. 2024;25(1):346. doi: 10.1186/s12882-024-03797-3 39394115 PMC11470595

[34] Zhu B, Hu S, Guo J, Dong Z, Dong Y, Li F. Differences in the global exposure, mortality and disability of low bone mineral density between men and women: the underestimated burden in men. BMC Public Health. 2023;23(1):991. doi: 10.1186/s12889-023-15947-7 37248448 PMC10226255

[35] Hallam L, Vassallo A, Pinho-Gomes A-C, Carcel C, Woodward M. Does Journal Content in the Field of Women’s Health Represent Women’s Burden of Disease? A Review of Publications in 2010 and 2020. J Womens Health (Larchmt). 2022;31(5):611–9. doi: 10.1089/jwh.2021.0425 35333604 PMC9133969

[36] Cao F, He Y-S, Sang N, Liu Y-C, Hu X, Ni Q-Y, et al. Age-standardized incidence, prevalence, and mortality rates of autoimmune diseases in women of childbearing age from 1990 to 2019. Autoimmun Rev. 2023;22(11):103450. doi: 10.1016/j.autrev.2023.103450 37741529

[37] Danpanichkul P, Duangsonk K, Lopimpisuth C, Ho AH-Y, Fangsaard P, Sukphutanan B, et al. Geographical and sociodemographic epidemiology of inflammatory bowel disease in young females from 2010 to 2019. Dig Liver Dis. 2025;57(1):190–7. doi: 10.1016/j.dld.2024.07.007 39068136

[38] Hosseinpour-Niazi S, Niknam M, Amiri P, Mirmiran P, Ainy E, Izadi N, et al. The association between ultra-processed food consumption and health-related quality of life differs across lifestyle and socioeconomic strata. BMC Public Health. 2024;24(1):1955. doi: 10.1186/s12889-024-19351-7 39039502 PMC11265477

[39] Ananthakrishnan AN, Khalili H, Konijeti GG, Higuchi LM, de Silva P, Korzenik JR, et al. A prospective study of long-term intake of dietary fiber and risk of Crohn’s disease and ulcerative colitis. Gastroenterology. 2013;145(5):970–7. doi: 10.1053/j.gastro.2013.07.050 23912083 PMC3805714

[40] Suez J, Korem T, Zeevi D, Zilberman-Schapira G, Thaiss CA, Maza O, et al. Artificial sweeteners induce glucose intolerance by altering the gut microbiota. Nature. 2014;514(7521):181–6. doi: 10.1038/nature13793 25231862

[41] Ananthakrishnan AN. Environmental risk factors for inflammatory bowel diseases: a review. Dig Dis Sci. 2015;60(2):290–8. doi: 10.1007/s10620-014-3350-9 25204669 PMC4304948

[42] Jafari A, Rajabi A, Gholian-Aval M, Peyman N, Mahdizadeh M, Tehrani H. National, regional, and global prevalence of cigarette smoking among women/females in the general population: a systematic review and meta-analysis. Environ Health Prev Med. 2021;26(1):5. doi: 10.1186/s12199-020-00924-y 33419408 PMC7796590

[43] Higuchi LM, Khalili H, Chan AT, Richter JM, Bousvaros A, Fuchs CS. A prospective study of cigarette smoking and the risk of inflammatory bowel disease in women. Am J Gastroenterol. 2012;107(9):1399–406. doi: 10.1038/ajg.2012.196 22777340 PMC3667663

[44] Narula N, Wong ECL, Dehghan M, Mente A, Rangarajan S, Lanas F, et al. Association of ultra-processed food intake with risk of inflammatory bowel disease: prospective cohort study. BMJ. 2021;374:n1554. doi: 10.1136/bmj.n1554 34261638 PMC8279036

[45] Lopes EW, Chan SSM, Song M, Ludvigsson JF, Håkansson N, Lochhead P, et al. Lifestyle factors for the prevention of inflammatory bowel disease. Gut. 2022. doi: 10.1136/gutjnl-2022-328174 36591609 PMC10241983

[46] Shahudin NN, Sameeha MJ, Mat Ludin AF, Manaf ZA, Chin K-Y, Jamil NA. Barriers towards Sun Exposure and Strategies to Overcome These Barriers in Female Indoor Workers with Insufficient Vitamin D: A Qualitative Approach. Nutrients. 2020;12(10):2994. doi: 10.3390/nu12102994 33007799 PMC7599895

[47] Lin D, Jin Y, Shao X, Xu Y, Ma G, Jiang Y, et al. Global, regional, and national burden of inflammatory bowel disease, 1990-2021: Insights from the global burden of disease 2021. Int J Colorectal Dis. 2024;39(1):139. doi: 10.1007/s00384-024-04711-x 39243331 PMC11380638

[48] Gautron J, Tu Thanh G, Barasa V, Voltolina G. Using intersectionality to study gender and antimicrobial resistance in low- and middle-income countries. Health policy and planning. 2023;38(9):1017–32. doi: 10.1093/heapol/czad054 37599460 PMC10566319

[49] Zou Y, Wu L, Xu W, Zhou X, Ye K, Xiong H, et al. Correlation between antibiotic use in childhood and subsequent inflammatory bowel disease: a systematic review and meta-analysis. Scand J Gastroenterol. 2020;55(3):301–11. doi: 10.1080/00365521.2020.1737882 32180472

[50] Jackson C, Hsia Y, Bielicki JA, Ellis S, Stephens P, Wong ICK, et al. Estimating global trends in total and childhood antibiotic consumption, 2011-2015. BMJ Glob Health. 2019;4(1):e001241. doi: 10.1136/bmjgh-2018-001241 30899565 PMC6407570

[51] Ng SC, Tang W, Leong RW, Chen M, Ko Y, Studd C, et al. Environmental risk factors in inflammatory bowel disease: a population-based case-control study in Asia-Pacific. Gut. 2015;64(7):1063–71. doi: 10.1136/gutjnl-2014-307410 25217388

[52] Cui G, Liu H, Xu G, Laugsand J-B, Pang Z. Exploring Links Between Industrialization, Urbanization, and Chinese Inflammatory Bowel Disease. Front Med (Lausanne). 2021;8:757025. doi: 10.3389/fmed.2021.757025 34778319 PMC8581156

[53] Zhang Y, Liu J, Han X, Jiang H, Zhang L, Hu J, et al. Long-term trends in the burden of inflammatory bowel disease in China over three decades: A joinpoint regression and age-period-cohort analysis based on GBD 2019. Front Public Health. 2022;10:994619. doi: 10.3389/fpubh.2022.994619 36159285 PMC9490087

[54] Muzammil MA, Fariha F, Patel T, Sohail R, Kumar M, Khan E, et al. Advancements in Inflammatory Bowel Disease: A Narrative Review of Diagnostics, Management, Epidemiology, Prevalence, Patient Outcomes, Quality of Life, and Clinical Presentation. Cureus. 2023;15(6):e41120. doi: 10.7759/cureus.41120 37519622 PMC10382792

[55] Dou Z, Zheng H, Shi Y, Li Y, Jia J. Analysis of global prevalence, DALY and trends of inflammatory bowel disease and their correlations with sociodemographic index: Data from 1990 to 2019. Autoimmun Rev. 2024;23(11):103655. doi: 10.1016/j.autrev.2024.103655 39366514

[56] Yang H, Qian J. Epidemiological research, burden, and clinical advances of inflammatory bowel disease in China. Chin Med J (Engl). 2024;137(9):1009–11. doi: 10.1097/CM9.0000000000003064 38704618 PMC11062669

[57] Lv H, Jin M, Zhang H, Chen X, Wu M, Guo M, et al. Increasing newly diagnosed inflammatory bowel disease and improving prognosis in China: a 30-year retrospective study from a single centre. BMC Gastroenterol. 2020;20(1):377. doi: 10.1186/s12876-020-01527-1 33183228 PMC7659043

[58] Han T, Wang T, Ye Y, Ying C, Wang X, Liu S. The global, regional, and national burden of paralytic ileus and intestinal obstruction, 1990 to 2021: a cross-sectional analysis from the 2021 global burden of disease study. Int J Surg. 2025;111(2):1773–87. doi: 10.1097/JS9.0000000000002189 39784557

[59] Cheng X, Yang Y, Schwebel DC, Liu Z, Li L, Cheng P, et al. Population ageing and mortality during 1990-2017: A global decomposition analysis. PLoS medicine. 2020;17(6):e1003138. doi: 10.1371/journal.pmed.1003138 PMC7279585 32511229

[60] Wang S, Dong Z, Wan X. Global, regional, and national burden of inflammatory bowel disease and its associated anemia, 1990 to 2019 and predictions to 2050: An analysis of the global burden of disease study 2019. Autoimmun Rev. 2024;23(3):103498. doi: 10.1016/j.autrev.2023.103498 38052263

[61] Lin D, Jin Y, Shao X, Xu Y, Ma G, Jiang Y, et al. Global, regional, and national burden of inflammatory bowel disease, 1990-2021: Insights from the global burden of disease 2021. Int J Colorectal Dis. 2024;39(1):139. doi: 10.1007/s00384-024-04711-x 39243331 PMC11380638

[62] Zhou J-L, Bao J-C, Liao X-Y, Chen Y-J, Wang L-W, Fan Y-Y, et al. Trends and projections of inflammatory bowel disease at the global, regional and national levels, 1990-2050: a bayesian age-period-cohort modeling study. BMC Public Health. 2023;23(1):2507. doi: 10.1186/s12889-023-17431-8 38097968 PMC10722679

[63] McDermott E, Healy G, Mullen G, Keegan D, Byrne K, Guerandel A, et al. Patient Education in Inflammatory Bowel Disease: A Patient-Centred, Mixed Methodology Study. J Crohns Colitis. 2018;12(4):419–24. doi: 10.1093/ecco-jcc/jjx175 29293956

[64] Kobayashi T, Hibi T. Improving IBD outcomes in the era of many treatment options. Nat Rev Gastroenterol Hepatol. 2023;20(2):79–80. doi: 10.1038/s41575-022-00738-z 36635556 PMC9838431

[65] Kumar S, De Kock I, Blad W, Hare R, Pollok R, Taylor SA. Magnetic Resonance Enterography and Intestinal Ultrasound for the Assessment and Monitoring of Crohn’s Disease. J Crohns Colitis. 2024;18(9):1450–63. doi: 10.1093/ecco-jcc/jjae042 38554104 PMC11369078

[66] Vedavati P, Gabriela FG, Alejandra A, Jack C, Erin D, Molly EH. Differences across the lifespan between females and males in the top 20 causes of disease burden globally: a systematic analysis of the Global Burden of Disease Study 2021. The Lancet Public Health. 2024. 10.1016/S2468-2667(24)00053-7 PMC11080072 38702093

[67] Torres J, Bonovas S, Doherty G, Kucharzik T, Gisbert JP, Raine T, et al. ECCO Guidelines on Therapeutics in Crohn’s Disease: Medical Treatment. J Crohns Colitis. 2020;14(1):4–22. doi: 10.1093/ecco-jcc/jjz180 31711158

[68] Kaplan GG, Ng SC. Understanding and Preventing the Global Increase of Inflammatory Bowel Disease. Gastroenterology. 2017;152(2):313–321.e2. doi: 10.1053/j.gastro.2016.10.020 27793607

[69] Murray CJL, Vos T, Lozano R, Naghavi M, Flaxman AD, Michaud C, et al. Disability-adjusted life years (DALYs) for 291 diseases and injuries in 21 regions, 1990-2010: a systematic analysis for the Global Burden of Disease Study 2010. Lancet. 2012;380(9859):2197–223. doi: 10.1016/S0140-6736(12)61689-4 23245608

[70] Druvefors E, Landerholm K, Hammar U, Myrelid P, Andersson RE. Impaired Fertility in Women With Inflammatory Bowel Disease: A National Cohort Study From Sweden. J Crohns Colitis. 2021;15(3):383–90. doi: 10.1093/ecco-jcc/jjaa191 32949133 PMC7944497

[71] Duijvestein M, Sidhu R, Zimmermann K, Carrington EV, Hann A, Sousa P, et al. The United European Gastroenterology green paper-climate change and gastroenterology. United European Gastroenterol J. 2024;12(9):1292–305. doi: 10.1002/ueg2.12698 39452615 PMC11578853

[72] Leddin D. The Impact of Climate Change, Pollution, and Biodiversity Loss on Digestive Health and Disease. Gastro Hep Adv. 2024;3(4):519–34. doi: 10.1016/j.gastha.2024.01.018 39131722 PMC11307547

