# Supplementary figures and images for: Global disease burden of inflammatory bowel disease in women and women of childbearing age from 1990 to 2021 and its prediction to 2040
Source: PLoS One. 2025 Sep 10;20(9):e0331034. doi: 10.1371/journal.pone.0331034 (PMC12422439; doi:10.1371/journal.pone.0331034)

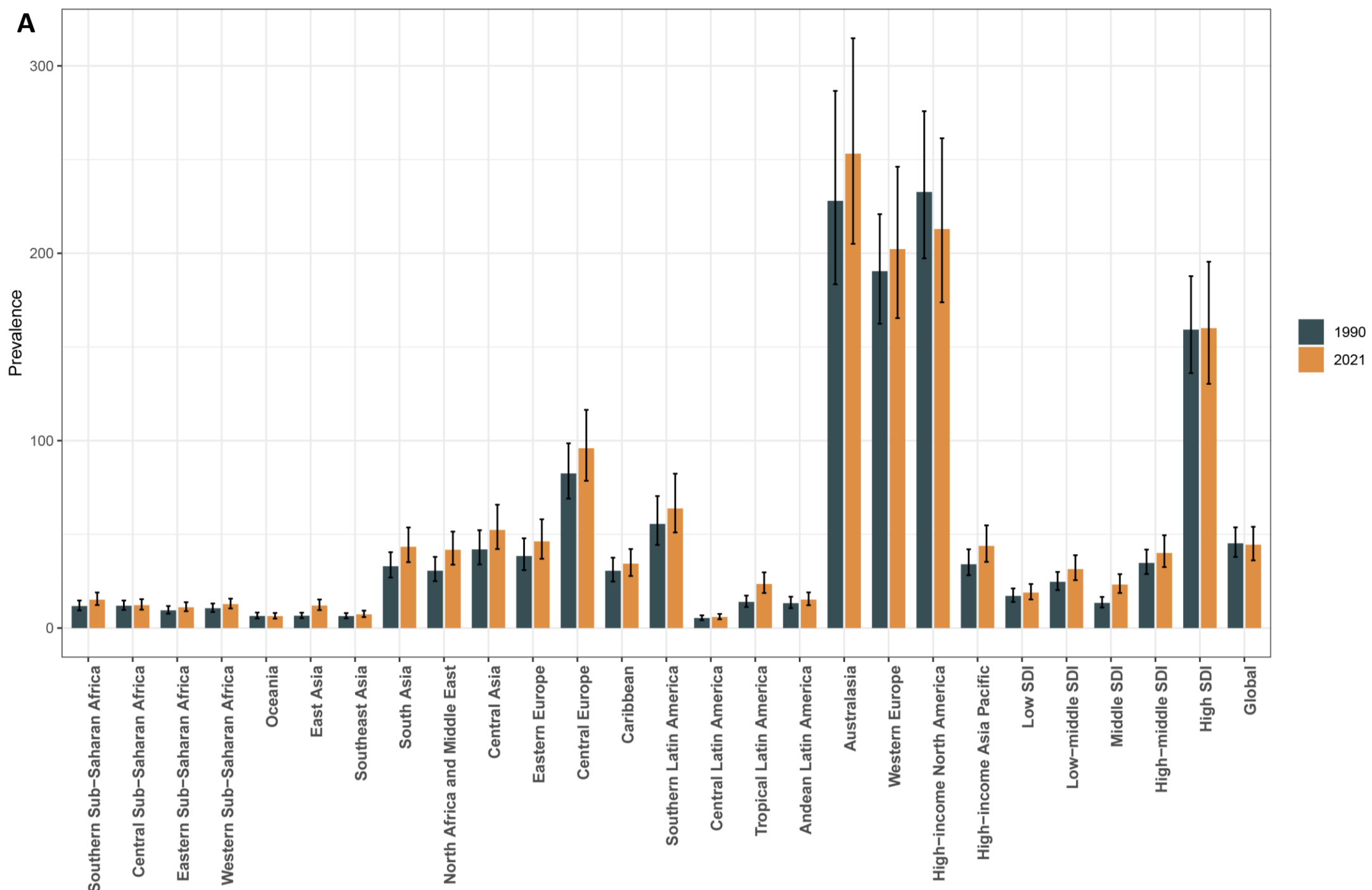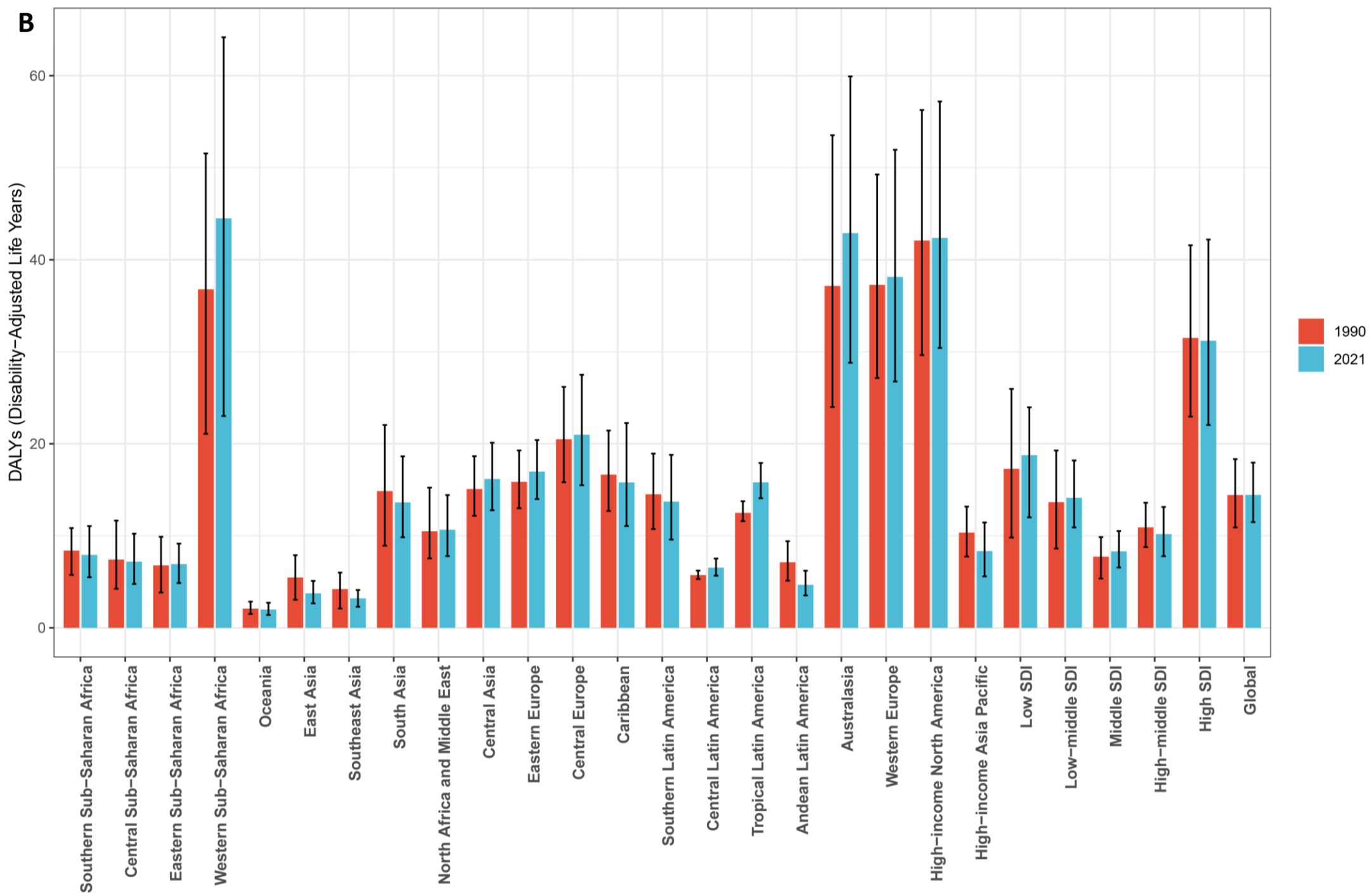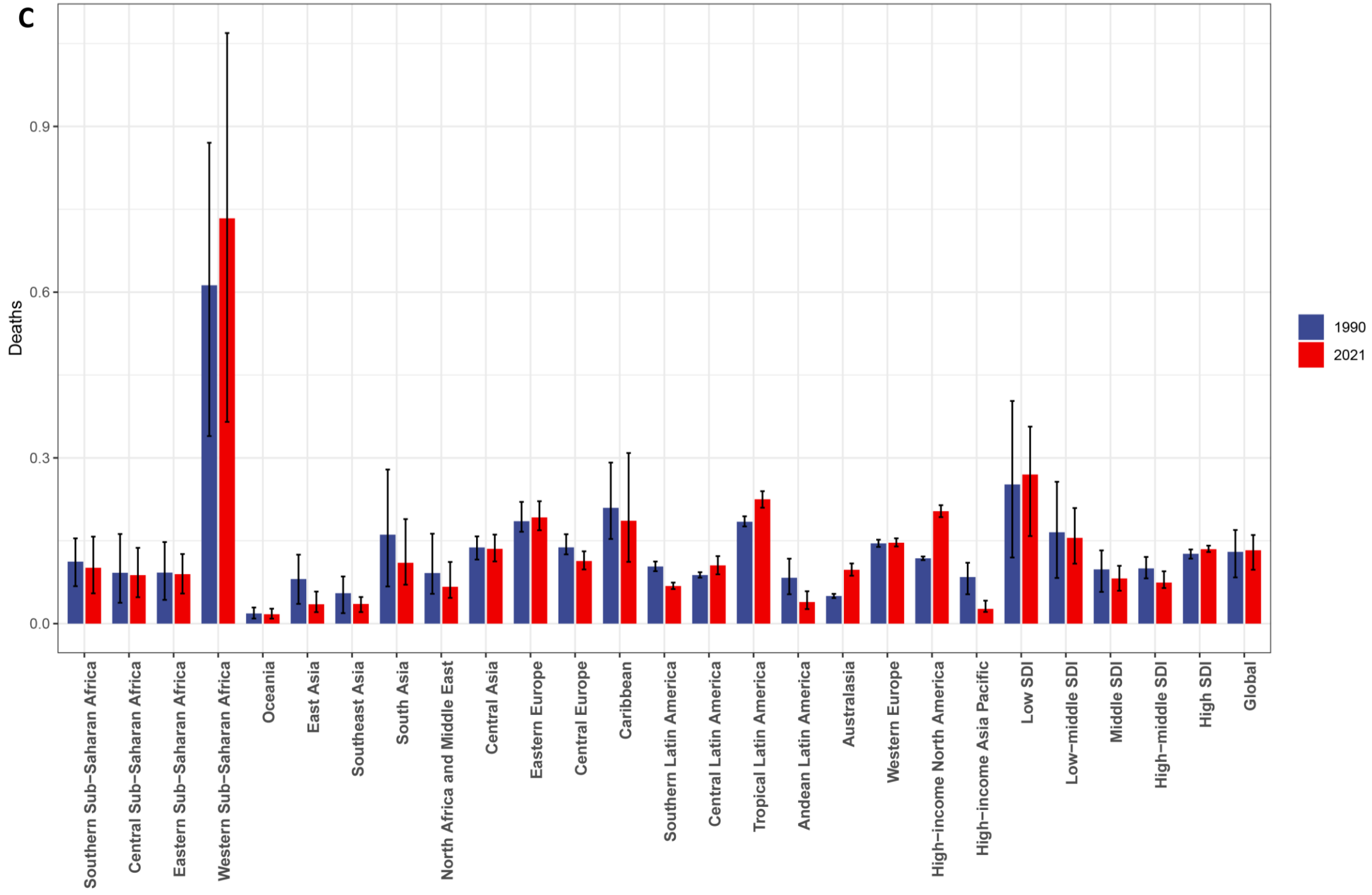

Supplement: S1 Fig — Abbreviation: IBD, inflammatory bowel disease; WCBA, women of childbearing age; DALYs, disability-adjusted life-years; SDI, Socio-demographic Index; GBD, Global Burden of Disease. (PDF) [file pone.0331034.s001.pdf]

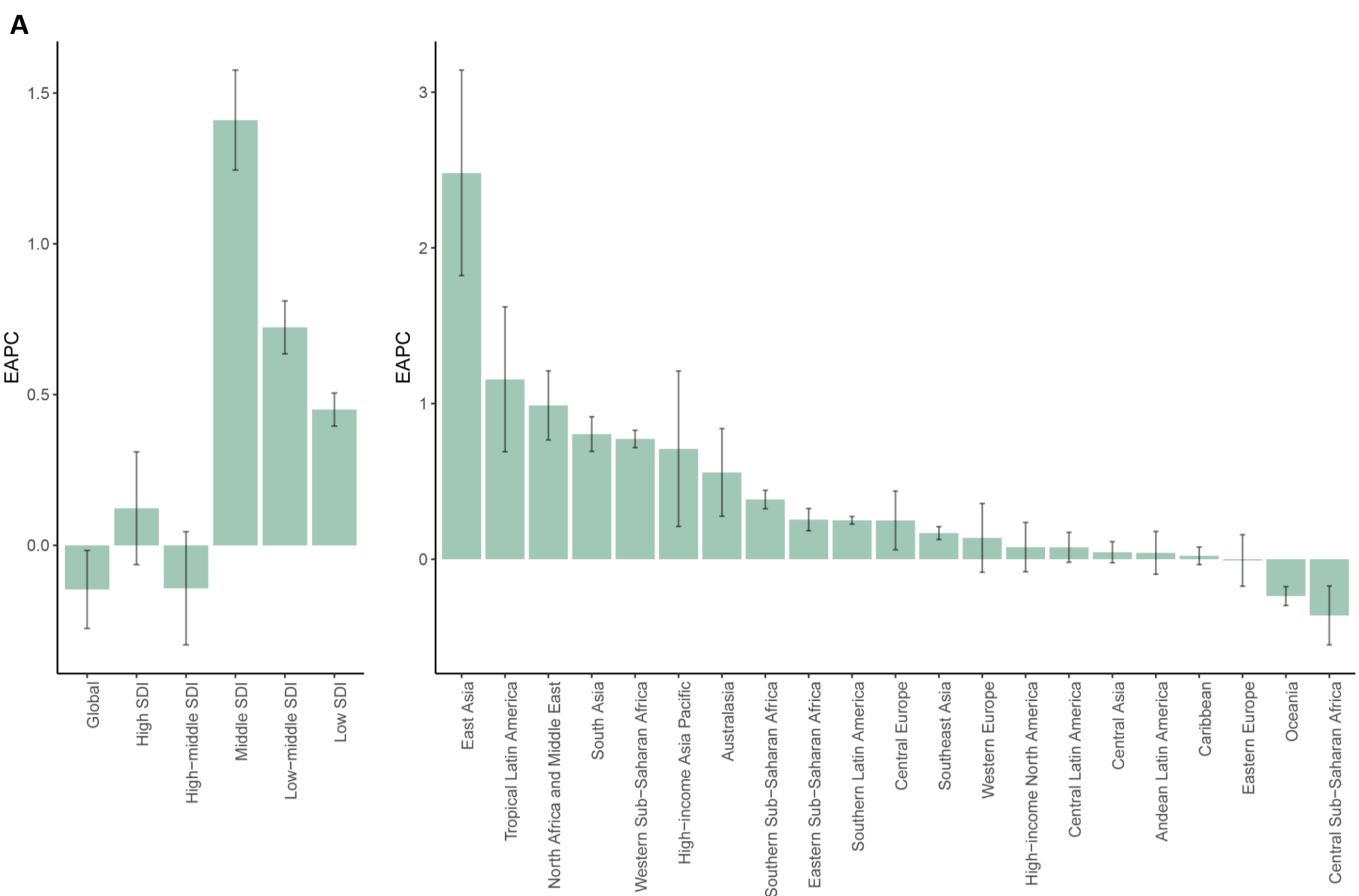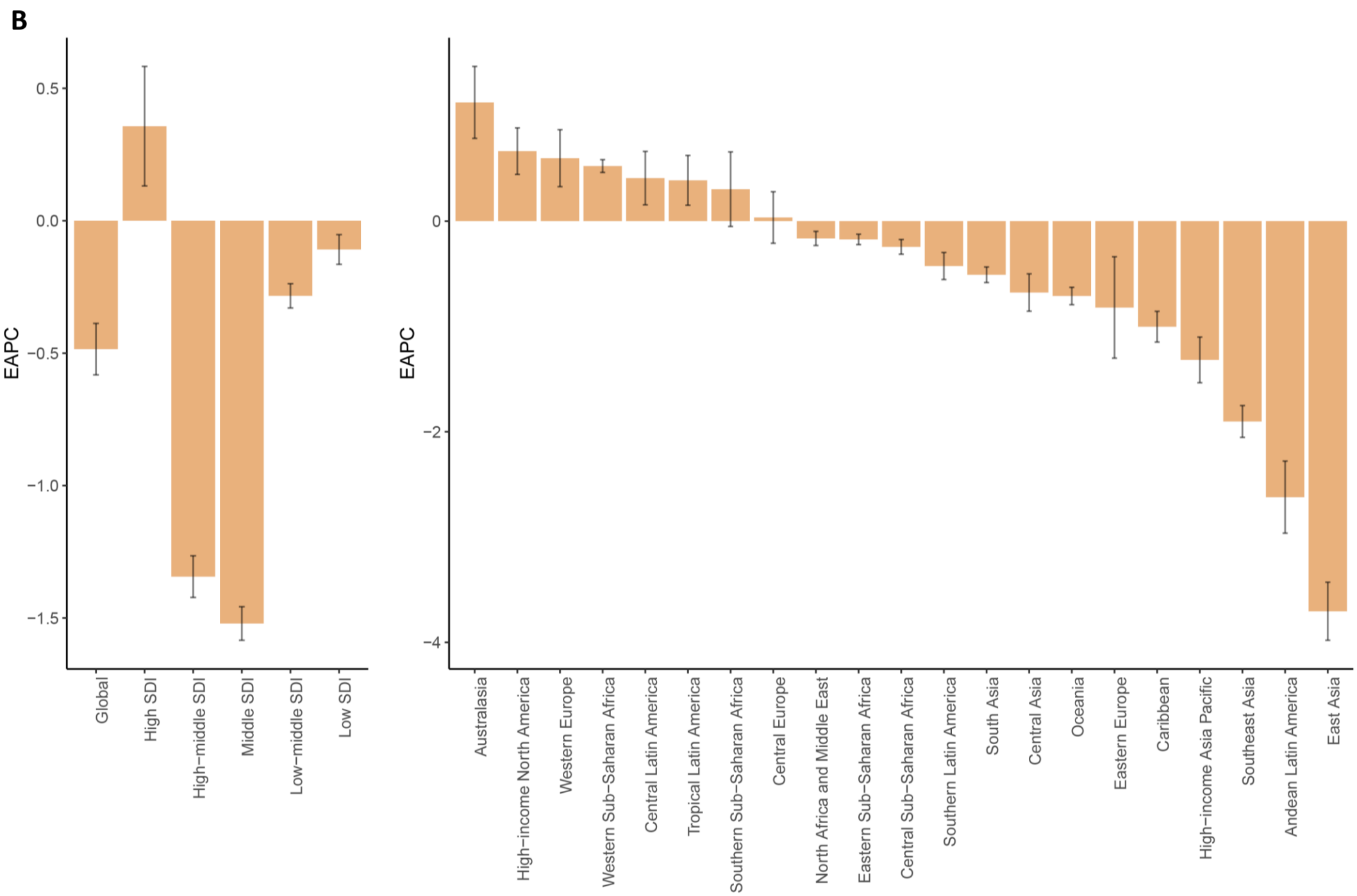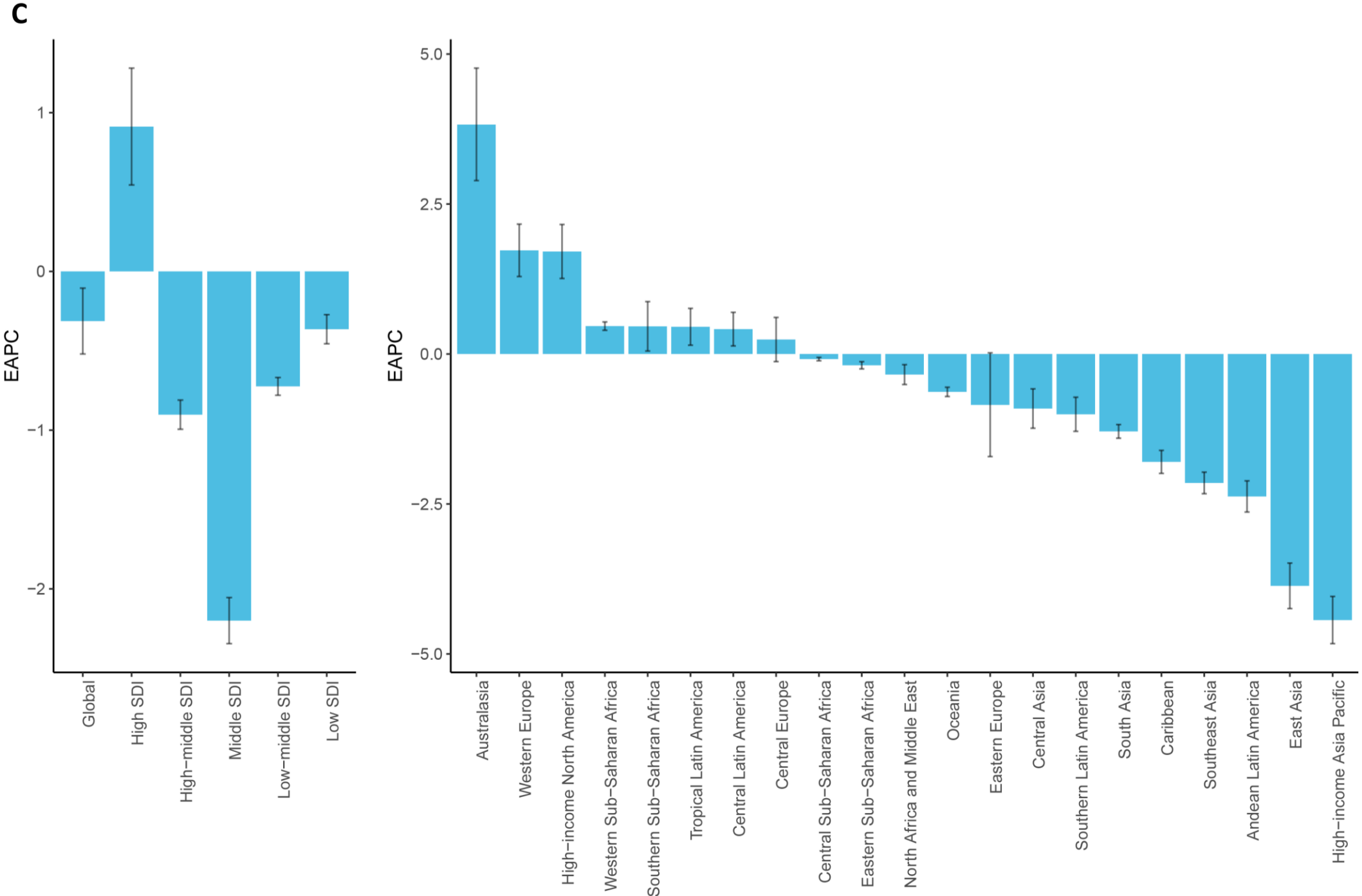

Supplement: S2 Fig — Abbreviation: IBD, inflammatory bowel disease; DALYs, disability-adjusted life-years; SDI, Socio-demographic Index; GBD, Global Burden of Disease; EAPC, estimated annual percentage change. (PDF) [file pone.0331034.s002.pdf]

A

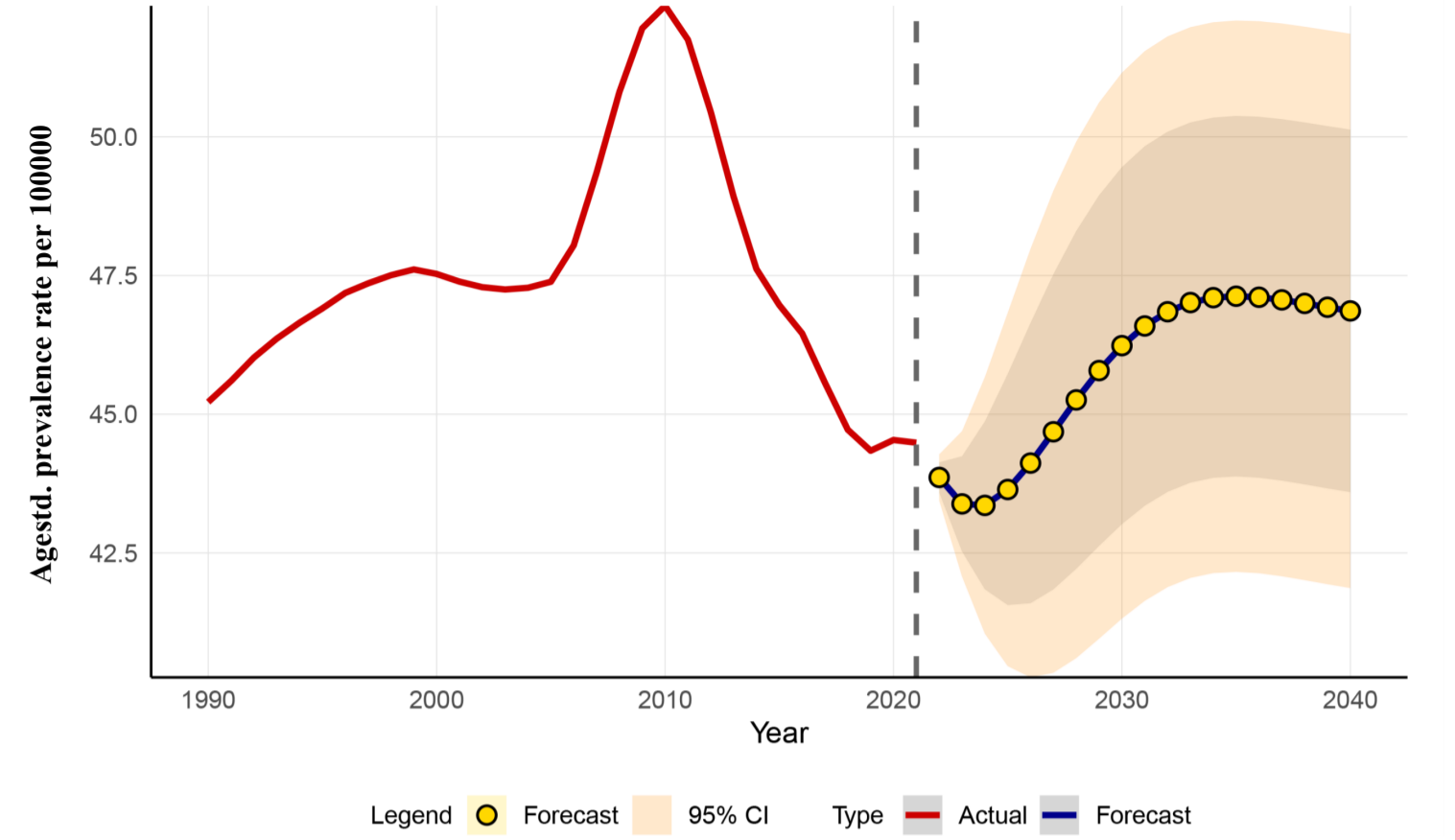

B

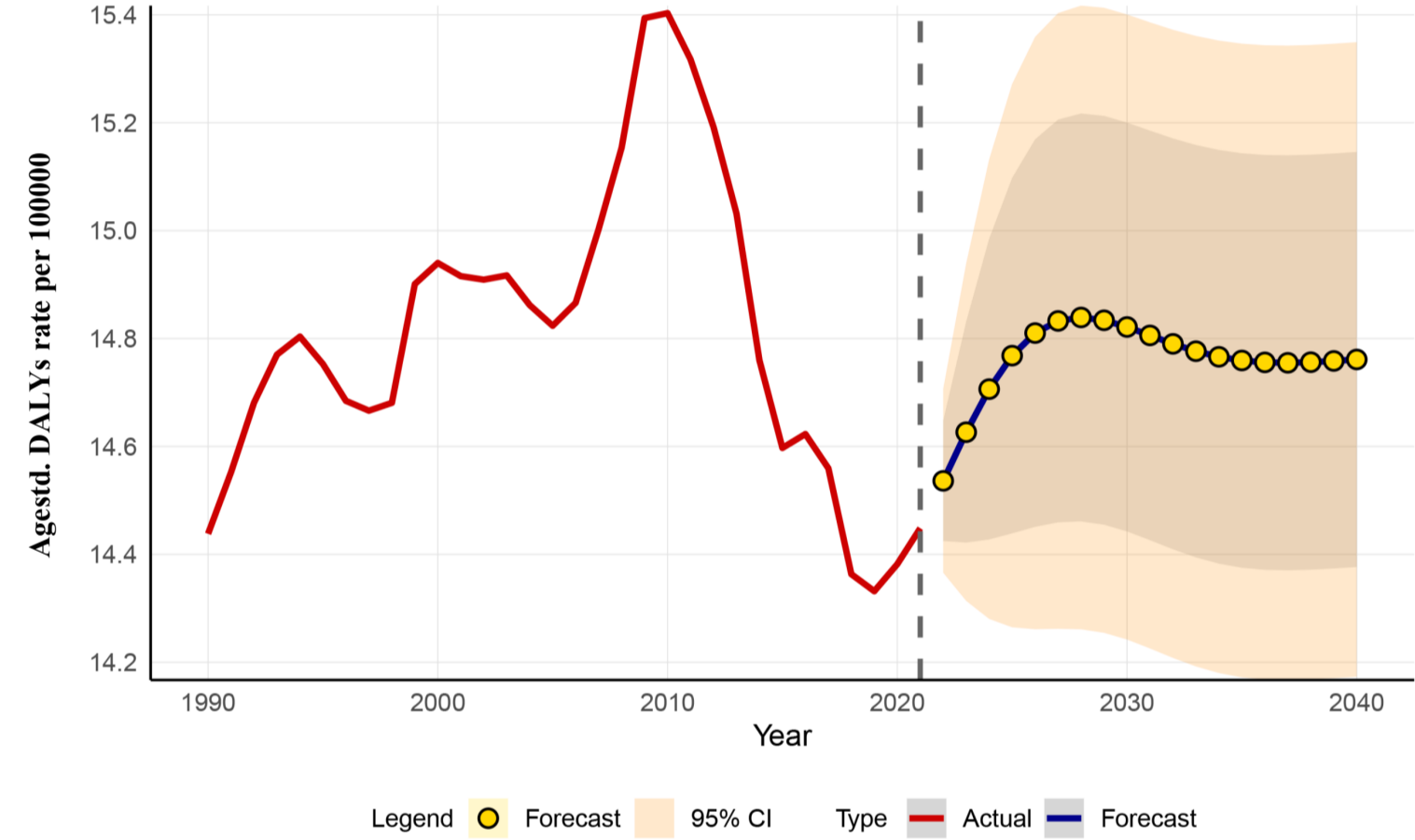

C

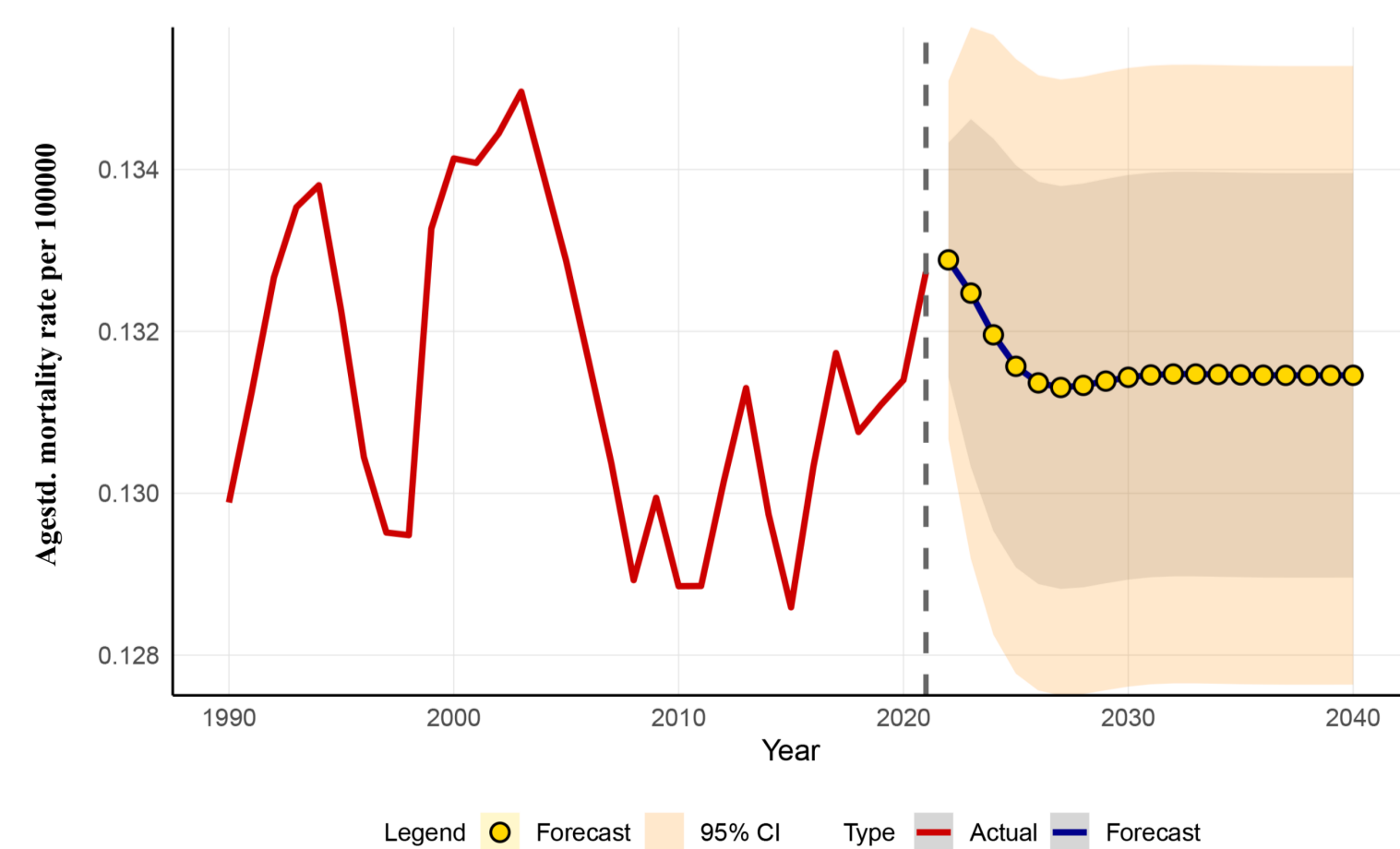

Supplement: S3 Fig — Abbreviation: DALYs, disability-adjusted life years; IBD, inflammatory bowel disease; WCBA, women of childbearing age. (PDF) [file pone.0331034.s003.pdf]
