# Supplementary material for: Global disease burden of inflammatory bowel disease in women and women of childbearing age from 1990 to 2021 and its prediction to 2040
Source: PLoS One. 2025 Sep 10;20(9):e0331034. doi: 10.1371/journal.pone.0331034 (PMC12422439; doi:10.1371/journal.pone.0331034)
Supplement: S1 Table — Abbreviations: IBD, inflammatory bowel disease; WCBA, women of childbearing age; SDI, Socio-demographic Index; DALYs, disability-adjusted life-years; EAPC, estimated annual percentage change. (DOCX) [file pone.0331034.s004.docx]

| **Table S1** The changes of prevalence, DALYs and mortality rate of IBD among WCBA from 1990 to 2021 | | | | | | | | | |
| --- | --- | --- | --- | --- | --- | --- | --- | --- | --- |
| Location | Prevalence rate of WCBA | | | DALYs rate of WCBA | | | Mortality rate of WCBA | | |
|  | 1990-per 100000  (95% UI) | 2021-per 100000  (95% UI) | EACP (95% CI) | 1990-per 100000  (95% UI) | 2021-per 100000  (95% UI) | EACP (95% CI) | 1990-per 100000  (95% UI) | 2021-per 100000  (95% UI) | EACP (95% CI) |
| **Global** | 45.22(37.98 to 53.75) | 44.49(36.13 to 54.05) | 0.01(-0.17 to 0.18) | 14.44(10.91 to 18.34) | 14.45(11.49 to 17.96) | -0.01(-0.09 to 0.06) | 0.13(0.08 to 0.17) | 0.13(0.10 to 0.16) | -0.05(-0.10 to 0.01) |
| **SDI regions** |  |  |  |  |  |  |  |  |  |
| High SDI | 159.26(136.03 to 187.79) | 160.06(130.29 to 195.48) | -0.08(-0.35 to 0.19) | 31.50(22.95 to 41.58) | 31.20(22.04 to 42.20) | -0.03(-0.28 to 0.23) | 0.13(0.12 to 0.13) | 0.13(0.13 to 0.14) | 0.48(0.17 to 0.79) |
| High middle SDI | 34.74(28.86 to 41.87) | 40.09(32.55 to 49.52) | 0.67(0.43 to 0.92) | 10.92(8.77 to 13.59) | 10.18(7.79 to 13.14) | -0.32(-0.45 to -0.20) | 0.10(0.08 to 0.12) | 0.07(0.06 to 0.09) | -1.50(-1.70 to -1.29) |
| Middle SDI | 13.43(10.97 to 16.62) | 23.20(18.71 to 28.76) | 2.09(1.88 to 2.31) | 7.73(5.35 to 9.86) | 8.31(6.55 to 10.52) | 0.35(0.27 to 0.43) | 0.10(0.06 to 0.13) | 0.08(0.06 to 0.10) | -0.69(-0.81 to -0.56) |
| Low middle SDI | 24.68(20.27 to 29.95) | 31.46(25.57 to 38.87) | 1.02(0.91 to 1.14) | 13.66(8.62 to 19.27) | 14.13(10.91 to 18.19) | 0.10(0.04 to 0.16) | 0.17(0.08 to 0.26) | 0.16(0.11 to 0.21) | -0.31(-0.38 to -0.25) |
| Low SDI | 17.15(13.93 to 21.15) | 18.92(15.26 to 23.51) | 0.44(0.35 to 0.52) | 17.28(9.81 to 25.96) | 18.77(12.01 to 23.97) | 0.24(0.17 to 0.30) | 0.25(0.12 to 0.40) | 0.27(0.16 to 0.36) | 0.17(0.09 to 0.24) |
| **Regions** |  |  |  |  |  |  |  |  |  |
| Andean Latin America | 13.27(10.64 to 16.71) | 15.20(12.18 to 19.02) | 0.36(0.20 to 0.53) | 7.13(5.13 to 9.41) | 4.68(3.52 to 6.20) | -1.62(-1.89 to -1.35) | 0.08(0.05 to 0.12) | 0.04(0.03 to 0.06) | -2.74(-3.02 to -2.45) |
| Australasia | 227.97(183.45 to 286.64) | 253.15(205.05 to 314.74) | 0.94(0.45 to 1.44) | 37.16(24.00 to 53.53) | 42.90(28.80 to 59.93) | 1.08(0.60 to 1.56) | 0.05(0.05 to 0.05) | 0.10(0.09 to 0.11) | 2.87(1.96 to 3.79) |
| Caribbean | 30.57(24.82 to 37.56) | 34.37(27.79 to 42.18) | 0.35(0.32 to 0.39) | 16.65(12.70 to 21.43) | 15.80(11.06 to 22.26) | -0.29(-0.42 to -0.16) | 0.21(0.15 to 0.29) | 0.19(0.11 to 0.31) | -0.57(-0.76 to -0.38) |
| Central Asia | 41.98(33.93 to 52.22) | 52.36(42.17 to 65.82) | 0.64(0.58 to 0.70) | 15.08(12.17 to 18.66) | 16.17(12.78 to 20.12) | -0.14(-0.34 to 0.06) | 0.14(0.12 to 0.16) | 0.14(0.11 to 0.16) | -0.76(-1.14 to -0.38) |
| Central Europe | 82.51(69.08 to 98.57) | 95.95(78.59 to 116.45) | 0.74(0.52 to 0.97) | 20.50(15.82 to 26.19) | 20.98(15.50 to 27.50) | 0.31(0.10 to 0.52) | 0.14(0.13 to 0.16) | 0.11(0.10 to 0.13) | -0.56(-0.81 to -0.30) |
| Central Latin America | 5.37(4.23 to 6.78) | 5.93(4.70 to 7.52) | 0.37(0.24 to 0.51) | 5.73(5.31 to 6.22) | 6.55(5.67 to 7.53) | 0.94(0.71 to 1.17) | 0.09(0.08 to 0.09) | 0.11(0.09 to 0.12) | 1.21(0.93 to 1.50) |
| Central Sub-Saharan Africa | 11.92(9.68 to 14.71) | 12.21(9.82 to 15.40) | -0.11(-0.27 to 0.04) | 7.42(4.24 to 11.65) | 7.19(4.77 to 10.24) | -0.15(-0.28 to -0.02) | 0.09(0.04 to 0.16) | 0.09(0.05 to 0.14) | -0.13(-0.26 to -0.00) |
| East Asia | 6.59(5.31 to 8.20) | 12.01(9.59 to 15.24) | 2.97(2.20 to 3.73) | 5.47(3.06 to 7.90) | 3.75(2.66 to 5.10) | -0.84(-1.15 to -0.53) | 0.08(0.04 to 0.12) | 0.04(0.02 to 0.06) | -2.95(-3.16 to -2.73) |
| Eastern Europe | 38.44(30.92 to 47.90) | 46.24(36.96 to 58.03) | 0.49(0.37 to 0.62) | 15.86(13.00 to 19.28) | 16.98(13.99 to 20.41) | -0.47(-0.83 to -0.12) | 0.19(0.17 to 0.22) | 0.19(0.17 to 0.22) | -0.97(-1.48 to -0.46) |
| Eastern Sub-Saharan Africa | 9.45(7.66 to 11.77) | 11.08(8.98 to 13.79) | 0.39(0.31 to 0.47) | 6.79(3.84 to 9.89) | 6.93(4.87 to 9.16) | -0.05(-0.11 to 0.02) | 0.09(0.04 to 0.15) | 0.09(0.05 to 0.13) | -0.20(-0.27 to -0.14) |
| High-income Asia Pacific | 34.05(28.23 to 42.03) | 43.86(35.32 to 54.80) | 1.15(0.48 to 1.82) | 42.09(29.64 to 56.27) | 42.38(30.42 to 57.20) | -0.33(-0.74 to 0.09) | 0.08(0.05 to 0.11) | 0.03(0.02 to 0.04) | -3.65(-3.95 to -3.35) |
| High-income North America | 232.71(197.29 to 275.82) | 212.92(173.83 to 261.38) | -0.38(-0.64 to -0.13) | 10.92(8.77 to 13.59) | 10.18(7.79 to 13.14) | 0.03(-0.22 to 0.27) | 0.12(0.11 to 0.12) | 0.20(0.19 to 0.21) | 1.96(1.57 to 2.35) |
| North Africa and Middle East | 30.59(25.04 to 37.98) | 41.78(33.81 to 51.48) | 1.30(1.16 to 1.43) | 10.50(7.55 to 15.23) | 10.66(7.80 to 14.43) | 0.18(0.11 to 0.25) | 0.09(0.05 to 0.16) | 0.07(0.05 to 0.11) | -1.08(-1.23 to -0.93) |
| Oceania | 6.45(5.13 to 8.26) | 6.42(5.08 to 8.05) | -0.14(-0.21 to -0.06) | 2.09(1.51 to 2.85) | 1.99(1.40 to 2.73) | -0.31(-0.41 to -0.22) | 0.02(0.01 to 0.03) | 0.02(0.01 to 0.03) | -0.38(-0.53 to -0.23) |
| South Asia | 32.99(26.93 to 40.47) | 43.38(35.17 to 53.69) | 1.17(1.00 to 1.34) | 14.86(8.93 to 22.04) | 13.63(9.84 to 18.64) | -0.28(-0.39 to -0.17) | 0.16(0.07 to 0.28) | 0.11(0.07 to 0.19) | -1.45(-1.59 to -1.30) |
| Southeast Asia | 6.40(5.21 to 7.99) | 7.33(5.87 to 9.29) | 0.56(0.51 to 0.61) | 4.22(2.10 to 6.00) | 3.21(2.29 to 4.11) | -1.07(-1.17 to -0.96) | 0.06(0.02 to 0.09) | 0.04(0.02 to 0.05) | -1.73(-1.90 to -1.57) |
| Southern Latin America | 55.57(44.35 to 70.43) | 63.83(51.01 to 82.37) | 0.38(0.34 to 0.42) | 14.51(10.74 to 18.93) | 13.72(9.59 to 18.80) | -0.18(-0.30 to -0.06) | 0.10(0.09 to 0.11) | 0.07(0.06 to 0.07) | -1.28(-1.62 to -0.93) |
| Southern Sub-Saharan Africa | 11.76(9.45 to 14.72) | 15.13(12.28 to 18.97) | 0.62(0.55 to 0.70) | 8.40(5.75 to 10.84) | 7.93(5.50 to 11.06) | 0.14(-0.48 to 0.77) | 0.11(0.07 to 0.15) | 0.10(0.05 to 0.16) | 0.25(-0.58 to 1.09) |
| Tropical Latin America | 13.96(11.25 to 17.33) | 23.52(18.71 to 29.73) | 1.33(0.79 to 1.87) | 12.49(11.59 to 13.76) | 15.81(14.08 to 17.92) | 0.73(0.49 to 0.96) | 0.18(0.18 to 0.19) | 0.23(0.21 to 0.24) | 0.64(0.35 to 0.94) |
| Western Europe | 190.44(162.39 to 220.86) | 202.24(165.38 to 246.16) | 0.05(-0.25 to 0.36) | 37.27(27.15 to 49.27) | 38.13(26.77 to 51.95) | 0.08(-0.24 to 0.39) | 0.15(0.14 to 0.15) | 0.15(0.14 to 0.15) | 0.55(0.07 to 1.03) |
| Western Sub-Saharan Africa | 10.58(8.56 to 13.11) | 12.78(10.47 to 15.68) | 0.62(0.59 to 0.65) | 36.78(21.09 to 51.55) | 44.49(23.02 to 64.18) | 0.67(0.59 to 0.74) | 0.61(0.34 to 0.87) | 0.73(0.37 to 1.07) | 0.64(0.56 to 0.72) |
| **Abbreviations:** IBD, inflammatory bowel disease; WCBA, women of childbearing age; SDI, Socio-demographic Index; DALYs, disability-adjusted life-years; EAPC, estimated annual percentage change. | | | | | | | | | |
