# Supplementary material for: Global disease burden of inflammatory bowel disease in women and women of childbearing age from 1990 to 2021 and its prediction to 2040
Source: PLoS One. 2025 Sep 10;20(9):e0331034. doi: 10.1371/journal.pone.0331034 (PMC12422439; doi:10.1371/journal.pone.0331034)
Supplement: S2 Table — Abbreviations: IBD, inflammatory bowel disease; AS, age-standardized; SDI, Socio-demographic Index; DALYs, disability-adjusted life-years; EAPC, estimated annual percentage change. (DOCX) [file pone.0331034.s005.docx]

| **Table S2** The changes of AS prevalence, DALYs and mortality rate of IBD among women from 1990 to 2021 | | | | | | | | | |
| --- | --- | --- | --- | --- | --- | --- | --- | --- | --- |
|  | AS prevalence rate | | | AS DALYs rate | | | AS mortality rate | | |
| Location | 1990-per 100 000  (95% UI) | 2021-per 100 000  (95% UI) | EACP  (95% CI) | 1990-per 100 000  (95% UI) | 2021-per 100 000  (95% UI) | EACP  (95% CI) | 1990-per 100 000  (95% UI) | 2021-per 100 000  (95% UI) | EACP  (95% CI) |
| **Global** | 49.43(43.20 to 57.62) | 45.90(39.71 to 53.97) | -0.15(-0.28 to -0.02) | 21.18(16.65 to 25.88) | 17.75(15.08 to 21.23) | -0.48(-0.58 to -0.39) | 0.58(0.46 to 0.68) | 0.50(0.42 to 0.59) | -0.31(-0.52 to -0.11) |
| **SDI regions** |  |  |  |  |  |  |  |  |  |
| High SDI | 132.99(117.02 to 152.72) | 138.08(119.37 to 161.05) | 0.12(-0.06 to 0.31) | 34.04(27.89 to 41.70) | 35.75(28.88 to 43.78) | 0.36(0.13 to 0.58) | 0.70(0.64 to 0.75) | 0.81(0.69 to 0.88) | 0.91(0.54 to 1.28) |
| High middle SDI | 36.08(31.24 to 42.66) | 32.55(27.92 to 39.24) | -0.14(-0.33 to 0.05) | 17.96(14.59 to 21.55) | 12.23(10.08 to 14.81) | -1.34(-1.42 to -1.27) | 0.47(0.39 to 0.54) | 0.36(0.30 to 0.47) | -0.90(-0.99 to -0.81) |
| Middle SDI | 14.31(12.17 to 17.25) | 20.23(17.15 to 24.59) | 1.41(1.24 to 1.58) | 15.54(9.03 to 20.38) | 9.86(7.98 to 11.96) | -1.52(-1.58 to -1.46) | 0.51(0.27 to 0.68) | 0.28(0.20 to 0.37) | -2.20(-2.34 to -2.05) |
| Low middle SDI | 28.16(24.02 to 33.73) | 33.03(28.11 to 39.95) | 0.72(0.64 to 0.81) | 16.56(10.96 to 22.96) | 14.96(11.86 to 19.46) | -0.28(-0.33 to -0.24) | 0.42(0.24 to 0.62) | 0.33(0.26 to 0.48) | -0.72(-0.78 to -0.67) |
| Low SDI | 20.69(17.44 to 24.89) | 23.06(19.52 to 27.89) | 0.45(0.40 to 0.51) | 17.16(10.47 to 25.82) | 16.63(12.04 to 20.77) | -0.11(-0.16 to -0.05) | 0.40(0.23 to 0.59) | 0.36(0.25 to 0.49) | -0.36(-0.46 to -0.27) |
| **Regions** |  |  |  |  |  |  |  |  |  |
| Andean Latin America | 13.51(11.38 to 16.33) | 13.87(11.72 to 16.55) | 0.04(-0.10 to 0.18) | 12.33(7.77 to 18.26) | 5.81(4.44 to 7.65) | -2.62(-2.96 to -2.28) | 0.27(0.18 to 0.37) | 0.13(0.10 to 0.19) | -2.37(-2.63 to -2.11) |
| Australasia | 202.91(170.93 to 244.86) | 214.00(182.06 to 255.17) | 0.56(0.28 to 0.84) | 36.14(25.79 to 48.35) | 44.35(33.36 to 57.46) | 1.13(0.79 to 1.47) | 0.32(0.29 to 0.35) | 0.79(0.64 to 0.91) | 3.82(2.89 to 4.77) |
| Caribbean | 29.82(24.96 to 36.05) | 29.60(24.89 to 35.43) | 0.02(-0.03 to 0.08) | 20.73(16.30 to 26.21) | 15.52(11.29 to 21.46) | -1.00(-1.15 to -0.86) | 0.55(0.45 to 0.69) | 0.33(0.24 to 0.46) | -1.80(-1.99 to -1.61) |
| Central Asia | 46.98(39.75 to 56.32) | 46.28(39.26 to 55.44) | 0.04(-0.02 to 0.11) | 18.73(15.35 to 22.63) | 16.46(13.52 to 19.82) | -0.68(-0.86 to -0.50) | 0.28(0.25 to 0.31) | 0.25(0.22 to 0.29) | -0.91(-1.24 to -0.58) |
| Central Europe | 66.22(57.09 to 77.82) | 68.01(58.09 to 80.07) | 0.25(0.06 to 0.44) | 20.90(17.33 to 25.10) | 19.51(15.89 to 23.82) | 0.03(-0.21 to 0.28) | 0.39(0.36 to 0.44) | 0.38(0.34 to 0.42) | 0.24(-0.13 to 0.61) |
| Central Latin America | 5.52(4.64 to 6.70) | 5.51(4.58 to 6.69) | 0.08(-0.02 to 0.17) | 9.73(9.24 to 10.29) | 9.20(8.18 to 10.41) | 0.41(0.15 to 0.66) | 0.35(0.33 to 0.37) | 0.33(0.29 to 0.37) | 0.41(0.13 to 0.69) |
| Central Sub-Saharan Africa | 13.88(11.77 to 16.69) | 13.09(11.07 to 15.69) | -0.36(-0.55 to -0.17) | 9.55(5.47 to 13.85) | 8.81(6.06 to 12.43) | -0.24(-0.31 to -0.17) | 0.25(0.13 to 0.40) | 0.24(0.14 to 0.40) | -0.08(-0.11 to -0.06) |
| East Asia | 5.92(5.01 to 7.05) | 9.36(7.96 to 11.26) | 2.48(1.82 to 3.14) | 19.13(9.32 to 27.40) | 6.33(4.54 to 9.52) | -3.71(-3.98 to -3.43) | 0.75(0.38 to 1.02) | 0.26(0.17 to 0.44) | -3.87(-4.24 to -3.49) |
| Eastern Europe | 38.46(32.58 to 46.58) | 37.27(31.64 to 44.70) | -0.01(-0.17 to 0.16) | 19.86(17.15 to 22.82) | 17.87(15.52 to 20.48) | -0.82(-1.30 to -0.34) | 0.50(0.46 to 0.55) | 0.47(0.42 to 0.52) | -0.85(-1.71 to 0.02) |
| Eastern Sub-Saharan Africa | 11.54(9.73 to 13.90) | 13.15(11.20 to 15.60) | 0.25(0.18 to 0.33) | 10.62(6.90 to 15.09) | 10.17(7.13 to 13.48) | -0.17(-0.22 to -0.12) | 0.32(0.20 to 0.47) | 0.30(0.19 to 0.46) | -0.19(-0.25 to -0.13) |
| High-income Asia Pacific | 29.89(25.61 to 35.61) | 35.56(29.99 to 42.96) | 0.71(0.21 to 1.21) | 11.60(8.81 to 14.18) | 7.27(5.25 to 9.58) | -1.32(-1.53 to -1.10) | 0.33(0.23 to 0.41) | 0.09(0.06 to 0.14) | -4.44(-4.83 to -4.04) |
| High-income North America | 203.12(179.88 to 235.07) | 202.93(175.82 to 237.34) | 0.08(-0.08 to 0.24) | 44.27(34.64 to 55.91) | 51.06(41.65 to 62.53) | 0.66(0.44 to 0.89) | 0.68(0.60 to 0.71) | 1.02(0.89 to 1.10) | 1.71(1.26 to 2.16) |
| North Africa and Middle East | 30.04(25.49 to 36.06) | 36.76(30.88 to 44.19) | 0.99(0.77 to 1.21) | 13.34(9.85 to 19.03) | 12.08(9.36 to 16.21) | -0.16(-0.23 to -0.10) | 0.31(0.21 to 0.48) | 0.26(0.19 to 0.40) | -0.34(-0.51 to -0.18) |
| Oceania | 6.07(5.05 to 7.45) | 5.75(4.78 to 7.04) | -0.24(-0.30 to -0.18) | 2.66(1.85 to 3.48) | 2.20(1.59 to 2.88) | -0.71(-0.79 to -0.63) | 0.07(0.04 to 0.09) | 0.05(0.03 to 0.08) | -0.63(-0.71 to -0.55) |
| South Asia | 39.66(33.68 to 47.65) | 47.59(40.43 to 57.28) | 0.80(0.69 to 0.92) | 18.86(12.27 to 26.92) | 15.99(11.97 to 22.05) | -0.51(-0.58 to -0.44) | 0.46(0.24 to 0.72) | 0.31(0.22 to 0.50) | -1.29(-1.40 to -1.18) |
| Southeast Asia | 5.82(4.90 to 6.92) | 5.96(5.02 to 7.20) | 0.17(0.13 to 0.21) | 8.24(3.73 to 12.31) | 5.02(3.33 to 6.41) | -1.90(-2.05 to -1.75) | 0.32(0.12 to 0.52) | 0.18(0.11 to 0.25) | -2.15(-2.33 to -1.97) |
| Southern Latin America | 51.02(42.59 to 62.08) | 55.05(46.24 to 68.27) | 0.25(0.23 to 0.27) | 15.96(13.00 to 19.49) | 13.72(10.75 to 17.53) | -0.43(-0.55 to -0.30) | 0.33(0.30 to 0.36) | 0.23(0.21 to 0.25) | -1.00(-1.29 to -0.72) |
| Southern Sub-Saharan Africa | 14.94(12.67 to 17.92) | 17.62(15.03 to 21.15) | 0.38(0.32 to 0.44) | 11.66(8.26 to 14.76) | 11.61(8.47 to 15.32) | 0.30(-0.05 to 0.66) | 0.32(0.19 to 0.43) | 0.33(0.21 to 0.46) | 0.46(0.05 to 0.87) |
| Tropical Latin America | 13.29(11.31 to 15.77) | 21.25(17.91 to 25.58) | 1.15(0.69 to 1.62) | 15.34(14.46 to 16.51) | 17.02(15.43 to 18.70) | 0.39(0.15 to 0.62) | 0.46(0.43 to 0.49) | 0.50(0.45 to 0.54) | 0.45(0.15 to 0.76) |
| Western Europe | 147.54(128.46 to 167.65) | 160.45(137.27 to 188.08) | 0.14(-0.08 to 0.36) | 37.83(30.67 to 46.20) | 42.72(34.53 to 52.40) | 0.60(0.33 to 0.87) | 0.79(0.71 to 0.84) | 1.10(0.92 to 1.21) | 1.73(1.29 to 2.16) |
| Western Sub-Saharan Africa | 13.05(11.05 to 15.67) | 16.45(14.02 to 19.90) | 0.77(0.72 to 0.83) | 23.35(13.74 to 32.31) | 26.96(14.86 to 37.59) | 0.52(0.46 to 0.58) | 0.39(0.22 to 0.56) | 0.45(0.23 to 0.64) | 0.47(0.40 to 0.53) |
| **Abbreviations:** IBD, inflammatory bowel disease; AS, age-standardized; SDI, Socio-demographic Index; DALYs, disability-adjusted life-years; EAPC, estimated annual percentage change. | | | | | | | | | |
