# Supplementary material for: Global disease burden of inflammatory bowel disease in women and women of childbearing age from 1990 to 2021 and its prediction to 2040
Source: PLoS One. 2025 Sep 10;20(9):e0331034. doi: 10.1371/journal.pone.0331034 (PMC12422439; doi:10.1371/journal.pone.0331034)
Supplement: S3 Table — Abbreviations: DALYs, disability-adjusted life years; IBD, inflammatory bowel disease; SDI, Socio-demographic Index. (DOCX) [file pone.0331034.s006.docx]

| **Table S3 Decomposition analysis of the changes in prevalence, DALYs and** **mortality rates for IBD in women** | | | | | | |  |  |  |
| --- | --- | --- | --- | --- | --- | --- | --- | --- | --- |
| Location | Prevalence | | | DALYs | | | Mortality | | |
|  | Aging (Percentage) | Population (Percentage) | Epidemiological change (Percentage) | Aging (Percentage) | Population (Percentage) | Epidemiological change (Percentage) | Aging (Percentage) | Population (Percentage) | Epidemiological change (Percentage) |
| **Global** | 88453.25(33.73％) | 274971.91(104.85％) | -101182.98(-38.58％) | 20023.72(22.63％) | 88643.55(100.17％) | -20177.59(-22.8％) | 174.48(20.53％) | 807.43(95.02％) | -132.2(-15.56％) |
| **SDI regions** |  |  |  |  |  |  |  |  |  |
| High SDI | -90753.11(-322.33％) | 143032.37(508.01％) | -24123.75(-85.68％) | -18017.58(-404.73％) | 28092.48(631.04％) | -5623.11(-126.31％) | -68.36(-166.86％) | 116.45(284.24％) | -7.12(-17.37％) |
| High-middle SDI | -10815.5(-41.88％) | 41349.81(160.11％) | -4707.75(-18.23％) | -3659.37(-496.27％) | 11777.89(1597.27％) | -7381.14(-1001％) | -21.79(42.8％) | 98.43(-193.32％) | -127.56(250.52％) |
| Middle SDI | 13416.07(16.07％) | 35912.24(43.02％) | 34158.8(40.92％) | 3742.53(22.22％) | 16016.79(95.08％) | -2914.03(-17.3％) | 52.22(78.58％) | 181.88(273.71％) | -167.65(-252.29％) |
| Low-middle SDI | 35938.29(39.09％) | 40063.27(43.58％) | 15929.37(17.33％) | 15894.67(46.39％) | 19778.89(57.72％) | -1408.36(-4.11％) | 183.78(54.98％) | 228.22(68.28％) | -77.74(-23.26％) |
| Low SDI | 17421.63(53.18％) | 12432.78(37.95％) | 2906.65(8.87％) | 17225.32(53.53％) | 12414.2(38.58％) | 2541.14(7.9％) | 250.56(54.59％) | 179.65(39.14％) | 28.78(6.27％) |
| **Regions** |  |  |  |  |  |  |  |  |  |
| Andean Latin America | 687.4(49.34％) | 704.56(50.57％) | 1.22(0.09％) | 243.09(173.49％) | 289.49(206.6％) | -392.46(-280.09％) | 2.53(-239.12％) | 2.99(-282.93％) | -6.58(622.05％) |
| Australasia | -145.98(-2.42％) | 5662.98(93.77％) | 522.15(8.65％) | -28.07(-2.55％) | 941.52(85.41％) | 188.88(17.13％) | 0.03(0.63％) | 1.71(39.41％) | 2.61(59.96％) |
| Caribbean | -24.31(-1.89％) | 1301.1(101.25％) | 8.21(0.64％) | -48.12(-13.79％) | 653.75(187.38％) | -256.74(-73.59％) | -0.16(-5.66％) | 8(279.43％) | -4.97(-173.77％) |
| Central Asia | 1744.12(30.81％) | 3606.27(63.7％) | 311.31(5.5％) | 357.17(25.62％) | 1194.37(85.69％) | -157.66(-11.31％) | 2.63(27.1％) | 10.44(107.42％) | -3.36(-34.52％) |
| Central Europe | -11300.29(1792.61％) | 9711.95(-1540.65％) | 957.96(-151.97％) | -2675.4(300.55％) | 2273.86(-255.44％) | -488.63(54.89％) | -15.38(116.44％) | 13.93(-105.41％) | -11.76(88.97％) |
| Central Latin America | 788.48(43.94％) | 1152.07(64.2％) | -146(-8.14％) | 791.14(38.38％) | 1250.67(60.67％) | 19.76(0.96％) | 14.6(41.68％) | 19.72(56.28％) | 0.71(2.03％) |
| Central Sub-Saharan Africa | 1549.28(61.65％) | 962.76(38.31％) | 0.82(0.03％) | 922.44(64.48％) | 580.83(40.6％) | -72.74(-5.08％) | 11.41(66.01％) | 7.15(41.34％) | -1.27(-7.35％) |
| East Asia | -6254.83(-35.2％) | 11367.03(63.97％) | 12656.63(71.23％) | -2980.54(51.1％) | 6027.87(-103.35％) | -8880.02(152.25％) | -23.3(15.22％) | 78.48(-51.29％) | -208.2(136.06％) |
| Eastern Europe | -8049.58(-763.84％) | 8388.04(795.95％) | 715.38(67.88％) | -3171.41(547.31％) | 3274.03(-565.03％) | -682.07(117.71％) | -33.08(339.11％) | 37.65(-385.96％) | -14.33(146.86％) |
| Eastern Sub-Saharan Africa | 4058.8(52.13％) | 2761.32(35.46％) | 966.28(12.41％) | 2673.13(59.52％) | 1835.54(40.87％) | -17.88(-0.4％) | 35.77(63.88％) | 24.31(43.41％) | -4.08(-7.29％) |
| High-income Asia Pacific | -7385.9(-665.69％) | 6228.02(561.33％) | 2267.39(204.36％) | -1875.6(120.06％) | 1534.26(-98.21％) | -1220.92(78.15％) | -11.49(40.43％) | 9.54(-33.58％) | -26.47(93.15％) |
| High-income North America | -41408.4(-709.07％) | 67238.58(1151.38％) | -19990.34(-342.31％) | -7838.84(-182.11％) | 12712.07(295.33％) | -568.86(-13.22％) | -26.8(-32.27％) | 47.66(57.38％) | 62.2(74.89％) |
| North Africa and Middle East | 20478.52(47.99％) | 15707.51(36.81％) | 6487.43(15.2％) | 5414(61.63％) | 4566.79(51.99％) | -1196.54(-13.62％) | 39.29(113.14％) | 34.12(98.25％) | -38.68(-111.38％) |
| Oceania | 75.11(61.28％) | 58.24(47.52％) | -10.78(-8.8％) | 25.74(70.53％) | 18.51(50.72％) | -7.75(-21.25％) | 0.25(83.29％) | 0.16(53.28％) | -0.11(-36.58％) |
| South Asia | 52856.93(40.58％) | 52268.61(40.13％) | 25123.61(19.29％) | 17977.58(61.06％) | 19375.26(65.81％) | -7911.82(-26.87％) | 167.45(125.15％) | 183.87(137.43％) | -217.52(-162.58％) |
| Southeast Asia | 1625.8(28.37％) | 3864.86(67.43％) | 240.69(4.2％) | 722.29(89.57％) | 2103.15(260.81％) | -2019.03(-250.38％) | 11.41(-1082.83％) | 25.87(-2455.63％) | -38.33(3638.46％) |
| Southern Latin America | 381.77(9.01％) | 3318.15(78.28％) | 538.86(12.71％) | 66.58(11.24％) | 786.31(132.77％) | -260.66(-44.01％) | 0.39(-41.11％) | 4.8(-502.37％) | -6.15(643.48％) |
| Southern Sub-Saharan Africa | 595.64(34.57％) | 867.81(50.36％) | 259.76(15.07％) | 323.13(53.43％) | 525.94(86.96％) | -244.24(-40.38％) | 4.56(64.78％) | 6.88(97.69％) | -4.4(-62.47％) |
| Tropical Latin America | 1794.21(20.66％) | 3454.2(39.78％) | 3435.13(39.56％) | 1219.78(26.52％) | 2632.91(57.25％) | 746.02(16.22％) | 21.18(33.71％) | 38.18(60.76％) | 3.47(5.53％) |
| Western Europe | -62471.83(-962.37％) | 70979.67(1093.43％) | -2016.36(-31.06％) | -12059.53(14365.49％) | 13653.29(-16264％) | -1677.7(1998.51％) | -42.42(1817.72％) | 52.77(-2261.37％) | -12.69(543.65％) |
| Western Sub-Saharan Africa | 5848.43(54.59％) | 3401.56(31.75％) | 1464.17(13.67％) | 19839.36(53.19％) | 11835.18(31.73％) | 5627.23(15.09％) | 331.97(54.2％) | 196.05(32.01％) | 84.49(13.79％) |
| **Abbreviations:** DALYs, disability-adjusted life years; IBD, inflammatory bowel disease; SDI, Socio-demographic Index. | | | | | | | | | |
