# Supplementary material for: Global disease burden of inflammatory bowel disease in women and women of childbearing age from 1990 to 2021 and its prediction to 2040
Source: PLoS One. 2025 Sep 10;20(9):e0331034. doi: 10.1371/journal.pone.0331034 (PMC12422439; doi:10.1371/journal.pone.0331034)
Supplement: S4 Table — Abbreviations: IBD, inflammatory bowel disease; AS, age-standardized; WCBA, women of childbearing age; EAPC, estimated annual percentage change; CI, Confidence Interval. (DOCX) [file pone.0331034.s007.docx]

Table S4 The prevalence rate for IBD among women and WCBA and its temporal trends from 1990 to 2021 across 204 countries and regions

| Location | Prevalence rate of WCBA_1990 | Prevalence rate of WCBA_2021 | EAPC_CI | AS prevalence rate of women_1990 | AS prevalence rate of women_2021 | EAPC_CI |
| --- | --- | --- | --- | --- | --- | --- |
| Afghanistan | 19.80(15.91 to 25.35) | 23.68(19.20 to 29.63) | 0.97(0.80 to 1.13) | 19.53(15.95 to 23.93) | 23.39(19.38 to 28.40) | 0.83(0.66 to 1.01) |
| Albania | 71.78(56.54 to 91.12) | 86.35(68.89 to 110.12) | 0.41(0.33 to 0.49) | 66.59(55.38 to 81.59) | 65.62(54.22 to 80.76) | -0.13(-0.24 to -0.02) |
| Algeria | 32.42(25.24 to 42.18) | 49.23(38.35 to 63.15) | 1.53(1.40 to 1.65) | 32.37(26.34 to 39.66) | 39.03(31.76 to 48.33) | 0.86(0.67 to 1.04) |
| American Samoa | 8.04(6.25 to 10.20) | 8.82(6.81 to 11.26) | 0.20(0.14 to 0.25) | 7.75(6.39 to 9.47) | 7.72(6.36 to 9.42) | -0.03(-0.08 to 0.02) |
| Andorra | 176.67(138.22 to 218.85) | 214.53(171.41 to 271.23) | 0.71(0.62 to 0.79) | 151.61(126.10 to 180.60) | 158.70(133.62 to 193.31) | 0.18(0.16 to 0.20) |
| Angola | 11.39(8.97 to 14.51) | 13.47(10.73 to 17.25) | 0.49(0.42 to 0.56) | 12.14(10.01 to 14.87) | 14.12(11.75 to 16.90) | 0.40(0.33 to 0.47) |
| Antigua and Barbuda | 35.99(28.81 to 44.81) | 45.14(35.21 to 57.46) | 0.69(0.62 to 0.76) | 34.29(28.68 to 41.49) | 35.79(29.54 to 44.19) | 0.14(0.12 to 0.17) |
| Argentina | 57.13(44.56 to 73.01) | 64.03(50.14 to 83.40) | 0.31(0.26 to 0.36) | 50.27(41.13 to 61.65) | 53.87(44.85 to 66.99) | 0.23(0.20 to 0.27) |
| Armenia | 44.83(36.53 to 54.79) | 62.78(50.14 to 77.76) | 0.85(0.75 to 0.95) | 48.58(40.45 to 58.22) | 51.85(43.07 to 62.76) | 0.29(0.27 to 0.32) |
| Australia | 231.36(186.29 to 290.94) | 260.94(210.28 to 324.00) | 1.07(0.53 to 1.63) | 200.91(168.82 to 244.64) | 216.79(183.53 to 259.53) | 0.68(0.36 to 1.01) |
| Austria | 149.19(121.39 to 182.23) | 174.15(141.63 to 214.11) | 0.09(-0.14 to 0.31) | 122.28(102.07 to 145.69) | 137.40(115.35 to 164.78) | 0.16(0.05 to 0.27) |
| Azerbaijan | 41.84(33.58 to 52.51) | 58.07(46.24 to 72.22) | 0.99(0.93 to 1.04) | 46.48(38.70 to 56.09) | 51.11(41.91 to 61.03) | 0.41(0.27 to 0.56) |
| Bahamas | 35.73(28.29 to 44.77) | 40.70(32.07 to 51.87) | 0.40(0.29 to 0.50) | 35.68(29.62 to 43.63) | 33.04(27.68 to 40.76) | -0.17(-0.23 to -0.11) |
| Bahrain | 36.85(30.18 to 45.40) | 45.30(36.45 to 56.22) | 1.00(0.85 to 1.16) | 32.86(27.58 to 39.13) | 36.24(30.28 to 44.05) | 0.67(0.54 to 0.81) |
| Bangladesh | 39.30(31.22 to 50.56) | 53.93(41.80 to 68.61) | 1.17(1.10 to 1.25) | 43.30(36.18 to 52.48) | 51.18(42.81 to 62.64) | 0.75(0.67 to 0.84) |
| Barbados | 42.59(35.53 to 51.68) | 50.25(39.30 to 64.41) | 0.42(0.17 to 0.68) | 39.19(33.62 to 45.66) | 40.22(33.22 to 50.27) | 0.10(-0.04 to 0.24) |
| Belarus | 45.92(36.71 to 57.35) | 61.03(48.79 to 77.24) | 0.83(0.74 to 0.92) | 44.64(37.09 to 55.01) | 46.18(38.64 to 56.15) | 0.20(0.03 to 0.37) |
| Belgium | 124.56(106.40 to 145.37) | 151.61(123.43 to 186.38) | 0.19(0.05 to 0.33) | 97.37(86.11 to 111.00) | 119.45(100.27 to 144.92) | 0.37(0.28 to 0.46) |
| Belize | 25.85(20.52 to 32.31) | 31.80(25.55 to 40.64) | 0.64(0.60 to 0.68) | 26.88(22.56 to 32.65) | 27.78(22.75 to 34.69) | 0.13(0.09 to 0.17) |
| Benin | 10.27(8.22 to 12.86) | 13.02(10.32 to 16.22) | 0.82(0.73 to 0.90) | 11.46(9.72 to 13.66) | 14.73(12.25 to 18.22) | 0.90(0.85 to 0.95) |
| Bermuda | 43.66(35.11 to 53.77) | 51.29(41.06 to 63.36) | 0.49(0.42 to 0.55) | 37.48(31.23 to 45.43) | 38.56(32.42 to 46.37) | 0.14(0.11 to 0.17) |
| Bhutan | 33.79(26.64 to 42.77) | 51.04(38.99 to 66.28) | 1.38(1.32 to 1.43) | 39.05(32.35 to 47.43) | 46.04(37.87 to 56.33) | 0.71(0.62 to 0.79) |
| Bolivia (Plurinational State of) | 10.80(8.61 to 13.73) | 13.02(10.25 to 16.66) | 0.50(0.39 to 0.61) | 10.72(8.92 to 13.05) | 12.04(10.04 to 14.50) | 0.31(0.25 to 0.38) |
| Bosnia and Herzegovina | 57.93(46.35 to 72.50) | 74.61(59.61 to 93.88) | 0.60(0.45 to 0.74) | 47.37(39.27 to 57.73) | 52.63(43.74 to 63.99) | 0.29(0.24 to 0.34) |
| Botswana | 11.27(8.84 to 14.35) | 15.95(12.65 to 20.50) | 1.02(0.97 to 1.07) | 13.02(10.84 to 15.84) | 15.66(13.11 to 19.04) | 0.47(0.41 to 0.54) |
| Brazil | 13.88(11.20 to 17.22) | 23.75(18.88 to 30.03) | 1.36(0.81 to 1.92) | 13.21(11.26 to 15.68) | 21.43(18.06 to 25.81) | 1.18(0.71 to 1.66) |
| Brunei Darussalam | 12.86(10.24 to 16.14) | 15.26(11.89 to 19.04) | 0.49(0.46 to 0.53) | 11.08(9.15 to 13.52) | 11.88(9.73 to 14.38) | 0.15(0.09 to 0.21) |
| Bulgaria | 84.00(67.82 to 104.53) | 96.18(78.19 to 118.18) | 0.45(0.33 to 0.56) | 65.25(55.20 to 79.29) | 65.37(55.12 to 77.66) | 0.01(-0.07 to 0.10) |
| Burkina Faso | 9.53(7.42 to 12.14) | 11.40(9.02 to 14.32) | 0.59(0.55 to 0.63) | 10.53(8.83 to 13.13) | 13.89(11.68 to 16.87) | 0.90(0.86 to 0.94) |
| Burundi | 10.44(8.21 to 13.34) | 10.67(8.43 to 13.86) | -0.26(-0.47 to -0.05) | 12.04(10.01 to 14.75) | 12.30(10.26 to 15.04) | -0.18(-0.29 to -0.07) |
| Cabo Verde | 12.17(9.70 to 15.35) | 17.92(14.10 to 22.12) | 1.08(1.03 to 1.13) | 15.42(13.00 to 18.69) | 19.62(16.64 to 23.57) | 0.82(0.76 to 0.87) |
| Cambodia | 5.42(4.28 to 6.77) | 5.97(4.78 to 7.56) | 0.32(0.26 to 0.38) | 4.57(3.75 to 5.56) | 4.69(3.92 to 5.72) | 0.15(0.10 to 0.19) |
| Cameroon | 10.76(8.52 to 13.66) | 12.51(10.05 to 15.65) | 0.44(0.32 to 0.56) | 12.79(10.58 to 15.62) | 14.56(12.25 to 17.28) | 0.43(0.32 to 0.53) |
| Canada | 622.85(544.59 to 705.91) | 448.58(357.73 to 564.09) | -1.14(-1.56 to -0.71) | 417.15(381.67 to 456.40) | 355.62(298.76 to 425.40) | -0.42(-0.73 to -0.10) |
| Central African Republic | 9.66(7.60 to 12.35) | 10.11(7.92 to 12.89) | 0.06(-0.00 to 0.13) | 10.64(8.87 to 12.97) | 11.13(9.08 to 13.48) | 0.06(0.02 to 0.10) |
| Chad | 9.97(7.70 to 12.84) | 11.22(8.75 to 14.14) | 0.42(0.38 to 0.47) | 10.94(8.97 to 13.54) | 13.55(11.16 to 16.33) | 0.77(0.71 to 0.83) |
| Chile | 51.37(40.29 to 64.27) | 63.61(50.74 to 80.48) | 0.60(0.55 to 0.65) | 52.27(43.17 to 63.05) | 57.70(48.04 to 71.74) | 0.29(0.26 to 0.31) |
| China | 6.54(5.27 to 8.15) | 12.14(9.70 to 15.44) | 3.03(2.25 to 3.81) | 5.90(4.99 to 7.03) | 9.47(8.05 to 11.39) | 2.53(1.86 to 3.21) |
| Colombia | 11.48(9.06 to 15.08) | 13.64(10.66 to 17.04) | 0.46(0.11 to 0.80) | 11.98(9.92 to 14.80) | 12.75(10.39 to 15.48) | 0.13(-0.11 to 0.37) |
| Comoros | 12.40(9.87 to 15.80) | 14.59(11.47 to 18.83) | 0.52(0.45 to 0.59) | 13.50(11.30 to 16.29) | 14.52(12.00 to 17.85) | 0.25(0.09 to 0.41) |
| Congo | 12.27(10.01 to 15.64) | 15.39(12.44 to 19.46) | 0.76(0.64 to 0.88) | 14.93(12.66 to 18.28) | 16.02(13.66 to 19.23) | 0.16(0.05 to 0.27) |
| Cook Islands | 8.44(6.79 to 10.75) | 9.46(7.39 to 11.99) | 0.41(0.33 to 0.50) | 8.07(6.75 to 9.91) | 8.05(6.53 to 9.82) | -0.04(-0.08 to 0.01) |
| Costa Rica | 7.01(5.53 to 8.97) | 8.03(6.19 to 10.48) | 0.29(0.24 to 0.34) | 6.70(5.54 to 8.22) | 6.94(5.69 to 8.55) | 0.06(0.02 to 0.09) |
| Côte d'Ivoire | 10.85(8.56 to 13.58) | 12.59(9.90 to 15.81) | 0.22(-0.51 to 0.95) | 12.60(10.46 to 15.24) | 14.31(12.12 to 17.46) | 0.39(0.29 to 0.48) |
| Croatia | 96.04(75.91 to 120.77) | 115.48(91.19 to 143.31) | 0.67(0.57 to 0.77) | 75.71(62.53 to 91.66) | 83.18(69.40 to 101.66) | 0.05(-0.49 to 0.59) |
| Cuba | 34.68(28.14 to 43.04) | 42.43(34.49 to 52.90) | 0.86(0.79 to 0.94) | 32.91(27.57 to 39.65) | 32.93(27.53 to 39.28) | 0.01(-0.04 to 0.06) |
| Cyprus | 120.68(97.29 to 154.25) | 156.35(125.50 to 195.32) | 1.34(0.86 to 1.83) | 97.21(81.35 to 120.89) | 116.41(98.26 to 141.05) | 0.66(0.59 to 0.73) |
| Czechia | 257.27(210.23 to 316.04) | 306.23(249.28 to 370.66) | 0.36(0.24 to 0.49) | 203.75(172.07 to 241.80) | 210.28(177.67 to 246.28) | 0.61(0.25 to 0.98) |
| Democratic People's Republic of Korea | 8.38(6.72 to 10.42) | 8.02(6.41 to 10.05) | -0.04(-0.10 to 0.01) | 6.76(5.65 to 8.14) | 6.19(5.14 to 7.49) | -0.13(-0.20 to -0.05) |
| Democratic Republic of the Congo | 12.21(9.71 to 15.16) | 11.55(9.06 to 14.88) | -0.45(-0.65 to -0.25) | 14.46(12.21 to 17.42) | 12.51(10.48 to 15.21) | -0.68(-0.92 to -0.44) |
| Denmark | 271.06(227.93 to 322.03) | 240.79(194.03 to 286.44) | -0.50(-0.92 to -0.08) | 197.59(168.36 to 226.96) | 192.02(162.40 to 221.68) | -0.07(-0.34 to 0.19) |
| Djibouti | 12.38(9.78 to 15.64) | 15.63(12.24 to 20.01) | 0.75(0.61 to 0.89) | 13.95(11.56 to 16.87) | 15.19(12.67 to 18.28) | 0.30(0.22 to 0.39) |
| Dominica | 30.16(23.62 to 37.49) | 36.68(28.98 to 46.65) | 0.65(0.59 to 0.70) | 29.59(24.12 to 36.21) | 31.26(25.79 to 38.20) | 0.30(0.23 to 0.38) |
| Dominican Republic | 26.79(21.04 to 33.17) | 32.53(26.13 to 40.95) | 0.59(0.55 to 0.63) | 27.48(22.69 to 33.67) | 28.28(23.52 to 34.28) | 0.18(0.07 to 0.28) |
| Ecuador | 12.45(9.90 to 15.73) | 14.91(11.81 to 18.51) | 0.58(0.51 to 0.64) | 13.32(11.02 to 16.24) | 14.22(12.05 to 17.23) | 0.27(0.17 to 0.38) |
| Egypt | 39.96(32.94 to 47.83) | 48.47(38.15 to 61.53) | 0.95(0.78 to 1.13) | 35.34(30.26 to 40.99) | 42.62(35.14 to 52.06) | 0.96(0.78 to 1.14) |
| El Salvador | 4.80(3.72 to 6.18) | 6.32(4.90 to 8.08) | 0.95(0.88 to 1.03) | 4.80(3.89 to 5.75) | 5.94(4.95 to 7.18) | 0.79(0.73 to 0.86) |
| Equatorial Guinea | 10.79(8.51 to 13.73) | 15.47(12.34 to 19.61) | 1.31(1.16 to 1.46) | 11.63(9.84 to 14.11) | 16.78(13.94 to 20.24) | 1.24(0.98 to 1.50) |
| Eritrea | 12.27(9.63 to 15.54) | 13.44(10.46 to 17.22) | 0.08(-0.01 to 0.16) | 13.05(10.75 to 15.74) | 13.80(11.42 to 16.83) | -0.06(-0.15 to 0.03) |
| Estonia | 48.35(39.16 to 60.74) | 61.52(49.56 to 77.20) | 0.68(0.56 to 0.81) | 43.64(36.68 to 52.65) | 46.35(39.23 to 56.31) | 0.33(0.17 to 0.48) |
| Eswatini | 10.47(8.24 to 13.01) | 13.46(10.63 to 16.94) | 0.70(0.64 to 0.76) | 12.20(10.27 to 14.72) | 14.80(12.44 to 18.00) | 0.58(0.53 to 0.64) |
| Ethiopia | 8.37(6.75 to 10.58) | 9.60(7.81 to 11.85) | 0.25(0.05 to 0.44) | 10.36(8.74 to 12.45) | 12.17(10.33 to 14.54) | 0.32(0.16 to 0.48) |
| Fiji | 7.11(5.64 to 9.15) | 7.23(5.77 to 9.31) | 0.02(-0.02 to 0.05) | 6.54(5.44 to 7.95) | 6.21(5.24 to 7.61) | -0.13(-0.18 to -0.08) |
| Finland | 247.54(219.02 to 277.13) | 216.15(177.58 to 264.39) | -1.40(-1.97 to -0.82) | 171.74(154.65 to 190.29) | 184.97(156.99 to 217.27) | -0.48(-1.01 to 0.05) |
| France | 180.74(165.51 to 197.06) | 171.30(139.62 to 209.03) | -0.55(-0.83 to -0.27) | 123.46(113.46 to 132.96) | 139.83(118.89 to 166.94) | 0.23(0.03 to 0.44) |
| Gabon | 12.44(10.23 to 15.27) | 15.22(12.16 to 18.94) | 0.53(0.47 to 0.59) | 14.92(12.77 to 17.79) | 16.38(13.73 to 19.28) | 0.17(0.12 to 0.22) |
| Gambia | 10.85(8.71 to 13.45) | 12.31(9.89 to 15.88) | 0.38(0.34 to 0.41) | 13.59(11.59 to 16.07) | 14.62(12.13 to 18.14) | 0.28(0.22 to 0.34) |
| Georgia | 53.35(42.21 to 68.01) | 62.65(50.65 to 78.43) | 0.49(0.44 to 0.55) | 52.59(43.65 to 63.60) | 52.69(44.44 to 63.39) | 0.08(-0.00 to 0.16) |
| Germany | 303.82(237.87 to 371.29) | 356.34(283.40 to 452.14) | 0.65(0.18 to 1.12) | 219.75(180.10 to 262.40) | 264.74(221.69 to 321.35) | 0.69(0.29 to 1.09) |
| Ghana | 13.36(10.45 to 16.50) | 16.73(13.40 to 21.06) | 0.51(0.42 to 0.59) | 15.72(13.08 to 18.79) | 19.07(15.88 to 23.01) | 0.51(0.48 to 0.54) |
| Greece | 41.67(34.99 to 49.19) | 54.50(43.74 to 69.03) | 1.00(0.37 to 1.64) | 32.13(27.97 to 36.58) | 39.90(33.42 to 48.41) | 0.61(0.04 to 1.19) |
| Greenland | 261.23(209.71 to 320.16) | 257.69(210.49 to 316.26) | -0.23(-0.31 to -0.14) | 225.74(191.41 to 268.17) | 214.23(182.46 to 250.89) | -0.15(-0.19 to -0.12) |
| Grenada | 26.53(21.01 to 33.08) | 33.85(27.20 to 41.88) | 0.84(0.81 to 0.86) | 25.83(21.40 to 31.89) | 27.97(23.36 to 33.56) | 0.27(0.25 to 0.30) |
| Guam | 9.06(7.00 to 11.75) | 9.73(7.42 to 12.29) | 0.12(0.04 to 0.20) | 8.26(6.83 to 10.12) | 8.16(6.72 to 9.92) | -0.05(-0.10 to -0.00) |
| Guatemala | 4.44(3.46 to 5.82) | 5.20(3.98 to 6.56) | 0.49(0.41 to 0.57) | 4.42(3.66 to 5.47) | 5.02(4.10 to 6.11) | 0.39(0.36 to 0.42) |
| Guinea | 10.75(8.52 to 13.89) | 11.98(9.58 to 15.09) | 0.31(0.21 to 0.41) | 11.35(9.47 to 13.79) | 14.11(11.76 to 16.97) | 0.77(0.69 to 0.84) |
| Guinea-Bissau | 10.34(8.23 to 13.20) | 12.52(9.86 to 16.01) | 0.60(0.53 to 0.68) | 11.51(9.49 to 13.98) | 13.99(11.67 to 17.03) | 0.69(0.64 to 0.74) |
| Guyana | 28.24(21.90 to 35.81) | 31.91(24.92 to 41.24) | 0.21(0.15 to 0.28) | 26.66(21.87 to 32.04) | 27.56(22.39 to 34.25) | 0.07(-0.01 to 0.14) |
| Haiti | 22.53(17.95 to 28.24) | 26.29(20.24 to 33.65) | 0.52(0.34 to 0.69) | 21.28(17.47 to 25.71) | 22.33(17.99 to 27.34) | 0.19(0.12 to 0.26) |
| Honduras | 4.43(3.43 to 5.72) | 5.24(4.09 to 6.78) | 0.50(0.43 to 0.57) | 4.47(3.64 to 5.44) | 5.01(4.13 to 6.12) | 0.34(0.31 to 0.37) |
| Hungary | 224.39(196.05 to 256.52) | 231.74(187.65 to 283.76) | 0.10(-0.26 to 0.47) | 165.42(147.03 to 186.70) | 164.51(138.82 to 194.31) | -0.10(-0.43 to 0.23) |
| Iceland | 290.13(251.67 to 330.48) | 239.74(193.91 to 290.13) | -1.01(-1.19 to -0.84) | 233.64(206.87 to 261.67) | 198.76(165.79 to 232.23) | -0.77(-0.90 to -0.63) |
| India | 32.39(26.27 to 39.78) | 43.42(35.30 to 53.67) | 1.29(1.07 to 1.51) | 39.38(33.52 to 47.33) | 48.15(40.81 to 57.88) | 0.88(0.75 to 1.01) |
| Indonesia | 6.49(5.24 to 8.19) | 7.00(5.52 to 8.94) | 0.26(0.22 to 0.29) | 6.04(5.09 to 7.21) | 5.91(4.91 to 7.14) | -0.11(-0.17 to -0.04) |
| Iran (Islamic Republic of) | 29.06(22.91 to 37.69) | 37.65(29.85 to 47.51) | 1.53(1.18 to 1.88) | 35.24(29.14 to 43.86) | 35.07(29.28 to 42.75) | 0.58(0.25 to 0.90) |
| Iraq | 22.43(18.29 to 27.81) | 33.20(26.18 to 42.15) | 1.36(1.28 to 1.44) | 20.71(17.58 to 24.77) | 28.37(23.18 to 34.28) | 1.08(0.96 to 1.20) |
| Ireland | 181.67(145.10 to 227.55) | 216.22(173.98 to 268.08) | 0.85(0.73 to 0.98) | 153.28(127.80 to 183.74) | 168.51(141.40 to 200.17) | 0.55(0.41 to 0.70) |
| Israel | 148.89(118.66 to 183.85) | 167.78(133.85 to 212.53) | -0.40(-1.13 to 0.33) | 128.27(107.42 to 153.74) | 137.17(113.01 to 166.20) | -0.53(-1.09 to 0.04) |
| Italy | 122.08(102.26 to 147.42) | 97.62(78.70 to 120.59) | -0.53(-0.77 to -0.28) | 103.52(89.52 to 121.63) | 82.72(70.46 to 100.08) | -0.78(-0.98 to -0.57) |
| Jamaica | 30.12(24.07 to 38.02) | 37.91(30.39 to 47.52) | 0.64(0.55 to 0.73) | 31.18(25.86 to 37.44) | 33.60(28.19 to 40.52) | 0.27(0.23 to 0.30) |
| Japan | 30.20(24.32 to 37.59) | 34.35(27.61 to 42.79) | 0.09(-0.48 to 0.67) | 26.88(22.63 to 32.51) | 28.62(24.16 to 34.71) | -0.13(-0.62 to 0.37) |
| Jordan | 36.75(30.53 to 44.33) | 47.36(38.59 to 59.32) | 1.32(1.12 to 1.52) | 40.15(34.57 to 46.59) | 45.43(38.33 to 55.99) | 0.77(0.59 to 0.96) |
| Kazakhstan | 47.69(37.76 to 60.11) | 60.43(48.02 to 76.52) | 0.67(0.63 to 0.72) | 49.99(41.63 to 61.00) | 51.01(42.33 to 62.62) | 0.15(0.04 to 0.26) |
| Kenya | 9.39(7.68 to 11.63) | 11.67(9.48 to 14.46) | 0.61(0.55 to 0.66) | 13.29(11.28 to 16.01) | 14.86(12.59 to 17.64) | 0.15(0.08 to 0.23) |
| Kiribati | 6.28(4.94 to 8.16) | 6.35(4.92 to 8.12) | -0.02(-0.07 to 0.03) | 5.72(4.68 to 7.12) | 5.60(4.59 to 6.90) | -0.05(-0.09 to -0.02) |
| Kuwait | 65.31(55.82 to 78.19) | 65.15(51.64 to 82.73) | -0.07(-0.61 to 0.47) | 57.74(50.85 to 65.90) | 49.58(41.10 to 60.48) | -0.39(-0.76 to -0.03) |
| Kyrgyzstan | 39.93(31.61 to 50.68) | 48.58(38.87 to 61.33) | 0.47(0.38 to 0.57) | 44.65(36.94 to 55.04) | 46.67(39.31 to 57.06) | 0.09(-0.02 to 0.20) |
| Lao People's Democratic Republic | 5.42(4.20 to 6.92) | 6.63(5.21 to 8.46) | 0.76(0.60 to 0.92) | 4.60(3.75 to 5.66) | 5.19(4.27 to 6.47) | 0.54(0.44 to 0.63) |
| Latvia | 50.39(41.31 to 63.64) | 62.40(48.81 to 79.09) | 0.65(0.53 to 0.76) | 46.30(38.95 to 56.28) | 47.25(38.84 to 57.07) | 0.18(0.02 to 0.33) |
| Lebanon | 85.57(69.36 to 107.95) | 118.56(96.29 to 145.40) | 0.97(0.84 to 1.10) | 82.85(70.26 to 102.25) | 97.62(82.79 to 117.88) | 0.71(0.62 to 0.81) |
| Lesotho | 10.99(8.63 to 13.88) | 12.71(10.33 to 15.86) | 0.35(0.30 to 0.39) | 11.75(9.78 to 14.25) | 14.28(12.00 to 17.30) | 0.57(0.53 to 0.62) |
| Liberia | 10.28(8.01 to 12.90) | 12.70(10.04 to 15.96) | 0.73(0.60 to 0.86) | 11.50(9.64 to 13.86) | 14.04(11.76 to 16.94) | 0.92(0.79 to 1.05) |
| Libya | 18.40(14.77 to 23.55) | 40.05(32.02 to 50.38) | 2.98(2.67 to 3.29) | 19.79(16.39 to 24.39) | 31.56(26.44 to 38.48) | 1.84(1.44 to 2.25) |
| Lithuania | 57.60(46.21 to 72.62) | 74.28(59.73 to 93.37) | 1.28(0.58 to 1.98) | 55.00(46.17 to 67.38) | 58.53(47.96 to 71.56) | 0.65(0.09 to 1.22) |
| Luxembourg | 165.55(133.11 to 209.12) | 196.68(157.82 to 244.95) | 0.42(0.32 to 0.52) | 128.41(108.80 to 153.74) | 156.35(133.11 to 189.42) | 0.53(0.46 to 0.61) |
| Madagascar | 10.62(8.23 to 13.36) | 11.83(9.53 to 14.87) | 0.19(0.07 to 0.31) | 12.17(10.14 to 14.83) | 12.99(11.06 to 15.81) | -0.03(-0.17 to 0.12) |
| Malawi | 9.43(7.38 to 11.85) | 11.61(9.24 to 14.98) | 0.72(0.68 to 0.77) | 11.30(9.41 to 13.66) | 12.95(10.72 to 15.85) | 0.32(0.27 to 0.37) |
| Malaysia | 8.56(6.97 to 10.80) | 12.45(9.95 to 15.74) | 1.80(1.44 to 2.16) | 7.08(5.97 to 8.51) | 9.11(7.55 to 11.08) | 1.43(1.10 to 1.76) |
| Maldives | 5.57(4.44 to 7.01) | 8.62(6.77 to 11.15) | 1.38(1.31 to 1.44) | 5.12(4.22 to 6.33) | 6.50(5.28 to 7.98) | 0.62(0.55 to 0.70) |
| Mali | 9.66(7.62 to 12.32) | 10.78(8.54 to 13.26) | 0.37(0.31 to 0.43) | 10.50(8.77 to 12.90) | 13.09(10.85 to 15.66) | 0.80(0.72 to 0.88) |
| Malta | 120.84(95.91 to 151.46) | 131.42(107.07 to 166.06) | 0.11(-0.01 to 0.22) | 92.16(75.77 to 110.82) | 99.27(83.41 to 121.30) | 0.16(0.10 to 0.22) |
| Marshall Islands | 5.65(4.51 to 7.13) | 6.28(4.95 to 7.93) | 0.36(0.34 to 0.38) | 5.52(4.59 to 6.60) | 5.59(4.57 to 6.79) | 0.02(-0.02 to 0.06) |
| Mauritania | 12.31(9.74 to 15.61) | 15.34(12.14 to 19.63) | 0.56(0.50 to 0.62) | 13.47(11.31 to 16.47) | 18.07(15.11 to 22.18) | 0.74(0.63 to 0.86) |
| Mauritius | 8.03(6.31 to 10.17) | 9.47(7.43 to 12.14) | 0.58(0.52 to 0.64) | 6.59(5.48 to 8.08) | 7.16(5.92 to 8.88) | 0.36(0.32 to 0.41) |
| Mexico | 1.99(1.55 to 2.56) | 2.20(1.71 to 2.84) | 0.59(0.37 to 0.81) | 1.94(1.63 to 2.38) | 1.88(1.56 to 2.32) | 0.14(-0.09 to 0.38) |
| Micronesia (Federated States of) | 6.29(4.92 to 8.14) | 6.51(5.10 to 8.34) | 0.03(-0.01 to 0.07) | 6.01(4.87 to 7.34) | 5.90(4.87 to 7.28) | -0.14(-0.18 to -0.09) |
| Monaco | 196.29(159.08 to 246.52) | 202.49(163.71 to 248.67) | 0.11(0.05 to 0.17) | 146.18(124.05 to 178.66) | 157.07(132.00 to 188.14) | 0.26(0.24 to 0.28) |
| Mongolia | 33.99(27.46 to 42.24) | 47.49(37.60 to 59.61) | 1.12(1.09 to 1.16) | 38.73(32.50 to 46.47) | 39.27(32.77 to 47.11) | 0.06(0.03 to 0.10) |
| Montenegro | 81.69(66.27 to 101.02) | 96.70(76.28 to 118.06) | 0.55(0.52 to 0.59) | 68.13(57.52 to 82.18) | 73.63(60.73 to 87.64) | 0.29(0.27 to 0.32) |
| Morocco | 28.46(22.69 to 35.91) | 40.42(32.29 to 51.61) | 1.31(1.22 to 1.40) | 26.15(21.78 to 32.21) | 32.68(27.16 to 40.09) | 0.95(0.81 to 1.10) |
| Mozambique | 9.24(7.48 to 11.40) | 9.76(7.62 to 12.36) | 0.15(0.09 to 0.20) | 10.41(8.73 to 12.46) | 11.52(9.61 to 13.88) | 0.19(0.11 to 0.27) |
| Myanmar | 5.60(4.30 to 7.02) | 6.95(5.45 to 8.84) | 0.96(0.86 to 1.06) | 4.76(3.91 to 5.75) | 5.31(4.38 to 6.52) | 0.56(0.48 to 0.64) |
| Namibia | 14.79(11.84 to 18.68) | 16.50(13.00 to 20.59) | 0.11(0.02 to 0.21) | 15.80(13.16 to 19.11) | 17.57(14.85 to 21.08) | 0.23(0.16 to 0.31) |
| Nauru | 6.95(5.34 to 9.03) | 6.80(5.29 to 8.71) | -0.16(-0.23 to -0.09) | 6.21(5.08 to 7.64) | 6.13(5.05 to 7.49) | -0.09(-0.17 to -0.01) |
| Nepal | 39.27(30.50 to 50.13) | 47.71(37.27 to 61.42) | 0.40(0.31 to 0.48) | 41.24(33.99 to 49.88) | 46.27(37.33 to 57.62) | 0.32(0.27 to 0.36) |
| Netherlands | 315.01(276.34 to 357.87) | 357.97(295.48 to 433.25) | 0.32(-0.45 to 1.10) | 234.88(209.65 to 261.65) | 287.83(245.76 to 339.59) | 0.67(0.04 to 1.31) |
| New Zealand | 211.19(170.74 to 261.23) | 213.84(173.07 to 270.49) | 0.13(-0.13 to 0.39) | 213.45(183.35 to 253.49) | 200.12(171.98 to 239.78) | -0.12(-0.25 to 0.01) |
| Nicaragua | 4.80(3.72 to 6.19) | 5.89(4.56 to 7.60) | 0.65(0.58 to 0.73) | 4.92(4.08 to 5.99) | 5.38(4.45 to 6.61) | 0.28(0.20 to 0.36) |
| Niger | 10.05(8.01 to 12.83) | 10.42(8.38 to 12.79) | 0.07(0.02 to 0.13) | 11.19(9.25 to 13.71) | 13.11(10.95 to 15.74) | 0.48(0.44 to 0.51) |
| Nigeria | 10.24(8.38 to 12.69) | 12.73(10.39 to 15.74) | 0.79(0.76 to 0.83) | 13.81(11.69 to 16.68) | 17.94(15.31 to 21.67) | 0.92(0.85 to 0.99) |
| Niue | 8.52(6.72 to 11.10) | 8.97(7.00 to 11.44) | 0.16(0.13 to 0.19) | 7.71(6.33 to 9.43) | 7.49(6.29 to 9.20) | -0.13(-0.17 to -0.09) |
| North Macedonia | 75.68(60.85 to 94.54) | 90.64(72.73 to 111.87) | 0.49(0.42 to 0.55) | 60.11(49.56 to 73.16) | 63.57(53.64 to 75.73) | 0.11(0.06 to 0.17) |
| Northern Mariana Islands | 9.39(7.21 to 11.99) | 10.12(7.85 to 12.84) | 0.14(-0.03 to 0.32) | 8.73(7.19 to 10.64) | 8.17(6.72 to 10.06) | -0.29(-0.35 to -0.24) |
| Norway | 224.77(184.49 to 270.55) | 261.58(215.63 to 321.91) | 0.26(0.14 to 0.37) | 212.80(182.51 to 247.58) | 243.87(211.87 to 284.20) | 0.20(0.10 to 0.29) |
| Oman | 34.53(27.55 to 43.19) | 51.93(39.77 to 65.82) | 1.45(1.19 to 1.70) | 33.15(27.32 to 40.59) | 42.52(34.50 to 51.03) | 1.03(0.85 to 1.21) |
| Pakistan | 30.28(24.54 to 37.67) | 34.38(27.42 to 43.61) | 0.55(0.39 to 0.71) | 37.88(32.13 to 45.66) | 39.38(33.85 to 47.50) | 0.32(0.21 to 0.43) |
| Palau | 8.59(6.86 to 10.78) | 9.40(7.31 to 11.94) | 0.17(0.09 to 0.25) | 7.79(6.52 to 9.51) | 7.41(6.15 to 9.03) | -0.19(-0.23 to -0.14) |
| Palestine | 27.41(22.08 to 34.55) | 37.09(29.43 to 46.37) | 1.04(0.95 to 1.13) | 28.98(23.90 to 36.09) | 34.23(28.05 to 41.73) | 0.81(0.66 to 0.95) |
| Panama | 9.17(7.09 to 11.66) | 10.51(8.12 to 13.91) | 0.38(0.34 to 0.42) | 8.88(7.25 to 10.97) | 9.60(7.76 to 11.84) | 0.19(0.16 to 0.22) |
| Papua New Guinea | 6.23(4.83 to 7.93) | 6.28(4.88 to 7.96) | -0.12(-0.21 to -0.03) | 5.82(4.79 to 7.25) | 5.56(4.58 to 6.83) | -0.23(-0.30 to -0.16) |
| Paraguay | 17.39(13.67 to 22.04) | 16.23(12.97 to 20.50) | -0.06(-0.28 to 0.16) | 16.57(13.58 to 20.03) | 13.70(11.51 to 16.40) | -0.33(-0.56 to -0.10) |
| Peru | 14.35(11.42 to 18.23) | 16.04(12.70 to 20.26) | 0.26(0.04 to 0.48) | 14.40(11.96 to 17.38) | 14.25(11.87 to 17.31) | -0.12(-0.30 to 0.05) |
| Philippines | 5.12(4.06 to 6.52) | 4.92(3.85 to 6.32) | -0.18(-0.21 to -0.14) | 5.07(4.23 to 6.16) | 4.40(3.65 to 5.33) | -0.48(-0.52 to -0.45) |
| Poland | 33.06(26.82 to 40.56) | 37.90(30.76 to 46.93) | 0.48(0.40 to 0.56) | 27.55(23.35 to 33.52) | 27.90(23.78 to 33.75) | 0.17(0.10 to 0.25) |
| Portugal | 93.05(75.05 to 115.56) | 114.45(91.14 to 144.79) | 0.93(0.56 to 1.30) | 75.85(63.12 to 90.83) | 83.78(70.68 to 100.62) | 0.42(0.22 to 0.61) |
| Puerto Rico | 35.75(28.49 to 44.83) | 41.65(33.64 to 52.38) | 0.42(0.29 to 0.55) | 32.13(26.84 to 39.05) | 33.30(28.27 to 39.98) | 0.16(0.09 to 0.22) |
| Qatar | 44.34(34.57 to 57.23) | 62.26(48.72 to 79.03) | 1.06(1.02 to 1.10) | 38.82(31.79 to 48.32) | 47.40(39.23 to 58.44) | 0.85(0.72 to 0.99) |
| Republic of Korea | 45.49(37.42 to 56.80) | 68.02(54.73 to 84.36) | 2.34(1.39 to 3.30) | 39.63(34.10 to 47.26) | 53.75(45.01 to 64.69) | 1.72(1.07 to 2.38) |
| Republic of Moldova | 43.40(33.47 to 56.67) | 52.67(41.65 to 66.98) | 0.65(0.47 to 0.84) | 38.07(31.12 to 47.77) | 37.77(30.90 to 46.38) | 0.18(-0.02 to 0.38) |
| Romania | 21.16(17.05 to 26.65) | 25.20(20.12 to 31.44) | 1.22(0.34 to 2.12) | 16.44(13.72 to 19.94) | 17.28(14.49 to 20.59) | 0.67(-0.15 to 1.49) |
| Russian Federation | 37.17(30.10 to 46.35) | 45.86(36.80 to 57.43) | 0.53(0.37 to 0.69) | 38.22(32.44 to 46.27) | 37.89(32.05 to 45.74) | 0.05(-0.14 to 0.25) |
| Rwanda | 10.65(8.47 to 13.26) | 12.23(9.70 to 15.37) | 0.32(0.17 to 0.47) | 12.57(10.39 to 15.29) | 13.28(11.17 to 16.07) | 0.03(-0.08 to 0.13) |
| Saint Kitts and Nevis | 31.56(25.37 to 40.46) | 41.95(32.98 to 52.82) | 0.87(0.82 to 0.92) | 30.75(25.67 to 37.87) | 32.18(26.69 to 38.80) | 0.28(0.17 to 0.39) |
| Saint Lucia | 28.66(23.07 to 35.96) | 40.48(31.91 to 51.72) | 1.11(1.04 to 1.17) | 27.99(23.29 to 34.25) | 31.56(25.90 to 39.46) | 0.33(0.26 to 0.40) |
| Saint Vincent and the Grenadines | 26.78(21.20 to 34.20) | 35.14(27.91 to 44.68) | 0.90(0.87 to 0.93) | 26.90(22.45 to 32.87) | 28.10(23.36 to 34.07) | 0.20(0.16 to 0.25) |
| Samoa | 6.45(5.18 to 8.09) | 6.99(5.53 to 8.70) | 0.20(0.10 to 0.29) | 6.73(5.58 to 8.07) | 6.65(5.48 to 7.96) | -0.12(-0.18 to -0.06) |
| San Marino | 230.18(187.04 to 291.04) | 269.17(216.23 to 335.82) | 0.73(0.57 to 0.90) | 195.39(165.32 to 239.01) | 201.30(168.23 to 243.44) | 0.21(0.10 to 0.33) |
| Sao Tome and Principe | 12.41(9.88 to 15.82) | 16.05(13.08 to 20.70) | 0.92(0.82 to 1.01) | 15.71(13.13 to 19.19) | 18.89(15.68 to 23.58) | 0.65(0.60 to 0.70) |
| Saudi Arabia | 23.74(18.81 to 29.61) | 38.84(30.89 to 50.70) | 1.45(1.35 to 1.55) | 22.22(18.52 to 27.21) | 30.65(25.31 to 38.18) | 1.22(1.06 to 1.38) |
| Senegal | 10.59(8.45 to 13.30) | 13.97(11.06 to 17.74) | 0.78(0.73 to 0.83) | 12.47(10.54 to 15.05) | 15.48(12.91 to 18.89) | 0.49(0.37 to 0.62) |
| Serbia | 81.92(66.05 to 102.10) | 95.35(76.41 to 119.73) | 0.57(0.48 to 0.66) | 64.48(53.67 to 78.08) | 68.63(57.82 to 84.61) | 0.32(0.20 to 0.45) |
| Seychelles | 7.65(6.06 to 9.65) | 9.33(7.37 to 11.97) | 0.70(0.61 to 0.78) | 6.76(5.58 to 8.34) | 7.09(5.91 to 8.73) | 0.22(0.19 to 0.26) |
| Sierra Leone | 10.33(8.12 to 13.36) | 11.67(9.46 to 14.56) | 0.25(0.17 to 0.33) | 11.25(9.35 to 13.66) | 13.79(11.60 to 16.85) | 0.69(0.59 to 0.78) |
| Singapore | 13.08(10.67 to 16.62) | 16.41(12.95 to 21.01) | 0.42(0.30 to 0.55) | 9.81(8.26 to 12.06) | 11.01(9.14 to 13.29) | 0.15(0.04 to 0.26) |
| Slovakia | 84.49(68.21 to 104.83) | 104.33(83.62 to 129.06) | 0.65(0.58 to 0.71) | 70.55(59.45 to 85.17) | 72.11(61.07 to 86.67) | -0.00(-0.05 to 0.05) |
| Slovenia | 123.83(99.53 to 152.13) | 152.93(121.77 to 188.61) | 0.65(0.42 to 0.89) | 100.35(83.49 to 120.98) | 106.54(89.40 to 126.44) | 0.14(-0.03 to 0.30) |
| Solomon Islands | 5.39(4.16 to 6.80) | 5.78(4.57 to 7.39) | 0.25(0.22 to 0.27) | 5.25(4.31 to 6.37) | 5.17(4.27 to 6.28) | -0.09(-0.14 to -0.05) |
| Somalia | 10.24(7.99 to 12.91) | 10.41(8.32 to 13.33) | -0.19(-0.27 to -0.12) | 10.89(9.02 to 13.36) | 11.06(9.09 to 13.46) | -0.19(-0.30 to -0.09) |
| South Africa | 11.87(9.58 to 14.86) | 15.77(12.85 to 19.73) | 0.77(0.71 to 0.83) | 15.54(13.21 to 18.68) | 18.29(15.62 to 21.78) | 0.43(0.38 to 0.47) |
| South Sudan | 10.93(8.61 to 13.97) | 11.56(9.37 to 14.71) | 0.03(-0.05 to 0.10) | 12.21(10.16 to 14.78) | 12.22(10.19 to 14.84) | -0.26(-0.33 to -0.18) |
| Spain | 137.08(117.38 to 160.32) | 150.59(122.20 to 187.31) | 0.24(-0.45 to 0.94) | 111.08(98.17 to 126.47) | 112.83(95.21 to 136.95) | -0.08(-0.61 to 0.45) |
| Sri Lanka | 10.22(8.10 to 13.05) | 11.59(9.11 to 14.51) | 0.90(0.54 to 1.27) | 8.62(7.11 to 10.57) | 9.28(7.61 to 11.36) | 0.55(0.25 to 0.85) |
| Sudan | 24.79(19.93 to 30.98) | 34.88(27.29 to 44.92) | 1.30(1.18 to 1.42) | 23.74(19.68 to 28.74) | 31.78(26.09 to 39.03) | 1.19(1.03 to 1.35) |
| Suriname | 29.97(23.75 to 37.63) | 36.07(28.90 to 46.21) | 0.63(0.60 to 0.65) | 28.72(24.10 to 34.95) | 29.48(24.50 to 36.56) | 0.12(0.08 to 0.16) |
| Sweden | 262.02(220.24 to 310.05) | 252.98(210.91 to 302.19) | -0.11(-0.29 to 0.08) | 233.23(202.88 to 266.39) | 228.14(200.55 to 263.60) | -0.02(-0.15 to 0.11) |
| Switzerland | 202.09(157.68 to 257.43) | 203.77(161.01 to 255.63) | -0.06(-0.15 to 0.04) | 153.79(127.93 to 189.43) | 151.44(125.66 to 183.17) | -0.04(-0.07 to -0.01) |
| Syrian Arab Republic | 27.78(22.11 to 35.33) | 41.56(32.71 to 52.60) | 1.67(1.55 to 1.79) | 28.05(23.29 to 34.98) | 36.21(29.97 to 43.81) | 1.11(0.85 to 1.38) |
| Taiwan (Province of China) | 7.85(6.10 to 9.82) | 9.00(6.98 to 11.58) | 1.98(0.98 to 2.99) | 6.58(5.42 to 7.96) | 6.68(5.55 to 8.16) | 1.51(0.51 to 2.52) |
| Tajikistan | 34.32(27.43 to 42.35) | 42.80(34.18 to 52.68) | 0.63(0.56 to 0.71) | 40.60(34.01 to 49.29) | 40.69(33.84 to 48.73) | 0.11(0.04 to 0.19) |
| Thailand | 6.28(5.06 to 7.83) | 8.01(6.35 to 10.02) | 0.88(0.77 to 1.00) | 5.47(4.57 to 6.47) | 6.08(5.09 to 7.36) | 0.43(0.38 to 0.49) |
| Timor-Leste | 5.72(4.47 to 7.31) | 6.16(4.93 to 7.77) | 0.25(0.19 to 0.32) | 4.73(3.90 to 5.85) | 5.29(4.36 to 6.48) | 0.47(0.42 to 0.53) |
| Togo | 11.85(9.44 to 15.27) | 14.72(11.69 to 18.64) | 0.68(0.58 to 0.77) | 13.65(11.52 to 16.58) | 15.16(12.74 to 18.06) | 0.29(0.23 to 0.35) |
| Tokelau | 6.97(5.44 to 8.79) | 8.10(6.39 to 10.12) | 0.53(0.47 to 0.59) | 6.16(5.10 to 7.50) | 6.97(5.83 to 8.42) | 0.49(0.42 to 0.55) |
| Tonga | 7.05(5.45 to 8.88) | 7.94(6.40 to 10.11) | 0.40(0.38 to 0.42) | 7.10(5.88 to 8.72) | 7.26(6.07 to 8.95) | 0.04(-0.01 to 0.08) |
| Trinidad and Tobago | 34.71(27.17 to 44.16) | 43.89(34.90 to 55.26) | 0.89(0.79 to 0.99) | 31.10(25.64 to 38.55) | 33.50(28.10 to 40.51) | 0.53(0.41 to 0.64) |
| Tunisia | 29.34(23.37 to 37.51) | 47.65(38.44 to 58.30) | 1.69(1.62 to 1.75) | 30.82(25.51 to 37.67) | 40.23(33.90 to 48.07) | 1.06(0.92 to 1.19) |
| Türkiye | 29.32(23.04 to 37.45) | 43.14(34.98 to 52.83) | 0.69(0.60 to 0.77) | 26.99(22.40 to 33.03) | 36.77(30.87 to 43.58) | 1.29(0.85 to 1.74) |
| Turkmenistan | 37.69(30.18 to 46.66) | 47.13(37.35 to 59.53) | -0.35(-0.42 to -0.29) | 41.70(34.20 to 50.48) | 42.54(35.77 to 50.91) | 0.14(0.09 to 0.19) |
| Tuvalu | 6.85(5.26 to 8.94) | 6.42(4.94 to 8.12) | 1.45(1.06 to 1.84) | 5.83(4.77 to 7.10) | 5.71(4.71 to 6.97) | -0.08(-0.12 to -0.04) |
| Uganda | 8.97(7.02 to 11.36) | 11.05(8.74 to 13.88) | 0.68(0.59 to 0.76) | 10.91(9.01 to 13.20) | 13.21(11.34 to 15.60) | 0.63(0.57 to 0.69) |
| Ukraine | 37.91(29.83 to 47.53) | 41.22(32.46 to 52.33) | 0.22(0.13 to 0.31) | 36.36(30.20 to 44.05) | 31.54(26.60 to 38.04) | -0.37(-0.48 to -0.26) |
| United Arab Emirates | 45.50(35.84 to 56.97) | 70.88(55.07 to 90.81) | 1.59(1.38 to 1.79) | 40.99(33.47 to 49.80) | 45.71(37.62 to 55.77) | 0.54(0.37 to 0.70) |
| United Kingdom | 154.55(125.99 to 187.92) | 173.54(141.65 to 211.64) | -0.34(-0.59 to -0.09) | 132.67(113.98 to 158.33) | 148.55(127.54 to 172.81) | -0.20(-0.41 to 0.01) |
| United Republic of Tanzania | 9.94(8.06 to 12.34) | 12.88(10.25 to 16.23) | 0.69(0.59 to 0.80) | 12.45(10.44 to 15.16) | 14.60(12.19 to 17.48) | 0.37(0.28 to 0.47) |
| United States of America | 189.86(154.71 to 233.57) | 187.09(153.55 to 230.65) | -0.14(-0.35 to 0.07) | 179.55(154.91 to 213.30) | 185.45(160.97 to 216.04) | 0.18(0.05 to 0.32) |
| United States Virgin Islands | 43.17(34.11 to 54.12) | 50.73(40.76 to 64.23) | 0.50(0.47 to 0.53) | 36.06(29.81 to 43.58) | 38.83(32.31 to 47.09) | 0.27(0.24 to 0.29) |
| Uruguay | 59.13(46.36 to 77.56) | 62.19(48.37 to 80.34) | 0.15(0.10 to 0.20) | 55.61(46.41 to 67.57) | 53.24(43.75 to 64.97) | -0.17(-0.20 to -0.14) |
| Uzbekistan | 37.47(29.74 to 47.32) | 49.18(39.14 to 62.58) | 0.84(0.75 to 0.92) | 44.43(36.53 to 54.00) | 42.06(35.11 to 50.83) | 0.00(-0.09 to 0.10) |
| Vanuatu | 6.29(4.98 to 7.93) | 6.44(5.05 to 8.35) | 0.01(-0.03 to 0.06) | 5.75(4.80 to 7.00) | 5.72(4.70 to 7.02) | -0.04(-0.09 to 0.02) |
| Venezuela (Bolivarian Republic of) | 9.77(7.70 to 12.21) | 9.83(7.52 to 12.66) | 0.21(0.06 to 0.35) | 9.84(8.25 to 11.70) | 8.22(6.70 to 10.04) | -0.20(-0.39 to 0.00) |
| Viet Nam | 6.50(5.14 to 8.11) | 8.50(6.82 to 10.53) | 0.91(0.89 to 0.92) | 6.04(5.05 to 7.26) | 6.47(5.30 to 7.86) | 0.36(0.31 to 0.40) |
| Yemen | 22.53(18.28 to 28.13) | 30.41(23.90 to 38.10) | 1.19(1.06 to 1.32) | 21.89(18.12 to 26.48) | 28.18(23.14 to 34.83) | 1.07(0.90 to 1.24) |
| Zambia | 9.28(7.52 to 11.50) | 11.41(9.10 to 14.42) | 0.50(0.36 to 0.64) | 11.75(9.80 to 14.10) | 13.67(11.59 to 16.63) | 0.28(0.14 to 0.41) |
| Zimbabwe | 11.16(8.81 to 14.31) | 12.77(10.16 to 16.23) | 0.01(-0.20 to 0.22) | 12.48(10.55 to 15.30) | 14.14(11.77 to 17.29) | -0.15(-0.40 to 0.10) |
| **Abbreviations:** IBD, inflammatory bowel disease; AS, age-standardized; WCBA, women of childbearing age; EAPC, estimated annual percentage change; CI, Confidence Interval. | | | | | | |
