# Supplementary material for: Global disease burden of inflammatory bowel disease in women and women of childbearing age from 1990 to 2021 and its prediction to 2040
Source: PLoS One. 2025 Sep 10;20(9):e0331034. doi: 10.1371/journal.pone.0331034 (PMC12422439; doi:10.1371/journal.pone.0331034)
Supplement: S5 Table — Abbreviations: IBD, inflammatory bowel disease; AS, age-standardized; WCBA, women of childbearing age; DALYs, disability-adjusted life-years; EAPC, estimated annual percentage change; CI, Confidence Interval. (DOCX) [file pone.0331034.s008.docx]

Table S5 The DALYs rate for IBD among women and WCBA and its temporal trends from 1990 to 2021 across 204 countries and regions

| Location | DALYs rate of WCBA _1990 | DALYs rate of WCBA _2021 | EAPC_CI | AS DALYs rate of women _1990 | AS DALYs rate of women _2021 | EAPC_CI |
| --- | --- | --- | --- | --- | --- | --- |
| Afghanistan | 10.07(3.79 to 31.35) | 11.23(4.74 to 30.63) | 0.60(0.48 to 0.73) | 14.16(6.31 to 39.85) | 14.72(6.75 to 34.38) | 0.37(0.21 to 0.52) |
| Albania | 25.54(18.53 to 34.89) | 21.38(14.81 to 31.18) | -0.51(-0.63 to -0.38) | 35.90(26.69 to 47.93) | 23.74(16.79 to 33.41) | -1.18(-1.34 to -1.02) |
| Algeria | 8.52(5.09 to 15.00) | 10.73(6.68 to 15.92) | 0.92(0.80 to 1.05) | 10.02(6.08 to 17.82) | 10.11(6.57 to 15.79) | 0.34(0.18 to 0.50) |
| American Samoa | 2.64(1.81 to 4.14) | 4.04(2.73 to 5.77) | 2.10(1.57 to 2.65) | 3.66(2.60 to 5.53) | 4.46(3.09 to 6.25) | 1.44(0.89 to 2.00) |
| Andorra | 31.41(20.58 to 45.41) | 35.82(24.40 to 49.93) | 0.57(0.49 to 0.66) | 34.77(25.53 to 47.47) | 31.93(22.87 to 43.22) | -0.14(-0.19 to -0.08) |
| Angola | 7.51(3.01 to 13.88) | 7.35(4.11 to 11.44) | 0.13(-0.03 to 0.29) | 9.90(3.90 to 18.14) | 9.04(5.17 to 13.77) | -0.23(-0.31 to -0.14) |
| Antigua and Barbuda | 17.04(14.09 to 20.96) | 11.86(8.62 to 16.05) | -1.05(-1.21 to -0.88) | 20.07(17.41 to 23.41) | 12.22(9.94 to 15.04) | -1.79(-2.04 to -1.55) |
| Argentina | 14.17(9.99 to 18.81) | 13.73(9.36 to 19.16) | -0.10(-0.22 to 0.02) | 14.70(11.66 to 18.47) | 13.25(10.01 to 17.15) | -0.26(-0.37 to -0.14) |
| Armenia | 14.72(10.94 to 18.71) | 14.22(10.26 to 19.91) | -0.14(-0.31 to 0.04) | 17.09(13.31 to 21.25) | 15.00(11.81 to 19.03) | -0.29(-0.52 to -0.05) |
| Australia | 37.40(23.84 to 53.81) | 44.44(29.81 to 62.21) | 1.25(0.72 to 1.78) | 35.45(25.17 to 48.11) | 45.85(34.48 to 59.16) | 1.35(0.98 to 1.73) |
| Austria | 27.49(19.11 to 38.13) | 29.75(19.95 to 41.94) | 0.03(-0.20 to 0.26) | 30.24(23.92 to 38.42) | 28.61(21.79 to 37.44) | -0.03(-0.29 to 0.22) |
| Azerbaijan | 9.68(6.70 to 13.40) | 11.01(7.33 to 15.71) | 0.35(0.29 to 0.40) | 10.99(7.84 to 14.89) | 10.20(7.00 to 13.91) | -0.18(-0.29 to -0.07) |
| Bahamas | 26.77(22.26 to 31.69) | 19.18(14.96 to 24.37) | -1.26(-1.51 to -1.01) | 30.21(25.64 to 34.88) | 17.75(14.51 to 21.72) | -1.70(-1.97 to -1.44) |
| Bahrain | 8.87(5.50 to 13.76) | 9.53(5.86 to 13.67) | 0.47(0.34 to 0.60) | 10.73(6.48 to 19.07) | 9.72(6.15 to 15.22) | -0.29(-0.37 to -0.22) |
| Bangladesh | 15.21(9.45 to 22.50) | 16.15(11.35 to 22.17) | 0.39(0.26 to 0.52) | 21.51(14.83 to 29.35) | 18.00(13.24 to 24.45) | -0.48(-0.51 to -0.44) |
| Barbados | 19.26(16.18 to 22.98) | 19.10(14.91 to 24.28) | -0.08(-0.41 to 0.25) | 21.51(18.65 to 24.32) | 19.56(15.85 to 24.03) | -0.07(-0.40 to 0.26) |
| Belarus | 12.21(9.27 to 16.43) | 14.79(10.31 to 19.96) | 0.45(0.35 to 0.55) | 14.86(12.27 to 18.63) | 14.33(11.12 to 18.41) | -0.09(-0.21 to 0.03) |
| Belgium | 27.20(20.42 to 35.37) | 30.26(21.06 to 41.13) | 0.17(-0.01 to 0.35) | 31.31(26.31 to 37.01) | 31.80(25.09 to 39.24) | 0.04(-0.41 to 0.50) |
| Belize | 13.41(10.84 to 17.42) | 11.46(9.04 to 14.21) | -0.78(-1.10 to -0.46) | 19.17(16.25 to 22.83) | 12.61(10.39 to 14.91) | -1.61(-1.88 to -1.33) |
| Benin | 38.51(19.32 to 63.98) | 52.55(23.72 to 94.28) | 0.78(0.59 to 0.96) | 23.68(12.50 to 37.65) | 30.59(14.76 to 50.88) | 0.68(0.52 to 0.83) |
| Bermuda | 24.81(20.13 to 29.12) | 12.47(9.02 to 17.16) | -2.57(-2.87 to -2.27) | 31.33(25.79 to 35.36) | 11.03(8.49 to 14.20) | -3.75(-4.18 to -3.31) |
| Bhutan | 14.86(8.22 to 25.48) | 16.44(11.03 to 23.59) | 0.15(0.07 to 0.24) | 18.79(11.62 to 28.86) | 17.81(12.74 to 24.51) | -0.21(-0.27 to -0.16) |
| Bolivia (Plurinational State of) | 6.72(3.44 to 11.19) | 5.38(3.55 to 8.12) | -0.95(-1.03 to -0.86) | 11.20(5.10 to 21.37) | 7.64(5.25 to 11.41) | -1.40(-1.47 to -1.33) |
| Bosnia and Herzegovina | 18.82(14.03 to 25.98) | 17.51(12.14 to 24.60) | -0.34(-0.47 to -0.21) | 23.02(16.85 to 32.37) | 17.25(12.18 to 23.43) | -0.90(-1.01 to -0.79) |
| Botswana | 8.42(4.67 to 14.19) | 7.43(4.49 to 11.51) | 0.11(-0.37 to 0.59) | 14.07(8.93 to 20.46) | 10.99(7.51 to 15.42) | -0.37(-0.76 to 0.01) |
| Brazil | 12.65(11.74 to 13.93) | 16.14(14.36 to 18.28) | 0.75(0.51 to 0.99) | 15.56(14.67 to 16.72) | 17.29(15.70 to 18.99) | 0.39(0.15 to 0.63) |
| Brunei Darussalam | 16.45(10.17 to 27.04) | 12.27(8.78 to 18.97) | -0.44(-0.65 to -0.23) | 27.70(17.77 to 41.27) | 19.81(14.18 to 28.63) | -0.50(-0.71 to -0.28) |
| Bulgaria | 15.97(11.35 to 23.27) | 18.27(12.45 to 25.85) | 0.62(0.51 to 0.72) | 13.75(10.36 to 18.25) | 14.06(10.29 to 18.44) | 0.26(0.12 to 0.39) |
| Burkina Faso | 37.75(22.64 to 57.42) | 46.00(22.80 to 76.15) | 0.82(0.54 to 1.11) | 23.43(14.57 to 35.09) | 27.70(14.21 to 44.43) | 0.70(0.45 to 0.96) |
| Burundi | 7.69(3.86 to 12.50) | 6.98(4.20 to 10.58) | -0.66(-0.84 to -0.49) | 11.34(6.13 to 17.14) | 9.97(6.44 to 15.17) | -0.66(-0.76 to -0.56) |
| Cabo Verde | 51.97(29.59 to 80.02) | 31.47(17.67 to 51.39) | -1.93(-2.05 to -1.82) | 30.85(18.57 to 45.40) | 17.93(10.89 to 28.46) | -1.90(-1.98 to -1.82) |
| Cambodia | 7.47(2.67 to 14.08) | 5.99(3.20 to 10.50) | -0.90(-1.00 to -0.80) | 14.34(5.47 to 25.02) | 10.02(5.14 to 16.02) | -1.36(-1.46 to -1.25) |
| Cameroon | 44.03(24.20 to 70.04) | 49.85(23.54 to 85.75) | 0.37(0.28 to 0.47) | 26.95(15.18 to 41.73) | 28.43(14.08 to 47.79) | 0.13(0.04 to 0.22) |
| Canada | 105.80(73.40 to 143.15) | 76.20(49.62 to 109.55) | -1.11(-1.51 to -0.70) | 80.62(59.94 to 104.02) | 64.55(45.86 to 87.53) | -0.68(-1.02 to -0.34) |
| Central African Republic | 6.67(3.05 to 11.79) | 7.98(4.16 to 13.03) | 0.57(0.48 to 0.65) | 8.83(4.29 to 14.92) | 10.47(6.22 to 16.77) | 0.59(0.47 to 0.71) |
| Chad | 39.93(18.58 to 75.43) | 53.77(25.60 to 93.43) | 1.11(0.99 to 1.23) | 24.92(12.09 to 45.37) | 33.12(16.73 to 56.63) | 1.05(0.93 to 1.17) |
| Chile | 14.55(11.28 to 18.90) | 13.19(9.51 to 18.16) | -0.29(-0.44 to -0.13) | 18.55(15.76 to 22.15) | 14.02(11.09 to 17.84) | -0.85(-1.06 to -0.64) |
| China | 5.45(2.98 to 7.90) | 3.73(2.62 to 5.10) | -0.83(-1.15 to -0.52) | 19.18(9.09 to 27.73) | 6.23(4.38 to 9.44) | -3.77(-4.07 to -3.48) |
| Colombia | 6.29(5.44 to 7.27) | 6.31(5.14 to 7.77) | 0.41(0.23 to 0.58) | 9.37(8.50 to 10.53) | 7.80(6.59 to 9.21) | -0.08(-0.28 to 0.12) |
| Comoros | 9.47(5.24 to 15.16) | 9.62(6.63 to 13.56) | -0.41(-0.78 to -0.04) | 13.19(8.40 to 19.42) | 12.38(8.65 to 17.44) | -0.44(-0.58 to -0.29) |
| Congo | 6.85(3.34 to 11.94) | 8.97(5.43 to 15.15) | 1.02(0.87 to 1.18) | 9.48(4.64 to 16.23) | 10.48(6.80 to 16.64) | 0.39(0.23 to 0.54) |
| Cook Islands | 2.43(1.71 to 3.35) | 2.37(1.56 to 3.64) | 0.12(0.03 to 0.22) | 2.64(1.92 to 3.53) | 2.25(1.47 to 3.40) | -0.69(-0.80 to -0.59) |
| Costa Rica | 3.16(2.62 to 3.82) | 3.93(3.30 to 4.71) | 0.57(0.30 to 0.83) | 4.54(4.04 to 5.09) | 4.81(4.19 to 5.45) | 0.09(-0.13 to 0.31) |
| Côte d'Ivoire | 35.63(19.47 to 60.07) | 54.63(24.40 to 94.90) | 0.04(-0.58 to 0.67) | 21.86(12.41 to 36.33) | 30.39(14.62 to 51.55) | 1.41(1.27 to 1.56) |
| Croatia | 19.63(13.96 to 27.72) | 21.36(14.49 to 30.25) | -1.33(-1.48 to -1.18) | 18.69(14.68 to 24.54) | 19.11(14.62 to 24.85) | 0.08(-0.34 to 0.50) |
| Cuba | 14.68(11.74 to 18.01) | 10.21(7.53 to 13.72) | -0.25(-0.34 to -0.17) | 16.56(13.96 to 19.76) | 9.46(7.48 to 11.89) | -1.95(-2.18 to -1.73) |
| Cyprus | 31.77(21.54 to 43.98) | 30.36(21.04 to 42.71) | 1.07(0.61 to 1.53) | 64.25(41.85 to 94.80) | 37.51(28.39 to 48.09) | -1.88(-2.08 to -1.67) |
| Czechia | 48.82(34.74 to 67.62) | 54.82(37.20 to 76.47) | 1.80(1.64 to 1.96) | 43.75(33.40 to 56.67) | 43.15(32.41 to 56.64) | 0.47(0.09 to 0.86) |
| Democratic People's Republic of Korea | 5.29(2.77 to 9.17) | 4.85(2.77 to 8.04) | -0.23(-0.28 to -0.17) | 13.41(6.88 to 22.15) | 10.23(6.28 to 16.54) | -0.80(-0.89 to -0.72) |
| Democratic Republic of the Congo | 7.49(4.37 to 11.76) | 6.94(4.46 to 10.54) | -0.40(-0.60 to -0.20) | 9.54(5.86 to 14.23) | 8.48(5.79 to 12.60) | -0.39(-0.50 to -0.27) |
| Denmark | 45.64(31.32 to 62.16) | 39.57(26.10 to 55.02) | -0.53(-0.95 to -0.10) | 38.59(28.96 to 50.48) | 36.97(27.13 to 48.34) | -0.05(-0.44 to 0.34) |
| Djibouti | 7.24(4.48 to 11.22) | 7.44(4.76 to 10.94) | -0.03(-0.21 to 0.14) | 11.68(7.95 to 16.70) | 9.98(6.68 to 14.89) | -0.58(-0.71 to -0.46) |
| Dominica | 9.82(7.09 to 12.72) | 10.87(7.71 to 14.75) | 0.36(0.28 to 0.44) | 12.35(9.14 to 15.88) | 11.13(8.18 to 15.03) | -0.34(-0.44 to -0.25) |
| Dominican Republic | 9.09(6.94 to 12.03) | 9.18(6.58 to 12.68) | -0.03(-0.17 to 0.11) | 11.08(8.50 to 14.74) | 8.77(6.40 to 12.04) | -0.77(-0.90 to -0.64) |
| Ecuador | 6.20(5.37 to 7.25) | 3.83(2.84 to 4.97) | -1.55(-1.79 to -1.30) | 9.25(8.27 to 10.38) | 4.77(3.85 to 5.99) | -2.06(-2.40 to -1.73) |
| Egypt | 12.39(8.82 to 16.47) | 12.55(8.74 to 17.66) | -0.10(-0.25 to 0.05) | 17.04(12.54 to 22.72) | 16.53(11.75 to 23.96) | -0.25(-0.39 to -0.12) |
| El Salvador | 4.54(3.23 to 6.17) | 3.96(2.72 to 5.44) | -0.30(-0.44 to -0.16) | 7.55(5.42 to 10.30) | 5.35(3.57 to 7.30) | -0.82(-1.06 to -0.58) |
| Equatorial Guinea | 9.40(4.84 to 16.56) | 8.34(4.39 to 13.65) | -0.59(-0.80 to -0.37) | 11.66(6.53 to 18.75) | 9.95(5.59 to 16.35) | -0.66(-0.89 to -0.43) |
| Eritrea | 8.85(4.62 to 14.91) | 9.60(6.25 to 14.33) | 0.16(0.06 to 0.26) | 13.51(7.76 to 21.10) | 13.60(8.85 to 19.54) | -0.07(-0.17 to 0.02) |
| Estonia | 23.50(19.53 to 28.90) | 12.42(8.54 to 16.99) | -2.75(-3.64 to -1.84) | 34.29(30.20 to 39.31) | 11.67(8.95 to 14.81) | -4.06(-5.19 to -2.91) |
| Eswatini | 7.67(4.62 to 12.52) | 9.10(5.06 to 14.31) | 0.72(0.41 to 1.02) | 13.20(8.21 to 20.73) | 13.81(8.45 to 20.96) | 0.35(0.10 to 0.60) |
| Ethiopia | 6.56(3.65 to 11.89) | 5.75(3.82 to 8.62) | -0.81(-0.98 to -0.64) | 10.54(6.82 to 18.18) | 8.78(5.88 to 12.97) | -0.93(-1.08 to -0.77) |
| Fiji | 2.85(1.97 to 3.99) | 2.59(1.78 to 3.80) | -0.22(-0.34 to -0.11) | 3.33(2.27 to 4.65) | 2.80(1.96 to 3.91) | -0.46(-0.60 to -0.31) |
| Finland | 39.25(26.80 to 54.45) | 34.05(23.04 to 48.80) | -1.28(-1.83 to -0.74) | 31.03(22.82 to 40.62) | 31.10(22.34 to 42.05) | -0.48(-0.93 to -0.02) |
| France | 35.60(27.27 to 47.01) | 33.69(23.40 to 45.21) | -0.28(-0.54 to -0.01) | 36.69(30.97 to 43.47) | 41.64(34.46 to 50.55) | 0.65(0.40 to 0.89) |
| Gabon | 6.34(3.39 to 10.24) | 7.30(4.24 to 11.38) | 0.35(0.16 to 0.54) | 8.45(4.37 to 12.91) | 8.95(5.57 to 13.11) | 0.18(0.01 to 0.36) |
| Gambia | 49.37(24.56 to 88.30) | 76.75(39.06 to 131.36) | 1.25(1.00 to 1.51) | 31.09(16.86 to 53.42) | 43.97(23.67 to 72.46) | 0.92(0.68 to 1.16) |
| Georgia | 13.07(9.06 to 17.66) | 14.80(10.53 to 19.89) | -0.03(-0.51 to 0.46) | 14.01(10.24 to 18.35) | 14.31(11.11 to 17.98) | -0.32(-0.84 to 0.21) |
| Germany | 60.60(41.69 to 83.22) | 66.77(46.63 to 91.61) | 0.56(0.15 to 0.97) | 52.30(40.43 to 67.09) | 72.16(57.79 to 89.82) | 1.58(1.21 to 1.95) |
| Ghana | 44.42(25.30 to 67.76) | 43.54(20.77 to 80.20) | -0.17(-0.26 to -0.09) | 26.03(15.26 to 38.14) | 24.75(12.74 to 43.50) | -0.23(-0.30 to -0.15) |
| Greece | 7.56(5.15 to 10.59) | 10.08(6.72 to 14.03) | 1.16(0.60 to 1.73) | 8.35(6.62 to 10.48) | 10.31(8.13 to 12.90) | 0.86(0.47 to 1.26) |
| Greenland | 49.02(32.24 to 67.86) | 46.03(30.52 to 63.56) | -0.36(-0.45 to -0.27) | 55.54(42.33 to 70.33) | 51.20(35.28 to 68.22) | -0.05(-0.16 to 0.06) |
| Grenada | 33.71(27.06 to 40.19) | 21.09(17.32 to 25.81) | -1.62(-1.85 to -1.39) | 39.91(31.50 to 47.10) | 24.32(21.01 to 28.13) | -1.62(-1.84 to -1.40) |
| Guam | 1.93(1.19 to 2.87) | 2.39(1.65 to 3.31) | 1.24(0.80 to 1.69) | 2.33(1.49 to 3.85) | 2.29(1.72 to 3.04) | 0.97(0.34 to 1.60) |
| Guatemala | 7.26(5.52 to 8.35) | 5.63(4.72 to 6.69) | -0.72(-1.24 to -0.19) | 10.22(7.70 to 11.52) | 7.34(6.35 to 8.41) | -0.90(-1.36 to -0.43) |
| Guinea | 37.73(16.47 to 71.78) | 52.69(27.07 to 85.48) | 1.07(0.96 to 1.17) | 22.49(10.01 to 41.54) | 30.11(16.15 to 48.23) | 0.92(0.78 to 1.07) |
| Guinea-Bissau | 62.77(26.19 to 118.77) | 87.33(47.06 to 141.34) | 1.19(1.07 to 1.31) | 37.70(16.41 to 71.96) | 48.25(27.13 to 77.13) | 0.89(0.72 to 1.06) |
| Guyana | 25.39(19.31 to 33.44) | 27.97(20.61 to 36.45) | -0.03(-0.35 to 0.30) | 36.85(30.90 to 42.76) | 30.32(23.40 to 38.23) | -0.52(-0.84 to -0.19) |
| Haiti | 28.45(11.14 to 52.44) | 25.59(12.96 to 46.11) | -0.20(-0.32 to -0.07) | 38.26(14.78 to 70.97) | 31.88(15.48 to 55.01) | -0.45(-0.50 to -0.40) |
| Honduras | 11.87(5.04 to 19.65) | 8.56(4.17 to 15.19) | -1.18(-1.28 to -1.07) | 16.35(7.87 to 23.27) | 11.92(6.89 to 19.84) | -1.02(-1.09 to -0.95) |
| Hungary | 47.43(35.75 to 62.65) | 47.06(32.95 to 64.32) | -0.11(-0.43 to 0.20) | 40.61(32.31 to 50.54) | 40.08(31.05 to 51.03) | -0.11(-0.43 to 0.22) |
| Iceland | 50.90(36.40 to 67.73) | 44.53(31.08 to 61.26) | -0.73(-0.90 to -0.55) | 49.16(38.49 to 61.87) | 45.85(35.99 to 58.41) | -0.22(-0.41 to -0.02) |
| India | 14.55(8.65 to 22.39) | 12.72(9.05 to 17.71) | -0.40(-0.57 to -0.23) | 18.02(11.84 to 26.03) | 15.16(10.95 to 21.19) | -0.50(-0.60 to -0.40) |
| Indonesia | 5.96(2.47 to 9.17) | 3.85(2.50 to 5.20) | -1.66(-1.79 to -1.53) | 13.91(5.50 to 22.66) | 8.07(4.97 to 10.94) | -2.12(-2.29 to -1.94) |
| Iran (Islamic Republic of) | 6.63(4.72 to 9.00) | 7.64(5.36 to 10.92) | 1.06(0.76 to 1.35) | 8.66(6.27 to 11.31) | 7.99(5.55 to 10.93) | 0.30(0.08 to 0.52) |
| Iraq | 12.89(7.77 to 18.61) | 11.30(8.07 to 16.10) | -0.41(-0.52 to -0.30) | 15.20(9.67 to 21.21) | 12.27(8.77 to 17.14) | -0.67(-0.78 to -0.56) |
| Ireland | 34.41(23.69 to 48.12) | 39.48(26.78 to 54.64) | 0.81(0.59 to 1.02) | 39.78(31.79 to 49.53) | 38.11(29.14 to 48.80) | 0.20(-0.12 to 0.53) |
| Israel | 25.01(16.39 to 35.46) | 27.38(17.25 to 39.99) | -0.37(-1.06 to 0.33) | 25.09(18.44 to 33.18) | 25.83(18.59 to 34.69) | -0.34(-0.86 to 0.18) |
| Italy | 23.26(16.51 to 30.76) | 21.26(15.64 to 28.52) | 0.15(-0.10 to 0.40) | 24.56(19.24 to 30.67) | 27.46(22.85 to 32.91) | 0.78(0.56 to 1.01) |
| Jamaica | 8.83(6.79 to 11.76) | 11.56(8.80 to 15.25) | 0.67(0.35 to 0.98) | 10.81(8.97 to 13.41) | 11.46(9.10 to 14.59) | 0.17(-0.07 to 0.42) |
| Japan | 7.47(5.68 to 9.82) | 6.42(4.37 to 9.00) | -0.59(-1.01 to -0.17) | 8.18(6.77 to 9.96) | 5.65(4.15 to 7.57) | -1.15(-1.46 to -0.83) |
| Jordan | 11.53(7.63 to 17.74) | 11.58(7.99 to 15.87) | 0.30(0.19 to 0.41) | 16.26(10.81 to 25.89) | 14.22(10.29 to 18.79) | -0.16(-0.37 to 0.05) |
| Kazakhstan | 19.54(15.45 to 24.49) | 24.27(19.17 to 30.76) | 0.16(-0.11 to 0.44) | 23.11(19.20 to 27.27) | 22.71(18.39 to 27.96) | -0.50(-0.73 to -0.28) |
| Kenya | 6.79(3.61 to 11.48) | 7.42(4.52 to 11.00) | 0.46(0.38 to 0.54) | 11.31(6.44 to 18.69) | 11.09(6.93 to 16.33) | 0.21(0.11 to 0.31) |
| Kiribati | 2.36(1.53 to 3.73) | 2.57(1.68 to 3.73) | 0.31(0.21 to 0.41) | 3.15(1.87 to 4.94) | 3.23(2.01 to 4.83) | 0.15(0.07 to 0.23) |
| Kuwait | 11.57(7.62 to 16.78) | 12.55(8.46 to 18.24) | 0.06(-0.61 to 0.73) | 10.07(7.00 to 14.23) | 11.27(8.50 to 14.51) | 0.41(-0.38 to 1.21) |
| Kyrgyzstan | 20.63(16.55 to 25.78) | 12.56(9.40 to 17.44) | -2.53(-3.08 to -1.97) | 25.72(20.87 to 31.85) | 13.13(10.15 to 17.19) | -3.05(-3.64 to -2.46) |
| Lao People's Democratic Republic | 3.64(1.47 to 6.17) | 3.36(1.96 to 5.16) | -0.32(-0.38 to -0.25) | 7.46(2.44 to 12.92) | 5.12(2.69 to 8.29) | -1.30(-1.37 to -1.23) |
| Latvia | 26.34(21.69 to 31.10) | 13.80(9.74 to 18.87) | -2.73(-3.73 to -1.72) | 35.09(30.60 to 40.36) | 14.06(11.13 to 17.50) | -3.63(-4.84 to -2.42) |
| Lebanon | 21.03(13.47 to 30.86) | 22.36(15.46 to 31.35) | 0.11(0.04 to 0.18) | 23.46(15.89 to 32.98) | 20.60(15.33 to 26.95) | -0.31(-0.36 to -0.25) |
| Lesotho | 6.43(3.48 to 12.70) | 8.83(5.71 to 13.52) | 1.70(1.30 to 2.11) | 11.29(6.58 to 20.12) | 14.24(9.39 to 20.94) | 1.33(0.99 to 1.68) |
| Liberia | 34.11(18.06 to 53.96) | 48.27(24.35 to 89.09) | 1.20(1.05 to 1.35) | 21.31(11.92 to 33.04) | 27.96(14.71 to 49.75) | 0.82(0.68 to 0.95) |
| Libya | 6.31(3.86 to 10.80) | 11.31(7.03 to 18.45) | 2.48(2.21 to 2.75) | 8.41(5.21 to 14.29) | 11.94(7.39 to 22.56) | 1.68(1.43 to 1.93) |
| Lithuania | 19.75(15.68 to 24.78) | 14.08(9.40 to 20.09) | -1.02(-1.69 to -0.35) | 25.33(21.37 to 29.89) | 13.47(9.95 to 17.53) | -2.16(-3.02 to -1.30) |
| Luxembourg | 33.31(23.91 to 46.18) | 34.91(23.74 to 48.30) | 0.17(-0.01 to 0.36) | 37.22(30.28 to 45.54) | 39.30(30.91 to 49.24) | 0.54(0.27 to 0.81) |
| Madagascar | 8.36(4.41 to 13.62) | 9.34(5.76 to 15.43) | 0.21(0.09 to 0.32) | 12.16(7.35 to 17.67) | 12.06(7.54 to 19.21) | -0.09(-0.14 to -0.04) |
| Malawi | 7.91(4.45 to 12.11) | 7.72(4.67 to 11.53) | 0.01(-0.09 to 0.11) | 11.55(7.69 to 16.74) | 10.96(7.08 to 16.18) | -0.14(-0.26 to -0.02) |
| Malaysia | 1.96(1.41 to 2.63) | 2.73(1.85 to 3.77) | 1.56(1.21 to 1.91) | 2.32(1.74 to 3.01) | 2.60(1.91 to 3.41) | 0.61(0.33 to 0.90) |
| Maldives | 3.12(1.69 to 5.70) | 2.69(1.89 to 3.86) | -0.47(-0.62 to -0.32) | 6.55(3.18 to 10.61) | 3.69(2.69 to 4.89) | -2.01(-2.17 to -1.85) |
| Mali | 66.26(33.88 to 115.27) | 79.78(43.18 to 124.49) | 0.67(0.59 to 0.75) | 38.91(20.01 to 68.33) | 46.73(26.67 to 72.50) | 0.67(0.58 to 0.77) |
| Malta | 23.31(16.22 to 32.14) | 26.01(18.19 to 35.77) | 0.43(0.34 to 0.51) | 24.34(19.47 to 30.71) | 26.66(21.08 to 33.58) | 0.47(0.25 to 0.70) |
| Marshall Islands | 2.21(1.45 to 3.12) | 2.46(1.62 to 3.83) | 0.30(0.19 to 0.41) | 3.32(2.05 to 4.81) | 2.82(1.89 to 4.26) | -0.59(-0.70 to -0.48) |
| Mauritania | 31.25(13.32 to 59.79) | 41.61(22.83 to 70.03) | 0.81(0.58 to 1.05) | 19.71(9.04 to 36.98) | 25.33(14.86 to 41.99) | 0.71(0.51 to 0.91) |
| Mauritius | 2.78(2.24 to 3.51) | 7.37(6.41 to 8.46) | 2.48(1.21 to 3.78) | 3.82(3.40 to 4.37) | 10.33(9.40 to 11.29) | 2.28(0.76 to 3.83) |
| Mexico | 5.46(5.26 to 5.70) | 7.15(6.02 to 8.30) | 1.68(1.37 to 1.99) | 10.91(10.58 to 11.19) | 11.21(9.62 to 12.86) | 0.91(0.58 to 1.23) |
| Micronesia (Federated States of) | 2.64(1.69 to 3.88) | 2.66(1.73 to 3.98) | -0.02(-0.08 to 0.03) | 3.70(2.32 to 5.54) | 3.05(1.99 to 4.32) | -0.64(-0.66 to -0.61) |
| Monaco | 32.67(21.39 to 47.63) | 33.28(22.08 to 47.42) | 0.08(0.01 to 0.14) | 29.52(21.12 to 40.42) | 29.84(21.90 to 40.18) | 0.08(0.06 to 0.11) |
| Mongolia | 16.08(9.77 to 26.10) | 14.38(9.76 to 20.65) | -0.75(-0.88 to -0.63) | 25.47(14.66 to 42.78) | 14.41(10.01 to 20.34) | -2.29(-2.45 to -2.13) |
| Montenegro | 21.00(15.77 to 28.19) | 21.18(14.98 to 29.13) | 0.18(0.07 to 0.28) | 20.44(15.75 to 26.33) | 19.46(14.28 to 26.25) | 0.02(-0.06 to 0.10) |
| Morocco | 8.36(4.90 to 15.43) | 9.97(6.18 to 15.62) | 0.72(0.62 to 0.83) | 9.67(5.80 to 18.26) | 10.01(6.53 to 16.54) | 0.41(0.26 to 0.57) |
| Mozambique | 6.43(4.15 to 9.43) | 6.94(4.28 to 11.27) | 0.37(0.31 to 0.43) | 10.34(6.92 to 15.16) | 11.07(6.80 to 17.27) | 0.43(0.37 to 0.49) |
| Myanmar | 5.27(2.12 to 9.00) | 3.42(2.20 to 5.10) | -1.65(-1.79 to -1.51) | 7.22(2.66 to 12.26) | 4.07(2.57 to 6.03) | -2.17(-2.32 to -2.02) |
| Namibia | 9.61(5.27 to 15.51) | 8.26(4.57 to 14.00) | -0.67(-0.79 to -0.55) | 16.05(10.22 to 24.25) | 13.58(8.50 to 21.40) | -0.59(-0.66 to -0.53) |
| Nauru | 2.73(1.69 to 4.20) | 2.54(1.62 to 3.89) | -0.32(-0.47 to -0.17) | 3.56(2.23 to 5.31) | 3.10(1.91 to 4.78) | -0.48(-0.59 to -0.37) |
| Nepal | 18.94(10.05 to 32.02) | 15.47(10.61 to 21.83) | -0.91(-1.01 to -0.81) | 22.26(12.47 to 34.76) | 17.21(12.37 to 23.39) | -0.96(-1.06 to -0.86) |
| Netherlands | 63.32(47.26 to 83.25) | 67.64(48.54 to 90.45) | 0.18(-0.53 to 0.90) | 68.01(55.92 to 82.03) | 78.73(63.52 to 96.62) | 0.57(0.03 to 1.11) |
| New Zealand | 35.95(24.19 to 51.83) | 35.09(22.77 to 49.52) | 0.03(-0.30 to 0.36) | 39.69(29.18 to 52.72) | 36.37(26.04 to 48.55) | -0.19(-0.50 to 0.13) |
| Nicaragua | 3.19(2.29 to 4.32) | 2.90(1.96 to 3.90) | -0.03(-0.18 to 0.12) | 4.65(3.46 to 6.28) | 3.81(2.57 to 5.06) | -0.24(-0.38 to -0.09) |
| Niger | 39.72(17.37 to 72.59) | 47.76(21.39 to 96.14) | 0.38(0.21 to 0.55) | 24.81(11.35 to 43.16) | 29.41(14.15 to 56.05) | 0.32(0.17 to 0.47) |
| Nigeria | 28.90(14.69 to 47.55) | 34.37(13.63 to 62.80) | 0.59(0.49 to 0.70) | 19.47(10.62 to 30.46) | 22.22(10.39 to 37.70) | 0.50(0.42 to 0.58) |
| Niue | 2.91(1.91 to 4.30) | 3.04(1.95 to 4.63) | -0.07(-0.15 to 0.02) | 3.34(2.29 to 4.79) | 5.39(2.81 to 10.60) | 0.13(-0.43 to 0.68) |
| North Macedonia | 14.99(10.14 to 20.43) | 16.42(10.73 to 23.40) | 0.26(0.22 to 0.30) | 13.37(9.75 to 17.65) | 13.34(9.71 to 18.01) | 0.08(0.02 to 0.13) |
| Northern Mariana Islands | 1.76(1.14 to 2.62) | 1.86(1.21 to 2.66) | 0.11(-0.12 to 0.34) | 1.79(1.27 to 2.54) | 1.57(1.08 to 2.17) | -0.45(-0.55 to -0.35) |
| Norway | 39.73(27.16 to 54.98) | 43.32(29.58 to 60.44) | -0.06(-0.26 to 0.14) | 38.55(28.11 to 51.50) | 42.62(31.14 to 56.40) | -0.04(-0.23 to 0.15) |
| Oman | 8.63(5.44 to 13.78) | 10.16(6.46 to 14.94) | 0.81(0.57 to 1.05) | 9.69(6.08 to 17.48) | 9.41(6.17 to 13.72) | 0.26(0.09 to 0.42) |
| Pakistan | 16.38(9.04 to 29.31) | 17.06(10.97 to 26.26) | -0.17(-0.29 to -0.06) | 22.84(12.76 to 41.17) | 21.19(14.62 to 29.57) | -0.51(-0.60 to -0.41) |
| Palau | 2.65(1.77 to 3.73) | 3.06(2.00 to 4.38) | 0.38(0.31 to 0.45) | 3.55(2.41 to 4.82) | 3.36(2.21 to 4.80) | -0.11(-0.14 to -0.09) |
| Palestine | 17.59(10.96 to 26.60) | 11.48(8.11 to 15.32) | -1.34(-1.64 to -1.04) | 31.92(18.41 to 48.40) | 16.35(12.55 to 21.99) | -2.06(-2.26 to -1.85) |
| Panama | 10.68(9.30 to 12.06) | 11.64(9.36 to 14.08) | 0.78(0.52 to 1.05) | 14.98(13.39 to 16.72) | 15.27(12.36 to 18.06) | 0.52(0.29 to 0.74) |
| Papua New Guinea | 1.94(1.30 to 2.81) | 1.87(1.27 to 2.65) | -0.36(-0.53 to -0.20) | 2.36(1.54 to 3.44) | 1.95(1.41 to 2.67) | -0.91(-1.08 to -0.74) |
| Paraguay | 6.10(4.43 to 7.99) | 5.64(4.08 to 7.71) | 0.06(-0.12 to 0.24) | 6.60(5.03 to 8.61) | 6.22(4.44 to 8.33) | 0.28(0.12 to 0.44) |
| Peru | 7.68(5.15 to 10.56) | 4.86(3.45 to 6.79) | -1.87(-2.26 to -1.47) | 14.15(7.57 to 22.52) | 5.78(4.16 to 8.19) | -3.25(-3.75 to -2.74) |
| Philippines | 3.24(2.02 to 4.61) | 2.60(1.90 to 3.70) | -1.00(-1.19 to -0.82) | 6.29(3.31 to 9.28) | 4.22(2.98 to 5.95) | -1.41(-1.62 to -1.21) |
| Poland | 12.75(10.70 to 15.35) | 12.07(9.63 to 15.16) | -0.06(-0.25 to 0.13) | 15.54(13.99 to 17.59) | 14.09(12.27 to 16.28) | 0.06(-0.27 to 0.39) |
| Portugal | 21.68(16.45 to 28.96) | 23.65(16.97 to 32.71) | 0.61(0.25 to 0.99) | 26.87(22.80 to 31.99) | 27.74(22.97 to 33.09) | 0.67(0.31 to 1.04) |
| Puerto Rico | 18.98(16.04 to 22.27) | 12.57(9.69 to 16.25) | -2.00(-2.25 to -1.74) | 23.76(20.92 to 26.72) | 11.99(9.74 to 14.76) | -2.72(-2.98 to -2.47) |
| Qatar | 17.47(11.34 to 25.06) | 14.38(9.33 to 20.45) | -0.68(-0.87 to -0.49) | 32.19(20.65 to 45.05) | 17.81(12.96 to 23.46) | -1.72(-1.89 to -1.54) |
| Republic of Korea | 18.15(10.73 to 24.91) | 13.10(8.28 to 17.90) | -0.23(-0.77 to 0.31) | 28.63(14.27 to 39.86) | 12.23(8.38 to 18.09) | -2.86(-3.00 to -2.73) |
| Republic of Moldova | 16.47(13.05 to 20.31) | 10.69(7.21 to 15.13) | -1.90(-2.71 to -1.07) | 25.55(21.37 to 30.55) | 10.94(8.65 to 13.62) | -3.27(-4.50 to -2.02) |
| Romania | 6.43(5.06 to 8.00) | 6.41(4.82 to 8.52) | 0.53(-0.05 to 1.11) | 6.58(5.36 to 7.86) | 5.99(4.82 to 7.30) | 0.18(-0.27 to 0.63) |
| Russian Federation | 16.66(13.59 to 20.85) | 17.75(14.81 to 21.46) | -0.63(-1.07 to -0.19) | 21.53(18.53 to 25.10) | 19.99(17.56 to 22.83) | -0.83(-1.42 to -0.23) |
| Rwanda | 8.90(4.45 to 15.55) | 8.00(4.66 to 13.12) | -0.56(-0.80 to -0.31) | 12.91(7.24 to 21.18) | 11.49(6.98 to 18.31) | -0.56(-0.71 to -0.42) |
| Saint Kitts and Nevis | 18.46(12.54 to 23.20) | 10.50(7.75 to 14.14) | -2.15(-2.50 to -1.80) | 21.81(16.40 to 26.34) | 10.52(8.40 to 13.11) | -2.44(-2.69 to -2.19) |
| Saint Lucia | 24.96(20.28 to 29.27) | 19.82(15.70 to 24.64) | -0.91(-1.22 to -0.60) | 34.46(28.77 to 39.45) | 19.60(16.00 to 23.35) | -2.30(-2.72 to -1.87) |
| Saint Vincent and the Grenadines | 27.31(23.57 to 32.06) | 25.72(21.08 to 30.55) | -0.49(-0.90 to -0.08) | 36.49(32.20 to 41.38) | 25.74(21.77 to 29.90) | -1.24(-1.58 to -0.89) |
| Samoa | 2.07(1.36 to 3.08) | 2.36(1.61 to 3.40) | 0.50(0.39 to 0.61) | 3.01(1.95 to 4.62) | 2.92(1.97 to 4.04) | -0.10(-0.14 to -0.06) |
| San Marino | 36.33(23.19 to 55.14) | 41.29(26.06 to 61.46) | 0.67(0.50 to 0.84) | 34.62(24.63 to 47.57) | 32.75(22.46 to 47.22) | 0.01(-0.11 to 0.12) |
| Sao Tome and Principe | 30.66(11.87 to 63.75) | 34.95(14.03 to 81.86) | 0.36(-0.05 to 0.76) | 20.79(9.98 to 39.79) | 21.12(9.36 to 46.37) | -0.06(-0.38 to 0.26) |
| Saudi Arabia | 7.86(5.03 to 11.96) | 9.76(6.49 to 14.15) | 0.75(0.55 to 0.96) | 9.14(5.84 to 13.68) | 8.81(6.22 to 12.49) | 0.06(-0.15 to 0.28) |
| Senegal | 52.57(28.70 to 86.82) | 55.06(30.39 to 87.60) | 0.28(0.12 to 0.45) | 32.37(18.08 to 51.92) | 31.68(18.19 to 49.46) | 0.00(-0.15 to 0.16) |
| Serbia | 26.26(19.07 to 36.48) | 23.53(16.41 to 32.30) | -0.22(-0.28 to -0.16) | 28.50(21.78 to 39.47) | 24.29(17.72 to 31.41) | -0.29(-0.41 to -0.17) |
| Seychelles | 3.45(2.21 to 4.64) | 3.21(2.19 to 4.51) | -0.15(-0.26 to -0.05) | 5.21(3.02 to 7.10) | 4.10(2.78 to 5.77) | -0.87(-0.98 to -0.76) |
| Sierra Leone | 30.29(13.54 to 55.24) | 52.60(27.01 to 84.75) | 1.94(1.84 to 2.05) | 18.63(8.90 to 32.46) | 30.18(16.79 to 46.35) | 1.79(1.68 to 1.90) |
| Singapore | 3.65(2.80 to 4.74) | 2.92(1.87 to 4.22) | -0.87(-1.02 to -0.73) | 4.23(3.57 to 4.97) | 2.08(1.41 to 2.90) | -2.35(-2.53 to -2.17) |
| Slovakia | 24.09(17.75 to 32.85) | 24.95(17.82 to 33.92) | 0.36(0.28 to 0.43) | 25.89(19.87 to 36.07) | 23.64(17.17 to 30.52) | 0.02(-0.11 to 0.14) |
| Slovenia | 27.08(19.63 to 36.47) | 27.09(18.05 to 38.48) | -0.02(-0.31 to 0.26) | 26.26(20.74 to 33.03) | 23.00(17.74 to 30.17) | -0.43(-0.75 to -0.12) |
| Solomon Islands | 1.40(0.94 to 1.94) | 1.72(1.18 to 2.37) | 0.75(0.72 to 0.79) | 1.95(1.32 to 2.78) | 2.02(1.39 to 2.72) | 0.11(0.05 to 0.18) |
| Somalia | 7.02(3.35 to 13.13) | 6.24(3.74 to 10.82) | -0.67(-0.81 to -0.53) | 10.19(5.55 to 18.01) | 9.33(5.81 to 15.96) | -0.45(-0.54 to -0.36) |
| South Africa | 9.22(6.05 to 12.14) | 7.81(5.27 to 10.74) | -0.23(-0.96 to 0.51) | 11.92(8.23 to 15.08) | 11.18(7.95 to 14.52) | 0.10(-0.28 to 0.49) |
| South Sudan | 7.27(3.92 to 12.91) | 7.49(4.30 to 12.12) | -0.01(-0.21 to 0.19) | 11.24(6.60 to 17.58) | 11.09(6.72 to 16.60) | -0.20(-0.30 to -0.09) |
| Spain | 23.83(16.41 to 33.39) | 24.99(16.38 to 36.36) | 0.14(-0.53 to 0.82) | 24.18(18.55 to 30.80) | 21.68(15.58 to 28.87) | -0.36(-0.81 to 0.09) |
| Sri Lanka | 2.54(1.84 to 3.45) | 2.25(1.50 to 3.20) | -0.00(-0.31 to 0.30) | 2.84(2.01 to 3.72) | 2.06(1.46 to 2.82) | -0.93(-1.10 to -0.75) |
| Sudan | 7.32(4.30 to 14.08) | 9.49(6.03 to 14.66) | 1.12(0.99 to 1.25) | 8.85(5.32 to 16.91) | 10.69(7.18 to 16.40) | 0.95(0.79 to 1.11) |
| Suriname | 23.40(15.53 to 30.80) | 20.65(14.40 to 29.92) | -0.63(-0.82 to -0.44) | 29.41(19.60 to 36.82) | 20.84(15.12 to 29.55) | -1.20(-1.36 to -1.05) |
| Sweden | 46.09(31.55 to 63.87) | 39.50(25.64 to 55.52) | -0.66(-0.87 to -0.46) | 48.74(37.26 to 61.97) | 39.58(28.16 to 52.18) | -1.08(-1.44 to -0.72) |
| Switzerland | 38.26(25.66 to 55.88) | 35.57(22.64 to 53.55) | -0.25(-0.38 to -0.11) | 34.79(26.64 to 46.83) | 32.64(24.19 to 42.86) | -0.12(-0.36 to 0.12) |
| Syrian Arab Republic | 12.78(8.03 to 19.78) | 12.51(8.10 to 17.71) | 0.07(-0.06 to 0.21) | 17.69(11.70 to 25.35) | 15.84(10.66 to 22.03) | -0.34(-0.38 to -0.30) |
| Taiwan (Province of China) | 7.14(6.41 to 8.04) | 3.68(2.99 to 4.48) | -1.85(-2.55 to -1.14) | 21.73(20.05 to 23.26) | 7.29(6.29 to 8.19) | -3.50(-4.16 to -2.83) |
| Tajikistan | 14.92(10.06 to 21.00) | 14.76(9.61 to 21.92) | -0.20(-0.41 to 0.01) | 21.73(13.11 to 34.50) | 16.49(10.77 to 25.45) | -0.63(-0.81 to -0.46) |
| Thailand | 1.85(1.25 to 2.59) | 2.37(1.67 to 3.19) | 0.82(0.70 to 0.93) | 2.57(1.81 to 3.80) | 2.69(1.83 to 3.65) | 0.08(-0.05 to 0.22) |
| Timor-Leste | 3.54(1.56 to 6.87) | 3.51(1.96 to 6.20) | -0.23(-0.69 to 0.23) | 7.97(2.69 to 14.78) | 6.66(3.15 to 11.89) | -0.75(-0.96 to -0.54) |
| Togo | 61.45(34.24 to 100.54) | 77.86(37.64 to 131.63) | 0.88(0.79 to 0.97) | 36.78(21.55 to 59.54) | 42.68(21.52 to 70.94) | 0.52(0.44 to 0.60) |
| Tokelau | 2.80(1.86 to 3.97) | 2.83(1.93 to 3.95) | -0.27(-0.39 to -0.14) | 3.73(2.49 to 5.27) | 4.67(3.04 to 7.60) | -0.38(-0.80 to 0.05) |
| Tonga | 2.63(1.81 to 3.74) | 2.78(1.80 to 4.55) | 0.24(0.20 to 0.29) | 4.00(2.58 to 5.97) | 3.78(2.43 to 6.00) | -0.15(-0.22 to -0.08) |
| Trinidad and Tobago | 16.75(14.52 to 19.82) | 17.46(13.40 to 22.62) | -0.07(-0.30 to 0.17) | 19.41(17.16 to 21.88) | 14.80(11.79 to 18.43) | -1.03(-1.26 to -0.79) |
| Tunisia | 7.02(4.42 to 10.98) | 9.67(6.26 to 14.10) | 1.16(1.09 to 1.23) | 8.56(5.19 to 14.70) | 9.20(6.15 to 13.85) | 0.44(0.32 to 0.56) |
| Türkiye | 15.41(9.78 to 23.58) | 12.01(8.52 to 16.36) | 0.08(-0.02 to 0.17) | 18.78(12.33 to 28.57) | 14.16(10.66 to 18.39) | -0.81(-0.89 to -0.74) |
| Turkmenistan | 16.71(12.04 to 21.02) | 17.65(11.96 to 24.87) | -1.13(-1.31 to -0.95) | 19.17(13.64 to 23.36) | 17.37(12.54 to 24.02) | -0.40(-0.49 to -0.31) |
| Tuvalu | 3.33(2.17 to 5.02) | 2.51(1.71 to 3.63) | -0.71(-0.81 to -0.61) | 3.80(2.40 to 5.60) | 3.04(2.03 to 4.37) | -0.72(-0.77 to -0.66) |
| Uganda | 4.04(2.48 to 6.61) | 5.11(3.43 to 7.40) | 0.34(0.14 to 0.54) | 6.71(4.16 to 10.12) | 7.45(5.25 to 11.01) | 0.12(-0.01 to 0.25) |
| Ukraine | 13.14(10.49 to 16.02) | 15.80(10.54 to 21.32) | 0.27(0.08 to 0.46) | 14.58(11.97 to 17.27) | 13.36(9.60 to 17.31) | -0.42(-0.56 to -0.29) |
| United Arab Emirates | 9.82(6.36 to 13.97) | 13.02(7.96 to 20.10) | 1.07(0.89 to 1.26) | 10.50(7.24 to 13.99) | 12.51(8.94 to 17.45) | 1.21(0.96 to 1.46) |
| United Kingdom | 33.01(24.55 to 43.79) | 35.35(25.79 to 47.09) | -0.20(-0.46 to 0.06) | 41.24(34.90 to 49.13) | 43.19(35.92 to 51.58) | 0.01(-0.31 to 0.33) |
| United Republic of Tanzania | 6.46(4.11 to 9.20) | 7.59(4.70 to 11.68) | 0.57(0.41 to 0.74) | 10.27(7.16 to 14.64) | 11.01(7.02 to 16.70) | 0.38(0.26 to 0.51) |
| United States of America | 35.09(24.50 to 47.97) | 38.67(28.53 to 51.77) | 0.34(0.12 to 0.55) | 40.30(31.51 to 50.74) | 49.57(41.28 to 59.80) | 0.90(0.69 to 1.12) |
| United States Virgin Islands | 30.05(20.43 to 40.07) | 20.44(12.48 to 31.38) | -0.98(-1.23 to -0.74) | 33.33(21.71 to 43.02) | 17.20(10.93 to 25.81) | -2.01(-2.23 to -1.79) |
| Uruguay | 17.99(13.97 to 23.29) | 16.44(12.64 to 21.54) | -0.38(-0.47 to -0.29) | 20.15(16.89 to 24.11) | 17.11(14.28 to 20.84) | -0.59(-0.67 to -0.51) |
| Uzbekistan | 12.50(9.70 to 16.23) | 14.81(11.15 to 19.33) | 0.31(0.03 to 0.58) | 15.37(12.31 to 19.38) | 15.04(11.87 to 18.78) | -0.23(-0.52 to 0.05) |
| Vanuatu | 2.44(1.43 to 4.03) | 2.92(1.71 to 4.93) | 0.36(0.23 to 0.48) | 3.39(1.84 to 5.65) | 3.42(1.87 to 5.43) | -0.04(-0.17 to 0.09) |
| Venezuela (Bolivarian Republic of) | 4.61(3.97 to 5.42) | 4.98(3.59 to 6.58) | 0.51(0.25 to 0.78) | 6.52(5.81 to 7.28) | 5.31(3.95 to 6.94) | -0.25(-0.52 to 0.03) |
| Viet Nam | 2.44(1.49 to 3.56) | 2.21(1.46 to 3.20) | -0.35(-0.49 to -0.20) | 4.82(2.45 to 7.55) | 3.20(2.06 to 4.78) | -1.40(-1.53 to -1.28) |
| Yemen | 5.56(3.46 to 8.89) | 7.59(5.07 to 10.79) | 1.24(1.07 to 1.42) | 7.79(4.48 to 14.22) | 9.70(6.17 to 15.56) | 0.93(0.74 to 1.13) |
| Zambia | 7.83(4.36 to 13.01) | 8.27(5.01 to 12.75) | 0.11(0.04 to 0.18) | 11.51(7.51 to 18.18) | 11.13(7.09 to 16.54) | -0.12(-0.21 to -0.04) |
| Zimbabwe | 5.30(3.32 to 8.82) | 8.20(4.12 to 13.62) | 1.84(1.44 to 2.24) | 10.06(7.00 to 14.87) | 13.07(6.96 to 21.52) | 1.27(0.88 to 1.65) |
| **Abbreviations:** IBD, inflammatory bowel disease; AS, age-standardized; WCBA, women of childbearing age; DALYs, disability-adjusted life-years; EAPC, estimated annual percentage change; CI, Confidence Interval. | | | | | | |
