# Supplementary material for: Global disease burden of inflammatory bowel disease in women and women of childbearing age from 1990 to 2021 and its prediction to 2040
Source: PLoS One. 2025 Sep 10;20(9):e0331034. doi: 10.1371/journal.pone.0331034 (PMC12422439; doi:10.1371/journal.pone.0331034)
Supplement: S6 Table — Abbreviations: IBD, inflammatory bowel disease; AS, age-standardized; WCBA, women of childbearing age; EAPC, estimated annual percentage change; CI, Confidence Interval. (DOCX) [file pone.0331034.s009.docx]

Table S6 The mortality rate for IBD among women and WCBA and its temporal trends from 1990 to 2021 across 204 countries and regions

| Location | Mortality rate of WCBA _1990 | Mortality rate of WCBA _2021 | EAPC_CI | AS mortality rate of women_1990 | AS mortality rate of women _2021 | EAPC_CI |
| --- | --- | --- | --- | --- | --- | --- |
| Afghanistan | 0.12(0.02 to 0.51) | 0.13(0.02 to 0.46) | 0.47(0.28 to 0.66) | 0.39(0.14 to 1.12) | 0.41(0.14 to 0.98) | 0.41(0.26 to 0.57) |
| Albania | 0.24(0.15 to 0.38) | 0.14(0.07 to 0.23) | -1.28(-1.57 to -1.00) | 0.89(0.57 to 1.42) | 0.54(0.29 to 0.95) | -1.33(-1.51 to -1.16) |
| Algeria | 0.05(0.01 to 0.15) | 0.04(0.02 to 0.12) | -0.03(-0.19 to 0.13) | 0.18(0.07 to 0.45) | 0.16(0.08 to 0.39) | 0.46(0.17 to 0.76) |
| American Samoa | 0.03(0.01 to 0.05) | 0.05(0.03 to 0.09) | 3.87(2.83 to 4.92) | 0.11(0.06 to 0.19) | 0.14(0.08 to 0.23) | 2.24(1.37 to 3.11) |
| Andorra | 0.09(0.05 to 0.14) | 0.07(0.03 to 0.13) | 0.06(-0.17 to 0.29) | 0.67(0.36 to 1.08) | 0.50(0.26 to 0.80) | -0.58(-0.76 to -0.39) |
| Angola | 0.10(0.02 to 0.21) | 0.09(0.03 to 0.15) | -0.10(-0.31 to 0.12) | 0.28(0.08 to 0.52) | 0.24(0.12 to 0.41) | -0.45(-0.53 to -0.36) |
| Antigua and Barbuda | 0.20(0.17 to 0.24) | 0.08(0.07 to 0.10) | -2.43(-2.76 to -2.10) | 0.52(0.45 to 0.59) | 0.26(0.24 to 0.29) | -2.55(-2.93 to -2.17) |
| Argentina | 0.09(0.08 to 0.10) | 0.06(0.06 to 0.07) | -1.00(-1.34 to -0.66) | 0.28(0.25 to 0.31) | 0.21(0.19 to 0.23) | -0.72(-1.01 to -0.42) |
| Armenia | 0.13(0.08 to 0.16) | 0.08(0.06 to 0.09) | -1.46(-1.85 to -1.06) | 0.25(0.19 to 0.29) | 0.26(0.21 to 0.30) | 0.14(-0.39 to 0.67) |
| Australia | 0.04(0.04 to 0.05) | 0.10(0.09 to 0.12) | 3.64(2.69 to 4.60) | 0.30(0.27 to 0.34) | 0.86(0.70 to 0.99) | 4.33(3.35 to 5.32) |
| Austria | 0.09(0.08 to 0.10) | 0.07(0.06 to 0.08) | -0.04(-0.62 to 0.55) | 0.65(0.58 to 0.71) | 0.46(0.38 to 0.51) | -0.34(-1.06 to 0.39) |
| Azerbaijan | 0.05(0.03 to 0.08) | 0.03(0.01 to 0.05) | -1.70(-1.85 to -1.56) | 0.09(0.05 to 0.15) | 0.06(0.03 to 0.11) | -1.46(-1.52 to -1.39) |
| Bahamas | 0.38(0.30 to 0.45) | 0.24(0.18 to 0.31) | -1.73(-2.09 to -1.38) | 0.79(0.66 to 0.89) | 0.42(0.34 to 0.52) | -1.94(-2.34 to -1.55) |
| Bahrain | 0.04(0.02 to 0.10) | 0.03(0.01 to 0.08) | -0.62(-0.79 to -0.46) | 0.22(0.08 to 0.58) | 0.18(0.09 to 0.39) | -1.09(-1.36 to -0.82) |
| Bangladesh | 0.15(0.06 to 0.26) | 0.13(0.07 to 0.20) | -0.21(-0.40 to -0.02) | 0.57(0.32 to 0.83) | 0.38(0.22 to 0.59) | -1.35(-1.49 to -1.21) |
| Barbados | 0.23(0.20 to 0.26) | 0.21(0.16 to 0.28) | -0.16(-0.59 to 0.27) | 0.54(0.49 to 0.59) | 0.52(0.41 to 0.65) | 0.30(-0.15 to 0.77) |
| Belarus | 0.09(0.07 to 0.13) | 0.10(0.07 to 0.15) | 0.04(-0.33 to 0.40) | 0.29(0.26 to 0.33) | 0.29(0.24 to 0.37) | 0.14(-0.10 to 0.37) |
| Belgium | 0.16(0.14 to 0.17) | 0.15(0.13 to 0.17) | 0.27(-0.37 to 0.91) | 0.87(0.75 to 0.96) | 0.72(0.60 to 0.81) | -0.54(-1.61 to 0.54) |
| Belize | 0.16(0.13 to 0.21) | 0.11(0.09 to 0.13) | -1.47(-1.97 to -0.97) | 0.47(0.39 to 0.56) | 0.29(0.25 to 0.33) | -2.01(-2.43 to -1.58) |
| Benin | 0.64(0.30 to 1.08) | 0.87(0.37 to 1.54) | 0.73(0.55 to 0.92) | 0.41(0.21 to 0.66) | 0.51(0.22 to 0.90) | 0.61(0.45 to 0.78) |
| Bermuda | 0.33(0.26 to 0.40) | 0.08(0.06 to 0.10) | -5.22(-5.65 to -4.78) | 1.04(0.85 to 1.18) | 0.20(0.16 to 0.24) | -5.96(-6.49 to -5.42) |
| Bhutan | 0.15(0.05 to 0.33) | 0.13(0.07 to 0.25) | -0.81(-0.94 to -0.68) | 0.45(0.22 to 0.77) | 0.40(0.25 to 0.63) | -0.49(-0.56 to -0.42) |
| Bolivia (Plurinational State of) | 0.08(0.03 to 0.16) | 0.06(0.03 to 0.10) | -1.50(-1.59 to -1.40) | 0.28(0.12 to 0.51) | 0.21(0.13 to 0.33) | -1.07(-1.14 to -1.01) |
| Bosnia and Herzegovina | 0.18(0.11 to 0.28) | 0.11(0.06 to 0.19) | -1.42(-1.63 to -1.20) | 0.59(0.36 to 0.95) | 0.38(0.23 to 0.60) | -1.35(-1.59 to -1.11) |
| Botswana | 0.11(0.05 to 0.22) | 0.08(0.04 to 0.15) | -0.24(-0.92 to 0.44) | 0.45(0.26 to 0.70) | 0.32(0.20 to 0.46) | -0.70(-1.11 to -0.29) |
| Brazil | 0.19(0.18 to 0.20) | 0.23(0.21 to 0.25) | 0.66(0.37 to 0.96) | 0.47(0.44 to 0.50) | 0.51(0.46 to 0.55) | 0.44(0.13 to 0.75) |
| Brunei Darussalam | 0.26(0.14 to 0.45) | 0.19(0.11 to 0.32) | -0.41(-0.66 to -0.16) | 1.23(0.72 to 1.85) | 0.90(0.61 to 1.31) | -0.17(-0.48 to 0.14) |
| Bulgaria | 0.05(0.04 to 0.06) | 0.06(0.04 to 0.07) | 1.38(0.77 to 1.98) | 0.12(0.11 to 0.13) | 0.15(0.12 to 0.18) | 1.15(0.62 to 1.69) |
| Burkina Faso | 0.64(0.37 to 1.00) | 0.77(0.36 to 1.29) | 0.76(0.46 to 1.06) | 0.41(0.24 to 0.61) | 0.47(0.22 to 0.75) | 0.62(0.34 to 0.90) |
| Burundi | 0.10(0.04 to 0.19) | 0.09(0.04 to 0.15) | -0.92(-1.09 to -0.74) | 0.35(0.18 to 0.56) | 0.31(0.16 to 0.53) | -0.72(-0.85 to -0.59) |
| Cabo Verde | 0.86(0.48 to 1.32) | 0.50(0.25 to 0.86) | -2.09(-2.22 to -1.97) | 0.52(0.30 to 0.79) | 0.28(0.15 to 0.46) | -2.21(-2.30 to -2.13) |
| Cambodia | 0.11(0.03 to 0.23) | 0.09(0.04 to 0.17) | -0.98(-1.11 to -0.86) | 0.56(0.21 to 1.04) | 0.39(0.20 to 0.64) | -1.41(-1.51 to -1.32) |
| Cameroon | 0.74(0.39 to 1.19) | 0.83(0.38 to 1.44) | 0.31(0.21 to 0.41) | 0.46(0.25 to 0.73) | 0.48(0.22 to 0.83) | 0.03(-0.08 to 0.14) |
| Canada | 0.10(0.09 to 0.11) | 0.13(0.12 to 0.15) | 1.05(0.65 to 1.45) | 0.84(0.73 to 0.91) | 0.53(0.46 to 0.59) | -2.07(-2.72 to -1.41) |
| Central African Republic | 0.09(0.03 to 0.18) | 0.11(0.05 to 0.21) | 0.74(0.62 to 0.85) | 0.25(0.11 to 0.44) | 0.31(0.17 to 0.53) | 0.75(0.58 to 0.93) |
| Chad | 0.68(0.31 to 1.32) | 0.90(0.42 to 1.62) | 1.06(0.93 to 1.18) | 0.44(0.20 to 0.82) | 0.58(0.28 to 1.02) | 1.04(0.91 to 1.17) |
| Chile | 0.12(0.11 to 0.13) | 0.07(0.06 to 0.07) | -1.82(-2.29 to -1.36) | 0.45(0.41 to 0.49) | 0.25(0.22 to 0.28) | -1.82(-2.26 to -1.39) |
| China | 0.08(0.03 to 0.13) | 0.03(0.02 to 0.06) | -3.02(-3.24 to -2.80) | 0.74(0.36 to 1.03) | 0.25(0.16 to 0.43) | -3.95(-4.36 to -3.53) |
| Colombia | 0.08(0.07 to 0.09) | 0.08(0.06 to 0.09) | 0.55(0.22 to 0.88) | 0.28(0.26 to 0.31) | 0.23(0.19 to 0.27) | -0.11(-0.39 to 0.17) |
| Comoros | 0.13(0.06 to 0.23) | 0.13(0.08 to 0.20) | -0.59(-1.07 to -0.11) | 0.39(0.24 to 0.59) | 0.37(0.23 to 0.61) | -0.39(-0.51 to -0.28) |
| Congo | 0.08(0.02 to 0.18) | 0.11(0.06 to 0.23) | 1.22(0.97 to 1.46) | 0.26(0.09 to 0.49) | 0.29(0.16 to 0.50) | 0.44(0.21 to 0.66) |
| Cook Islands | 0.02(0.01 to 0.04) | 0.02(0.01 to 0.04) | -0.15(-0.39 to 0.10) | 0.05(0.03 to 0.09) | 0.03(0.01 to 0.07) | -1.49(-1.65 to -1.34) |
| Costa Rica | 0.04(0.03 to 0.04) | 0.05(0.04 to 0.06) | 0.90(0.50 to 1.31) | 0.14(0.12 to 0.15) | 0.15(0.13 to 0.17) | 0.17(-0.08 to 0.42) |
| Côte d'Ivoire | 0.58(0.30 to 1.02) | 0.91(0.39 to 1.63) | -0.53(-1.09 to 0.02) | 0.37(0.20 to 0.64) | 0.51(0.23 to 0.89) | 1.45(1.28 to 1.61) |
| Croatia | 0.08(0.07 to 0.10) | 0.06(0.05 to 0.08) | -3.34(-3.64 to -3.03) | 0.28(0.25 to 0.30) | 0.30(0.25 to 0.35) | 0.76(0.34 to 1.18) |
| Cuba | 0.16(0.14 to 0.21) | 0.06(0.05 to 0.08) | -2.34(-2.54 to -2.14) | 0.41(0.35 to 0.49) | 0.16(0.13 to 0.19) | -3.23(-3.56 to -2.89) |
| Cyprus | 0.25(0.13 to 0.41) | 0.13(0.08 to 0.24) | -0.44(-0.80 to -0.07) | 3.33(1.81 to 5.59) | 1.37(0.88 to 1.94) | -3.17(-3.50 to -2.84) |
| Czechia | 0.17(0.14 to 0.19) | 0.14(0.11 to 0.18) | 1.86(1.70 to 2.03) | 0.48(0.41 to 0.55) | 0.49(0.40 to 0.58) | 0.67(0.13 to 1.21) |
| Democratic People's Republic of Korea | 0.07(0.03 to 0.14) | 0.07(0.03 to 0.13) | -0.06(-0.14 to 0.03) | 0.59(0.30 to 1.03) | 0.45(0.25 to 0.79) | -0.88(-0.96 to -0.80) |
| Democratic Republic of the Congo | 0.09(0.04 to 0.16) | 0.09(0.04 to 0.15) | -0.32(-0.52 to -0.12) | 0.24(0.13 to 0.39) | 0.23(0.13 to 0.41) | -0.10(-0.14 to -0.06) |
| Denmark | 0.10(0.09 to 0.11) | 0.07(0.06 to 0.09) | -0.77(-1.31 to -0.23) | 0.43(0.40 to 0.47) | 0.49(0.42 to 0.55) | 0.61(-0.24 to 1.48) |
| Djibouti | 0.09(0.04 to 0.16) | 0.09(0.04 to 0.15) | -0.27(-0.51 to -0.03) | 0.35(0.21 to 0.54) | 0.29(0.17 to 0.51) | -0.67(-0.79 to -0.55) |
| Dominica | 0.09(0.05 to 0.12) | 0.09(0.05 to 0.14) | 0.12(-0.06 to 0.29) | 0.28(0.18 to 0.38) | 0.22(0.15 to 0.33) | -1.00(-1.07 to -0.94) |
| Dominican Republic | 0.08(0.05 to 0.12) | 0.07(0.04 to 0.11) | -0.59(-0.82 to -0.35) | 0.21(0.15 to 0.33) | 0.14(0.09 to 0.23) | -1.45(-1.58 to -1.31) |
| Ecuador | 0.07(0.06 to 0.08) | 0.03(0.02 to 0.03) | -3.09(-3.49 to -2.69) | 0.24(0.21 to 0.26) | 0.11(0.09 to 0.13) | -2.22(-2.68 to -1.75) |
| Egypt | 0.10(0.06 to 0.16) | 0.09(0.05 to 0.15) | -1.36(-1.73 to -0.99) | 0.50(0.33 to 0.74) | 0.47(0.29 to 0.81) | -0.38(-0.63 to -0.13) |
| El Salvador | 0.07(0.04 to 0.10) | 0.06(0.03 to 0.09) | -0.52(-0.71 to -0.34) | 0.24(0.16 to 0.34) | 0.17(0.11 to 0.26) | -0.76(-1.02 to -0.49) |
| Equatorial Guinea | 0.13(0.05 to 0.25) | 0.10(0.04 to 0.19) | -1.26(-1.51 to -1.01) | 0.33(0.17 to 0.53) | 0.26(0.11 to 0.48) | -0.93(-1.15 to -0.70) |
| Eritrea | 0.12(0.05 to 0.24) | 0.13(0.07 to 0.22) | 0.20(0.09 to 0.32) | 0.42(0.23 to 0.63) | 0.42(0.28 to 0.65) | -0.02(-0.13 to 0.08) |
| Estonia | 0.30(0.26 to 0.38) | 0.05(0.04 to 0.06) | -7.26(-9.16 to -5.32) | 1.09(0.97 to 1.22) | 0.19(0.16 to 0.22) | -6.59(-8.35 to -4.80) |
| Eswatini | 0.10(0.05 to 0.19) | 0.12(0.05 to 0.22) | 0.76(0.35 to 1.17) | 0.43(0.25 to 0.73) | 0.42(0.25 to 0.68) | 0.27(-0.03 to 0.56) |
| Ethiopia | 0.09(0.04 to 0.19) | 0.07(0.04 to 0.12) | -1.21(-1.38 to -1.03) | 0.33(0.21 to 0.56) | 0.26(0.15 to 0.46) | -1.09(-1.26 to -0.93) |
| Fiji | 0.03(0.02 to 0.05) | 0.03(0.01 to 0.05) | -0.41(-0.64 to -0.18) | 0.09(0.05 to 0.15) | 0.07(0.04 to 0.12) | -0.62(-0.86 to -0.39) |
| Finland | 0.04(0.04 to 0.05) | 0.04(0.04 to 0.05) | 0.44(-0.27 to 1.15) | 0.32(0.27 to 0.36) | 0.23(0.19 to 0.26) | -0.44(-0.87 to -0.02) |
| France | 0.15(0.14 to 0.17) | 0.16(0.14 to 0.18) | 0.82(0.28 to 1.35) | 1.07(0.94 to 1.17) | 1.31(1.06 to 1.51) | 1.19(0.77 to 1.61) |
| Gabon | 0.07(0.02 to 0.14) | 0.08(0.03 to 0.15) | 0.38(0.09 to 0.66) | 0.22(0.08 to 0.39) | 0.23(0.12 to 0.41) | 0.20(-0.04 to 0.44) |
| Gambia | 0.84(0.40 to 1.51) | 1.30(0.65 to 2.22) | 1.24(0.97 to 1.50) | 0.55(0.28 to 0.96) | 0.77(0.41 to 1.32) | 0.90(0.65 to 1.16) |
| Georgia | 0.08(0.05 to 0.12) | 0.09(0.07 to 0.12) | -0.64(-1.83 to 0.57) | 0.15(0.10 to 0.22) | 0.21(0.17 to 0.26) | 0.38(-0.86 to 1.64) |
| Germany | 0.22(0.19 to 0.24) | 0.23(0.20 to 0.26) | 0.89(0.47 to 1.32) | 0.81(0.70 to 0.92) | 1.90(1.55 to 2.14) | 4.22(3.57 to 4.87) |
| Ghana | 0.73(0.40 to 1.12) | 0.71(0.32 to 1.34) | -0.20(-0.29 to -0.11) | 0.43(0.23 to 0.65) | 0.40(0.18 to 0.72) | -0.27(-0.35 to -0.19) |
| Greece | 0.02(0.02 to 0.02) | 0.03(0.03 to 0.04) | 2.56(2.03 to 3.08) | 0.21(0.19 to 0.24) | 0.27(0.22 to 0.30) | 1.18(0.80 to 1.55) |
| Greenland | 0.16(0.09 to 0.36) | 0.14(0.08 to 0.25) | -0.53(-0.79 to -0.27) | 0.95(0.52 to 1.27) | 0.91(0.29 to 1.48) | 0.51(0.18 to 0.83) |
| Grenada | 0.52(0.40 to 0.63) | 0.29(0.24 to 0.37) | -1.98(-2.27 to -1.70) | 1.15(0.88 to 1.38) | 0.73(0.63 to 0.83) | -1.49(-1.76 to -1.22) |
| Guam | 0.01(0.00 to 0.02) | 0.02(0.01 to 0.02) | 4.24(2.91 to 5.58) | 0.05(0.02 to 0.12) | 0.03(0.02 to 0.05) | 0.96(-0.44 to 2.39) |
| Guatemala | 0.12(0.09 to 0.13) | 0.09(0.07 to 0.10) | -0.83(-1.42 to -0.24) | 0.31(0.25 to 0.35) | 0.24(0.21 to 0.28) | -0.56(-0.95 to -0.16) |
| Guinea | 0.64(0.25 to 1.25) | 0.87(0.43 to 1.43) | 1.00(0.88 to 1.12) | 0.38(0.15 to 0.73) | 0.51(0.25 to 0.83) | 0.92(0.75 to 1.08) |
| Guinea-Bissau | 1.08(0.44 to 2.10) | 1.50(0.80 to 2.47) | 1.16(1.05 to 1.27) | 0.67(0.28 to 1.30) | 0.85(0.46 to 1.40) | 0.88(0.71 to 1.06) |
| Guyana | 0.37(0.27 to 0.50) | 0.42(0.29 to 0.59) | 0.10(-0.28 to 0.49) | 1.09(0.92 to 1.26) | 0.83(0.63 to 1.07) | -0.68(-1.09 to -0.26) |
| Haiti | 0.44(0.13 to 0.87) | 0.39(0.15 to 0.75) | -0.33(-0.45 to -0.21) | 1.16(0.41 to 2.32) | 0.97(0.41 to 1.86) | -0.46(-0.53 to -0.39) |
| Honduras | 0.20(0.08 to 0.33) | 0.14(0.06 to 0.26) | -1.23(-1.35 to -1.12) | 0.49(0.24 to 0.71) | 0.40(0.24 to 0.71) | -0.60(-0.69 to -0.52) |
| Hungary | 0.24(0.21 to 0.27) | 0.22(0.17 to 0.27) | -0.81(-1.21 to -0.42) | 0.56(0.50 to 0.63) | 0.58(0.49 to 0.68) | 0.18(-0.30 to 0.65) |
| Iceland | 0.15(0.13 to 0.17) | 0.18(0.15 to 0.21) | 0.82(0.36 to 1.28) | 0.70(0.61 to 0.78) | 0.89(0.73 to 1.03) | 1.36(0.84 to 1.88) |
| India | 0.16(0.07 to 0.29) | 0.09(0.05 to 0.17) | -1.87(-2.10 to -1.64) | 0.41(0.21 to 0.64) | 0.28(0.17 to 0.47) | -1.26(-1.41 to -1.10) |
| Indonesia | 0.09(0.03 to 0.15) | 0.05(0.03 to 0.07) | -2.15(-2.33 to -1.97) | 0.60(0.20 to 1.06) | 0.37(0.21 to 0.52) | -1.90(-2.07 to -1.73) |
| Iran (Islamic Republic of) | 0.03(0.01 to 0.05) | 0.03(0.01 to 0.04) | 0.07(-0.34 to 0.47) | 0.12(0.05 to 0.19) | 0.11(0.04 to 0.17) | 0.08(-0.33 to 0.49) |
| Iraq | 0.15(0.07 to 0.25) | 0.10(0.05 to 0.17) | -1.34(-1.53 to -1.15) | 0.35(0.20 to 0.53) | 0.26(0.15 to 0.45) | -1.04(-1.12 to -0.96) |
| Ireland | 0.13(0.12 to 0.15) | 0.15(0.12 to 0.18) | 1.02(0.43 to 1.63) | 0.90(0.81 to 0.98) | 0.71(0.56 to 0.85) | -0.19(-0.82 to 0.45) |
| Israel | 0.04(0.03 to 0.04) | 0.03(0.03 to 0.04) | 0.71(0.13 to 1.29) | 0.33(0.28 to 0.36) | 0.33(0.26 to 0.38) | 0.51(-0.16 to 1.18) |
| Italy | 0.09(0.08 to 0.09) | 0.13(0.12 to 0.14) | 2.29(1.81 to 2.78) | 0.38(0.34 to 0.40) | 0.84(0.68 to 0.96) | 3.42(2.90 to 3.95) |
| Jamaica | 0.07(0.06 to 0.09) | 0.10(0.07 to 0.14) | 0.99(0.37 to 1.60) | 0.19(0.18 to 0.21) | 0.21(0.16 to 0.27) | 0.22(-0.22 to 0.66) |
| Japan | 0.05(0.04 to 0.05) | 0.02(0.02 to 0.02) | -2.86(-3.29 to -2.43) | 0.21(0.18 to 0.22) | 0.06(0.05 to 0.07) | -3.44(-4.18 to -2.69) |
| Jordan | 0.09(0.04 to 0.18) | 0.07(0.04 to 0.10) | -0.85(-1.10 to -0.60) | 0.38(0.20 to 0.72) | 0.31(0.18 to 0.45) | -0.31(-0.76 to 0.15) |
| Kazakhstan | 0.20(0.16 to 0.25) | 0.27(0.20 to 0.35) | 0.01(-0.44 to 0.47) | 0.42(0.36 to 0.49) | 0.42(0.32 to 0.54) | -0.69(-1.03 to -0.35) |
| Kenya | 0.09(0.04 to 0.18) | 0.10(0.05 to 0.16) | 0.47(0.35 to 0.59) | 0.36(0.17 to 0.59) | 0.35(0.19 to 0.56) | 0.38(0.24 to 0.52) |
| Kiribati | 0.03(0.01 to 0.05) | 0.03(0.01 to 0.05) | 0.64(0.43 to 0.85) | 0.09(0.04 to 0.19) | 0.10(0.05 to 0.18) | 0.29(0.18 to 0.40) |
| Kuwait | 0.01(0.01 to 0.01) | 0.04(0.03 to 0.04) | 4.34(1.44 to 7.33) | 0.03(0.02 to 0.03) | 0.16(0.13 to 0.19) | 6.37(3.47 to 9.35) |
| Kyrgyzstan | 0.24(0.19 to 0.32) | 0.08(0.06 to 0.10) | -5.32(-6.39 to -4.24) | 0.45(0.35 to 0.59) | 0.16(0.12 to 0.20) | -5.07(-6.18 to -3.95) |
| Lao People's Democratic Republic | 0.05(0.01 to 0.09) | 0.04(0.01 to 0.07) | -0.88(-0.97 to -0.80) | 0.29(0.07 to 0.56) | 0.18(0.08 to 0.32) | -1.67(-1.76 to -1.58) |
| Latvia | 0.36(0.30 to 0.43) | 0.08(0.06 to 0.10) | -6.10(-8.02 to -4.13) | 1.07(0.94 to 1.26) | 0.28(0.24 to 0.33) | -5.33(-7.15 to -3.48) |
| Lebanon | 0.13(0.05 to 0.26) | 0.07(0.05 to 0.11) | -2.02(-2.19 to -1.84) | 0.42(0.21 to 0.76) | 0.26(0.17 to 0.39) | -1.40(-1.46 to -1.33) |
| Lesotho | 0.08(0.03 to 0.20) | 0.12(0.06 to 0.20) | 2.12(1.55 to 2.68) | 0.37(0.18 to 0.74) | 0.45(0.27 to 0.73) | 1.30(0.90 to 1.70) |
| Liberia | 0.56(0.29 to 0.91) | 0.80(0.38 to 1.50) | 1.16(0.99 to 1.32) | 0.36(0.19 to 0.58) | 0.47(0.23 to 0.87) | 0.78(0.62 to 0.93) |
| Libya | 0.05(0.02 to 0.13) | 0.08(0.03 to 0.21) | 2.29(1.71 to 2.87) | 0.19(0.07 to 0.36) | 0.27(0.10 to 0.68) | 2.04(1.58 to 2.49) |
| Lithuania | 0.21(0.18 to 0.25) | 0.05(0.04 to 0.06) | -5.41(-7.22 to -3.57) | 0.65(0.57 to 0.74) | 0.20(0.17 to 0.23) | -4.58(-6.35 to -2.78) |
| Luxembourg | 0.15(0.14 to 0.17) | 0.11(0.09 to 0.12) | -0.48(-1.18 to 0.21) | 0.96(0.87 to 1.04) | 1.00(0.84 to 1.13) | 1.05(0.50 to 1.61) |
| Madagascar | 0.12(0.05 to 0.21) | 0.13(0.07 to 0.24) | 0.22(0.09 to 0.35) | 0.37(0.20 to 0.57) | 0.36(0.19 to 0.63) | -0.12(-0.17 to -0.08) |
| Malawi | 0.11(0.05 to 0.18) | 0.10(0.05 to 0.17) | -0.24(-0.34 to -0.14) | 0.34(0.22 to 0.52) | 0.33(0.19 to 0.53) | -0.11(-0.27 to 0.04) |
| Malaysia | 0.01(0.01 to 0.01) | 0.01(0.00 to 0.01) | -0.91(-1.10 to -0.71) | 0.05(0.03 to 0.07) | 0.05(0.03 to 0.07) | -0.27(-0.66 to 0.12) |
| Maldives | 0.04(0.01 to 0.08) | 0.02(0.01 to 0.04) | -1.81(-1.99 to -1.63) | 0.26(0.10 to 0.44) | 0.13(0.08 to 0.18) | -2.39(-2.58 to -2.19) |
| Mali | 1.15(0.57 to 2.05) | 1.35(0.72 to 2.14) | 0.57(0.49 to 0.66) | 0.70(0.35 to 1.26) | 0.83(0.46 to 1.29) | 0.63(0.53 to 0.73) |
| Malta | 0.09(0.08 to 0.11) | 0.12(0.10 to 0.14) | 1.56(1.09 to 2.03) | 0.54(0.48 to 0.59) | 0.63(0.52 to 0.71) | 0.80(0.26 to 1.34) |
| Marshall Islands | 0.02(0.01 to 0.04) | 0.03(0.01 to 0.06) | 0.33(0.11 to 0.54) | 0.11(0.05 to 0.19) | 0.08(0.04 to 0.14) | -0.97(-1.11 to -0.82) |
| Mauritania | 0.51(0.19 to 1.02) | 0.68(0.36 to 1.19) | 0.82(0.57 to 1.06) | 0.33(0.13 to 0.66) | 0.42(0.22 to 0.74) | 0.69(0.48 to 0.89) |
| Mauritius | 0.02(0.02 to 0.03) | 0.11(0.09 to 0.12) | 3.78(1.82 to 5.79) | 0.12(0.11 to 0.13) | 0.41(0.35 to 0.45) | 2.76(0.89 to 4.67) |
| Mexico | 0.09(0.09 to 0.10) | 0.13(0.11 to 0.15) | 1.93(1.59 to 2.26) | 0.48(0.46 to 0.49) | 0.46(0.39 to 0.52) | 0.64(0.31 to 0.98) |
| Micronesia (Federated States of) | 0.03(0.01 to 0.06) | 0.03(0.01 to 0.06) | 0.08(-0.02 to 0.17) | 0.12(0.06 to 0.21) | 0.09(0.05 to 0.15) | -0.76(-0.80 to -0.73) |
| Monaco | 0.06(0.04 to 0.09) | 0.06(0.03 to 0.10) | -0.04(-0.19 to 0.12) | 0.37(0.21 to 0.55) | 0.37(0.23 to 0.54) | 0.06(-0.05 to 0.17) |
| Mongolia | 0.18(0.09 to 0.35) | 0.13(0.06 to 0.23) | -1.86(-2.06 to -1.65) | 0.48(0.28 to 0.75) | 0.28(0.14 to 0.49) | -2.38(-2.58 to -2.19) |
| Montenegro | 0.15(0.10 to 0.22) | 0.12(0.07 to 0.18) | -0.33(-0.66 to -0.00) | 0.31(0.20 to 0.45) | 0.33(0.19 to 0.50) | 0.41(0.31 to 0.52) |
| Morocco | 0.06(0.02 to 0.17) | 0.06(0.02 to 0.15) | 0.16(-0.05 to 0.36) | 0.18(0.08 to 0.43) | 0.18(0.09 to 0.40) | 0.39(0.19 to 0.59) |
| Mozambique | 0.09(0.05 to 0.14) | 0.09(0.05 to 0.17) | 0.38(0.31 to 0.46) | 0.32(0.19 to 0.50) | 0.35(0.19 to 0.62) | 0.59(0.51 to 0.68) |
| Myanmar | 0.07(0.02 to 0.14) | 0.04(0.02 to 0.07) | -2.54(-2.74 to -2.33) | 0.22(0.07 to 0.39) | 0.11(0.06 to 0.16) | -2.73(-2.92 to -2.54) |
| Namibia | 0.12(0.05 to 0.23) | 0.10(0.04 to 0.20) | -0.75(-0.90 to -0.60) | 0.51(0.28 to 0.83) | 0.42(0.23 to 0.69) | -0.63(-0.76 to -0.51) |
| Nauru | 0.03(0.01 to 0.06) | 0.03(0.01 to 0.05) | -0.42(-0.74 to -0.11) | 0.11(0.05 to 0.18) | 0.08(0.04 to 0.16) | -0.78(-0.97 to -0.59) |
| Nepal | 0.21(0.07 to 0.43) | 0.13(0.07 to 0.22) | -1.75(-1.86 to -1.65) | 0.56(0.23 to 0.99) | 0.38(0.24 to 0.60) | -1.42(-1.57 to -1.26) |
| Netherlands | 0.29(0.26 to 0.32) | 0.27(0.24 to 0.30) | 0.03(-0.61 to 0.67) | 1.88(1.62 to 2.06) | 2.33(1.90 to 2.60) | 0.79(0.30 to 1.28) |
| New Zealand | 0.08(0.07 to 0.09) | 0.06(0.05 to 0.07) | -0.72(-1.76 to 0.34) | 0.39(0.35 to 0.42) | 0.38(0.32 to 0.43) | 0.05(-0.98 to 1.09) |
| Nicaragua | 0.04(0.03 to 0.06) | 0.04(0.02 to 0.05) | -0.12(-0.32 to 0.08) | 0.13(0.09 to 0.19) | 0.11(0.07 to 0.16) | 0.15(-0.09 to 0.39) |
| Niger | 0.67(0.28 to 1.24) | 0.79(0.34 to 1.60) | 0.32(0.15 to 0.49) | 0.42(0.18 to 0.77) | 0.50(0.22 to 0.99) | 0.31(0.16 to 0.47) |
| Nigeria | 0.47(0.23 to 0.80) | 0.56(0.20 to 1.04) | 0.58(0.46 to 0.69) | 0.32(0.16 to 0.53) | 0.36(0.14 to 0.63) | 0.41(0.33 to 0.50) |
| Niue | 0.03(0.01 to 0.06) | 0.03(0.01 to 0.06) | -0.28(-0.43 to -0.14) | 0.09(0.05 to 0.16) | 0.10(0.05 to 0.18) | -0.43(-0.72 to -0.13) |
| North Macedonia | 0.05(0.03 to 0.08) | 0.04(0.02 to 0.07) | -0.57(-0.85 to -0.29) | 0.13(0.09 to 0.19) | 0.16(0.08 to 0.23) | 0.87(0.53 to 1.22) |
| Northern Mariana Islands | 0.00(0.00 to 0.00) | 0.00(0.00 to 0.00) | 0.57(-0.67 to 1.82) | 0.01(0.01 to 0.02) | 0.01(0.00 to 0.01) | -1.51(-2.34 to -0.68) |
| Norway | 0.12(0.11 to 0.12) | 0.08(0.08 to 0.09) | -1.92(-2.68 to -1.16) | 0.32(0.29 to 0.34) | 0.37(0.31 to 0.40) | -0.47(-1.08 to 0.13) |
| Oman | 0.04(0.01 to 0.12) | 0.03(0.01 to 0.06) | -0.61(-0.85 to -0.38) | 0.14(0.06 to 0.40) | 0.10(0.04 to 0.25) | -0.43(-0.71 to -0.14) |
| Pakistan | 0.20(0.07 to 0.42) | 0.19(0.09 to 0.34) | -0.54(-0.70 to -0.37) | 0.69(0.28 to 1.43) | 0.60(0.36 to 0.90) | -0.82(-0.95 to -0.69) |
| Palau | 0.02(0.01 to 0.04) | 0.03(0.01 to 0.06) | 0.78(0.68 to 0.89) | 0.10(0.06 to 0.17) | 0.10(0.05 to 0.17) | 0.13(0.06 to 0.19) |
| Palestine | 0.23(0.12 to 0.39) | 0.10(0.06 to 0.15) | -2.82(-3.19 to -2.44) | 1.13(0.55 to 1.90) | 0.49(0.33 to 0.74) | -2.61(-2.75 to -2.48) |
| Panama | 0.16(0.14 to 0.19) | 0.18(0.14 to 0.22) | 1.00(0.68 to 1.32) | 0.49(0.43 to 0.55) | 0.54(0.42 to 0.64) | 0.79(0.54 to 1.04) |
| Papua New Guinea | 0.02(0.01 to 0.03) | 0.01(0.01 to 0.03) | -0.43(-0.71 to -0.15) | 0.05(0.02 to 0.09) | 0.04(0.02 to 0.07) | -0.85(-0.98 to -0.71) |
| Paraguay | 0.06(0.04 to 0.08) | 0.05(0.03 to 0.08) | 0.30(0.12 to 0.47) | 0.13(0.09 to 0.18) | 0.15(0.09 to 0.21) | 1.00(0.84 to 1.15) |
| Peru | 0.09(0.05 to 0.13) | 0.04(0.02 to 0.07) | -3.12(-3.55 to -2.68) | 0.28(0.16 to 0.42) | 0.13(0.08 to 0.21) | -2.89(-3.27 to -2.50) |
| Philippines | 0.04(0.02 to 0.06) | 0.03(0.02 to 0.05) | -1.19(-1.45 to -0.92) | 0.26(0.10 to 0.42) | 0.15(0.09 to 0.23) | -1.79(-2.04 to -1.53) |
| Poland | 0.14(0.13 to 0.14) | 0.11(0.10 to 0.13) | -0.57(-0.95 to -0.19) | 0.43(0.40 to 0.44) | 0.42(0.37 to 0.47) | 0.31(-0.15 to 0.77) |
| Portugal | 0.13(0.12 to 0.15) | 0.12(0.11 to 0.14) | 0.36(-0.12 to 0.83) | 0.80(0.71 to 0.89) | 0.93(0.76 to 1.05) | 1.59(0.98 to 2.20) |
| Puerto Rico | 0.24(0.21 to 0.28) | 0.11(0.09 to 0.14) | -3.53(-3.95 to -3.11) | 0.76(0.68 to 0.84) | 0.26(0.21 to 0.31) | -4.13(-4.50 to -3.75) |
| Qatar | 0.18(0.08 to 0.30) | 0.08(0.04 to 0.15) | -2.59(-2.93 to -2.25) | 1.28(0.63 to 2.01) | 0.50(0.29 to 0.76) | -3.05(-3.43 to -2.66) |
| Republic of Korea | 0.19(0.07 to 0.28) | 0.05(0.03 to 0.10) | -4.47(-4.79 to -4.14) | 1.30(0.45 to 1.95) | 0.24(0.13 to 0.57) | -6.55(-7.01 to -6.09) |
| Republic of Moldova | 0.18(0.15 to 0.22) | 0.05(0.04 to 0.05) | -5.62(-7.43 to -3.78) | 0.80(0.68 to 1.00) | 0.21(0.17 to 0.24) | -5.31(-7.36 to -3.20) |
| Romania | 0.05(0.05 to 0.06) | 0.04(0.03 to 0.05) | -0.30(-0.55 to -0.04) | 0.12(0.10 to 0.13) | 0.12(0.10 to 0.15) | 0.64(0.27 to 1.02) |
| Russian Federation | 0.20(0.18 to 0.26) | 0.21(0.18 to 0.23) | -1.16(-1.77 to -0.54) | 0.58(0.52 to 0.65) | 0.57(0.51 to 0.63) | -0.77(-1.79 to 0.26) |
| Rwanda | 0.13(0.05 to 0.24) | 0.11(0.05 to 0.20) | -0.77(-1.05 to -0.48) | 0.39(0.21 to 0.61) | 0.36(0.19 to 0.64) | -0.55(-0.71 to -0.38) |
| Saint Kitts and Nevis | 0.23(0.14 to 0.30) | 0.07(0.05 to 0.08) | -4.79(-5.27 to -4.30) | 0.57(0.42 to 0.68) | 0.21(0.18 to 0.25) | -3.17(-3.49 to -2.86) |
| Saint Lucia | 0.37(0.29 to 0.44) | 0.25(0.19 to 0.33) | -1.41(-1.83 to -0.97) | 1.14(0.94 to 1.29) | 0.56(0.44 to 0.67) | -3.23(-3.86 to -2.59) |
| Saint Vincent and the Grenadines | 0.41(0.35 to 0.48) | 0.38(0.31 to 0.47) | -0.50(-1.02 to 0.01) | 1.12(0.98 to 1.27) | 0.72(0.61 to 0.83) | -1.44(-1.88 to -1.01) |
| Samoa | 0.02(0.01 to 0.04) | 0.02(0.01 to 0.04) | 0.89(0.73 to 1.05) | 0.09(0.04 to 0.15) | 0.08(0.05 to 0.14) | -0.05(-0.09 to -0.01) |
| San Marino | 0.02(0.01 to 0.03) | 0.02(0.01 to 0.03) | 0.24(-0.18 to 0.66) | 0.37(0.23 to 0.53) | 0.20(0.10 to 0.34) | -1.50(-1.85 to -1.15) |
| Sao Tome and Principe | 0.50(0.18 to 1.10) | 0.57(0.20 to 1.41) | 0.37(-0.03 to 0.78) | 0.34(0.14 to 0.68) | 0.34(0.12 to 0.80) | -0.11(-0.45 to 0.24) |
| Saudi Arabia | 0.06(0.02 to 0.12) | 0.06(0.03 to 0.10) | -0.11(-0.55 to 0.33) | 0.19(0.09 to 0.36) | 0.14(0.07 to 0.22) | -0.93(-1.29 to -0.56) |
| Senegal | 0.89(0.48 to 1.52) | 0.92(0.48 to 1.50) | 0.22(0.05 to 0.39) | 0.56(0.30 to 0.93) | 0.54(0.29 to 0.88) | -0.06(-0.22 to 0.10) |
| Serbia | 0.24(0.16 to 0.41) | 0.16(0.09 to 0.25) | -1.04(-1.22 to -0.87) | 0.70(0.49 to 1.04) | 0.57(0.36 to 0.76) | -0.31(-0.53 to -0.10) |
| Seychelles | 0.04(0.02 to 0.05) | 0.03(0.01 to 0.05) | -0.57(-0.77 to -0.36) | 0.18(0.08 to 0.28) | 0.14(0.08 to 0.21) | -1.04(-1.25 to -0.82) |
| Sierra Leone | 0.50(0.20 to 0.93) | 0.88(0.44 to 1.42) | 1.95(1.84 to 2.06) | 0.31(0.14 to 0.55) | 0.51(0.27 to 0.80) | 1.86(1.74 to 1.97) |
| Singapore | 0.02(0.02 to 0.03) | 0.00(0.00 to 0.00) | -7.99(-8.39 to -7.60) | 0.12(0.11 to 0.13) | 0.01(0.01 to 0.02) | -7.15(-7.44 to -6.86) |
| Slovakia | 0.20(0.14 to 0.34) | 0.16(0.09 to 0.24) | -0.04(-0.24 to 0.16) | 0.54(0.40 to 0.90) | 0.52(0.30 to 0.70) | 0.43(0.16 to 0.71) |
| Slovenia | 0.14(0.13 to 0.17) | 0.07(0.05 to 0.09) | -2.14(-2.83 to -1.46) | 0.41(0.37 to 0.45) | 0.32(0.27 to 0.36) | -0.68(-1.30 to -0.06) |
| Solomon Islands | 0.01(0.00 to 0.02) | 0.01(0.01 to 0.03) | 1.76(1.67 to 1.84) | 0.05(0.02 to 0.09) | 0.05(0.02 to 0.08) | 0.16(0.06 to 0.25) |
| Somalia | 0.09(0.03 to 0.21) | 0.08(0.03 to 0.17) | -0.97(-1.18 to -0.77) | 0.31(0.17 to 0.56) | 0.28(0.16 to 0.51) | -0.41(-0.49 to -0.33) |
| South Africa | 0.13(0.07 to 0.18) | 0.10(0.05 to 0.15) | -0.24(-1.19 to 0.71) | 0.31(0.18 to 0.41) | 0.31(0.19 to 0.43) | 0.38(-0.06 to 0.81) |
| South Sudan | 0.09(0.03 to 0.19) | 0.10(0.04 to 0.19) | 0.11(-0.16 to 0.38) | 0.34(0.20 to 0.52) | 0.33(0.19 to 0.52) | -0.16(-0.24 to -0.09) |
| Spain | 0.05(0.05 to 0.05) | 0.05(0.04 to 0.05) | -0.06(-0.62 to 0.50) | 0.42(0.37 to 0.47) | 0.26(0.21 to 0.30) | -1.33(-1.72 to -0.93) |
| Sri Lanka | 0.01(0.01 to 0.02) | 0.00(0.00 to 0.01) | -3.44(-3.63 to -3.24) | 0.06(0.03 to 0.09) | 0.02(0.01 to 0.04) | -3.52(-3.69 to -3.35) |
| Sudan | 0.05(0.01 to 0.16) | 0.06(0.02 to 0.14) | 0.94(0.77 to 1.11) | 0.16(0.06 to 0.36) | 0.20(0.10 to 0.37) | 1.05(0.86 to 1.24) |
| Suriname | 0.33(0.18 to 0.46) | 0.27(0.17 to 0.45) | -0.90(-1.12 to -0.68) | 0.83(0.51 to 1.09) | 0.54(0.34 to 0.85) | -1.39(-1.52 to -1.25) |
| Sweden | 0.13(0.12 to 0.14) | 0.03(0.03 to 0.04) | -6.16(-7.53 to -4.76) | 0.77(0.67 to 0.83) | 0.39(0.32 to 0.45) | -4.30(-5.81 to -2.77) |
| Switzerland | 0.11(0.10 to 0.12) | 0.07(0.06 to 0.08) | -0.92(-1.58 to -0.25) | 0.55(0.47 to 0.61) | 0.56(0.44 to 0.63) | 0.11(-0.71 to 0.93) |
| Syrian Arab Republic | 0.13(0.06 to 0.24) | 0.10(0.05 to 0.18) | -1.00(-1.25 to -0.76) | 0.51(0.30 to 0.80) | 0.45(0.26 to 0.70) | -0.46(-0.59 to -0.33) |
| Taiwan (Province of China) | 0.11(0.10 to 0.12) | 0.04(0.04 to 0.05) | -3.14(-3.85 to -2.44) | 1.12(0.98 to 1.21) | 0.39(0.31 to 0.44) | -3.65(-4.36 to -2.94) |
| Tajikistan | 0.15(0.09 to 0.26) | 0.13(0.06 to 0.24) | -0.75(-1.02 to -0.48) | 0.33(0.20 to 0.49) | 0.23(0.13 to 0.39) | -1.03(-1.16 to -0.90) |
| Thailand | 0.01(0.01 to 0.02) | 0.02(0.01 to 0.03) | 1.02(0.75 to 1.30) | 0.07(0.04 to 0.12) | 0.08(0.04 to 0.12) | 0.23(-0.01 to 0.48) |
| Timor-Leste | 0.05(0.01 to 0.11) | 0.04(0.02 to 0.09) | -0.53(-1.13 to 0.07) | 0.31(0.08 to 0.65) | 0.25(0.10 to 0.49) | -0.86(-1.03 to -0.69) |
| Togo | 1.05(0.57 to 1.76) | 1.33(0.61 to 2.31) | 0.90(0.80 to 0.99) | 0.64(0.36 to 1.09) | 0.75(0.35 to 1.29) | 0.51(0.43 to 0.59) |
| Tokelau | 0.03(0.02 to 0.06) | 0.03(0.02 to 0.05) | -0.94(-1.17 to -0.72) | 0.12(0.06 to 0.20) | 0.10(0.05 to 0.15) | -1.31(-1.54 to -1.07) |
| Tonga | 0.03(0.02 to 0.05) | 0.03(0.01 to 0.06) | 0.15(0.07 to 0.23) | 0.13(0.06 to 0.23) | 0.12(0.06 to 0.22) | -0.15(-0.24 to -0.06) |
| Trinidad and Tobago | 0.20(0.18 to 0.23) | 0.19(0.14 to 0.27) | -0.55(-0.95 to -0.15) | 0.51(0.45 to 0.56) | 0.30(0.23 to 0.38) | -1.96(-2.31 to -1.61) |
| Tunisia | 0.04(0.01 to 0.10) | 0.03(0.01 to 0.09) | -0.14(-0.23 to -0.05) | 0.13(0.05 to 0.33) | 0.12(0.06 to 0.28) | -0.10(-0.24 to 0.05) |
| Türkiye | 0.18(0.09 to 0.31) | 0.09(0.05 to 0.13) | -0.17(-0.34 to -0.00) | 0.48(0.28 to 0.77) | 0.37(0.24 to 0.52) | -0.59(-0.85 to -0.33) |
| Turkmenistan | 0.18(0.11 to 0.24) | 0.18(0.09 to 0.31) | -1.60(-1.88 to -1.32) | 0.31(0.22 to 0.39) | 0.27(0.18 to 0.44) | -0.69(-0.88 to -0.50) |
| Tuvalu | 0.04(0.02 to 0.08) | 0.03(0.02 to 0.05) | -2.29(-2.51 to -2.08) | 0.12(0.06 to 0.21) | 0.09(0.05 to 0.16) | -0.84(-0.88 to -0.80) |
| Uganda | 0.04(0.02 to 0.09) | 0.06(0.03 to 0.09) | 0.17(-0.14 to 0.48) | 0.20(0.11 to 0.34) | 0.21(0.13 to 0.36) | -0.10(-0.28 to 0.08) |
| Ukraine | 0.14(0.12 to 0.16) | 0.19(0.11 to 0.30) | 0.36(0.03 to 0.70) | 0.27(0.23 to 0.31) | 0.26(0.17 to 0.37) | -0.38(-0.58 to -0.19) |
| United Arab Emirates | 0.03(0.01 to 0.05) | 0.02(0.01 to 0.03) | -0.94(-1.41 to -0.46) | 0.13(0.06 to 0.22) | 0.28(0.15 to 0.49) | 4.62(3.83 to 5.42) |
| United Kingdom | 0.18(0.18 to 0.19) | 0.18(0.18 to 0.19) | 0.28(-0.36 to 0.92) | 1.05(0.97 to 1.10) | 1.16(1.01 to 1.24) | 0.57(-0.02 to 1.17) |
| United Republic of Tanzania | 0.08(0.04 to 0.13) | 0.09(0.05 to 0.16) | 0.58(0.40 to 0.77) | 0.29(0.19 to 0.43) | 0.31(0.17 to 0.52) | 0.42(0.27 to 0.57) |
| United States of America | 0.12(0.12 to 0.12) | 0.21(0.20 to 0.22) | 2.03(1.64 to 2.42) | 0.66(0.59 to 0.70) | 1.08(0.94 to 1.16) | 2.05(1.59 to 2.51) |
| United States Virgin Islands | 0.44(0.25 to 0.64) | 0.23(0.09 to 0.44) | -1.60(-1.97 to -1.24) | 1.04(0.59 to 1.43) | 0.37(0.20 to 0.61) | -3.29(-3.53 to -3.04) |
| Uruguay | 0.16(0.14 to 0.18) | 0.13(0.11 to 0.14) | -0.89(-1.11 to -0.68) | 0.46(0.40 to 0.51) | 0.38(0.34 to 0.41) | -0.69(-0.85 to -0.54) |
| Uzbekistan | 0.10(0.07 to 0.12) | 0.12(0.09 to 0.15) | -0.17(-0.80 to 0.45) | 0.18(0.15 to 0.23) | 0.21(0.16 to 0.27) | -0.06(-0.61 to 0.50) |
| Vanuatu | 0.03(0.01 to 0.06) | 0.04(0.01 to 0.08) | 0.66(0.45 to 0.88) | 0.11(0.04 to 0.22) | 0.11(0.04 to 0.20) | -0.12(-0.28 to 0.04) |
| Venezuela (Bolivarian Republic of) | 0.05(0.05 to 0.06) | 0.06(0.04 to 0.09) | 0.84(0.52 to 1.16) | 0.18(0.16 to 0.20) | 0.15(0.10 to 0.20) | -0.27(-0.57 to 0.02) |
| Viet Nam | 0.02(0.01 to 0.04) | 0.01(0.01 to 0.03) | -1.64(-1.90 to -1.38) | 0.19(0.06 to 0.33) | 0.11(0.06 to 0.18) | -1.96(-2.11 to -1.81) |
| Yemen | 0.03(0.01 to 0.08) | 0.04(0.02 to 0.09) | 1.09(0.72 to 1.47) | 0.16(0.06 to 0.35) | 0.20(0.10 to 0.43) | 0.96(0.78 to 1.13) |
| Zambia | 0.11(0.05 to 0.19) | 0.11(0.05 to 0.19) | 0.02(-0.08 to 0.12) | 0.33(0.22 to 0.51) | 0.31(0.17 to 0.54) | -0.13(-0.26 to -0.01) |
| Zimbabwe | 0.06(0.03 to 0.11) | 0.11(0.04 to 0.20) | 2.85(2.16 to 3.54) | 0.33(0.21 to 0.54) | 0.42(0.21 to 0.73) | 1.31(0.82 to 1.79) |
| **Abbreviations:** IBD, inflammatory bowel disease; AS, age-standardized; WCBA, women of childbearing age; EAPC, estimated annual percentage change; CI, Confidence Interval. | | | | | | |
